# Large-Scale Modelling of the Divergent Spectrin Repeats in Nesprins: Giant Modular Proteins

**DOI:** 10.1371/journal.pone.0063633

**Published:** 2013-05-06

**Authors:** Flavia Autore, Mark Pfuhl, Xueping Quan, Aisling Williams, Roland G. Roberts, Catherine M. Shanahan, Franca Fraternali

**Affiliations:** 1 Randall Division of Cell and Molecular Biophysics, School of Physical Sciences and Engineering, King's College London, London, United Kingdom; 2 Division of Cardiovascular Medicine, BHF Centre of Research Excellence, King's College London, London, United Kingdom; 3 Division of Medical and Molecular Genetics, Kings College London, Guy's Hospital, London, United Kingdom; 4 The Thomas Young Centre for Theory and Simulation of Materials, London, United Kingdom; University of Queensland, Australia

## Abstract

Nesprin-1 and nesprin-2 are nuclear envelope (NE) proteins characterized by a common structure of an SR (spectrin repeat) rod domain and a C-terminal transmembrane KASH [Klarsicht–ANC–Syne-homology] domain and display N-terminal actin-binding CH (calponin homology) domains. Mutations in these proteins have been described in Emery-Dreifuss muscular dystrophy and attributed to disruptions of interactions at the NE with nesprins binding partners, lamin A/C and emerin. Evolutionary analysis of the rod domains of the nesprins has shown that they are almost entirely composed of unbroken SR-like structures. We present a bioinformatical approach to accurate definition of the boundaries of each SR by comparison with canonical SR structures, allowing for a large-scale homology modelling of the 74 nesprin-1 and 56 nesprin-2 SRs. The exposed and evolutionary conserved residues identify important pbs for protein-protein interactions that can guide tailored binding experiments. Most importantly, the bioinformatics analyses and the 3D models have been central to the design of selected constructs for protein expression. 1D NMR and CD spectra have been performed of the expressed SRs, showing a folded, stable, high content α-helical structure, typical of SRs. Molecular Dynamics simulations have been performed to study the structural and elastic properties of consecutive SRs, revealing insights in the mechanical properties adopted by these modules in the cell.

## Introduction

**Figure 1:**
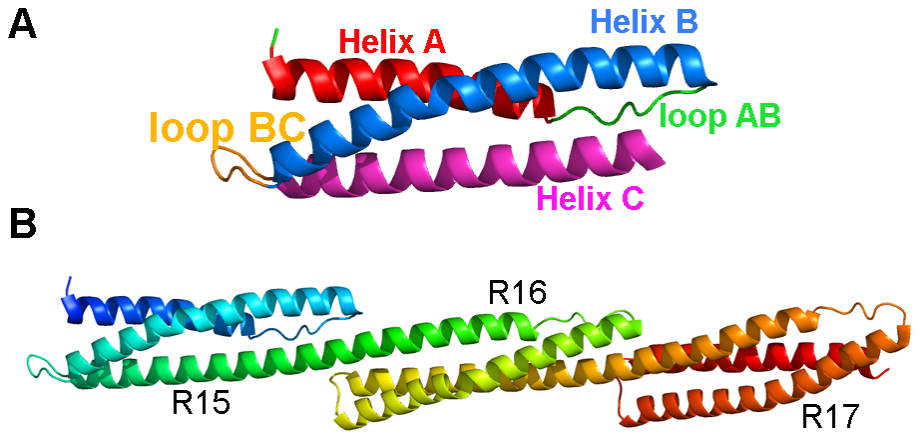
Ribbon representation of chicken brain α-spectrin crystal structures. Representation of (A) single unit SR15 and (B) the three contiguous units SR15-17 of the chicken brain α-spectrin (pdb code1U4Q).

The spectrin protein superfamily, which includes spectrin, dystrophin, α-actinin and others, is characterized by multiple repeats of a structural unit of about 100-110 residues termed ‘spectrin repeats’ (SRs) [1], [2]. Each SR consists of a characteristic motif of three bundled antiparallel α-helices (called helix A, B and C) separated by two loop regions (called loop AB and BC) (Figure 1A) [3], [4]. Within each protein, consecutive SRs are linked by a helix region (the ‘linker’) which connects the last helix of one repeat (helix C) with the first helix (helix A') of the adjacent one [5] (Figure 1B). Often previously considered as “spacers” serving merely to establish the physical separation of the functional N- and C-terminal domains of their parent protein, it is now established that SRs are not solely simple structural modules. Importantly, they are also involved in protein-protein interactions and protein dimerization and exhibit interesting and potentially functionally important mechanical attributes such as elasticity and structural flexibility.

Nesprin-1 and nesprin-2 (Nuclear Envelope SPectRIN repeat) represent the largest members of the spectrin superfamily, where the SR units comprise the vast majority of the protein backbone. The full-length nesprin-1 and -2 isoforms are giant proteins with molecular weights of 1.01 MDa and 796 kDa, respectively. Several shorter isoforms have also been identified that are mainly generated by alternative transcriptional initiation and termination [6], [7], [8], [9]

Structurally nesprin-1 and nesprin-2 are composed of three major domains: i) a pair of N-terminal CH (calponin-homology) domains that bind F-actin; ii) a C-terminal KASH (Klarsicht–ANC–Syne-homology) domain that inserts into membrane bilayers and mediates interaction with SUN-proteins (SUN1 and SUN2) at the NE; iii) an extended SR-containing rod domain separating the N- and C- terminal domains of the protein. Functionally, the CH domains of the larger outer NE nesprin isoforms connect the actin cytoskeleton to the NE via the Linker of Nucleoskeleton-and-Cytoskeleton (LINC) complex, which includes the SUN proteins and is also contiguous with the INM and the nuclear lamina. In addition, at the INM, shorter isoforms that lack the N-terminal CH domains interact with components of the lamina network (lamin A/C) and with their INM binding partner emerin [8], [10], [11], [12]

Importantly, disruption of nesprin NE interactions is involved in the etiology of the human genetic disorder Emery-Dreifuss muscular dystrophy (EDMD). EDMD can be caused either by dominant mutations in the gene encoding nuclear lamins A/C or recessive mutations in the X-linked gene encoding emerin [13]. It has also been shown that missense mutations in shorter nesprin isoforms, nesprin-1α (112 kDa) and nesprin-2β (87 kDa), can independently cause EDMD [14]. These C-terminal isoforms, which lack the CH-domains, contain the most conserved SR units which mediate both the interaction with emerin and lamins A/C [10], [11], [15] and antiparallel homodimerisation [**11**]. Taken together, these data suggest that nesprin SRs have functional activity that is dependent on specific protein-protein interactions that when disrupted can cause cellular dysfunction and disease [16], [17], [18]. Thus, the identification of the interaction interfaces and binding properties of these SRs is likely to provide essential information for our understanding of these disease processes and of the normal physiological function of the nesprins. Moreover the physical properties of nesprins, in terms of their elasticity and distensibility are likely to be integral to their function in cellular mechanical coupling.

To date, the identification of specific interaction sites and new, potentially functional, interfaces on the surface of nesprin SRs has been hampered by the lack of experimental structural data for nesprin rod domain repeats. Structural determination has also been compromised by the paucity of knowledge regarding evolutionary relationships and protein conservation between nesprins as well as the accurate definition of the single SR unit boundaries.

A recent paper [19] analysing the evolutionary conservation between nesprin genes has shown that the rod domains are composed of virtually uninterrupted SR-like structures (74 SRs in nesprin-1 and 56 in nesprin-2) ranging from poorly to very highly conserved, with the latter most probably necessary for the protein-protein interaction activity of these proteins. This useful evolutionary analysis is only the starting point for an exact determination of the SR boundaries and the design of stable constructs for structure determination. In this study we had to assess these boundaries by comparison with ‘canonical‘ SR as from Pfam database [20] and available SR structures of single, double and triple repeats (Table 1). This allowed for a large-scale comparative modelling of the 72 SRs of nesprin-1 and the 56 SRs of nesprin-2. The three-dimensional models obtained have been used to extract information on conserved and exposed surfaces, which potentially represent sites of homodimerisation or interfaces for binding interaction with known or undiscovered partners. Most importantly, this bioinformatical analysis put us in the position of designing and expressing with high yield soluble single SR domains from nesprin-1 and nesprin-2. We show by 1D NMR and CD spectra that the single SRs selected are predominantly in α-helix structure (60-80%), typical of a canonical SR domain [21], [22], [23], [24]. Lastly, Molecular Dynamics simulations on selected consecutive nesprin SRs have been performed to characterize some of the features that might influence the flexibility and other mechanical properties of the SRs.

**Table 1:** Template structures containing spectrin repeats (SR).

PDB	Molecule	number of SRs	Resolution	Reference
1HCI	Human α-actinin 2	4 SRs	2.80 Å	[62]
1SJJ	Chicken α-actinin 1	4 SRs	Ecec. Crystal.	[63]
1QUU	Human α-actinin 2	2 SRs	2.5 Å	[64]
1WLX	Human α-actinin 4	1 SR	NMR	[65]
1U4Q	Chicken α-spectrin 2	2 SRs	2.5 Å	[66]
1U5P	Chicken α–spectrin 2	2 SRs	2.0 Å	[66]
1OWA	Human α–spectrin 1	2 SRs	NMR	[67]
1CUN	Chicken α–spectrin 2	2 SRs	2.0 Å	[5]
3FB2	Human α–spectrin 2	2 SRs	2.3 Å	[68]
1AJ3	Chicken α-spectrin 2	1 SR	NMR	[4]
2SPC	Fruit fly α-spectrin	1 SR	1.8 Å	[69]
3F57	Human β-spectrin 1	2 SRs	2.9 Å	[70]
3EDU	Human β-spectrin 1	2 SRs	2.1 Å	[71]
3EDV	Human β-spectrin 2	3 SRs	1.95 Å	[72]
1S35	Human β-spectrin 1	2 SRs	2.4 Å	[37]

## Results and Discussion

### Modularity and boundaries of nesprins SRs

The recently published alignment for this class of proteins provided a useful guideline for nesprin SR boundary assignment [19]. Neverthless the exact location of these boundaries is crucial to structural determination for these important proteins largely uncharacterised. Newly defined boundaries for nesprin-1 and -2 SRs were assigned based on the SCOP assignment for canonical SR (see Methods session), which is based on their typical structural assembly and divides adjacent SRs into two triple-helix structure units (Figure S1 and S2). This, together with the SR structures selected as templates (31 single-SR structures, 17 double-SRs and 6 triple-SRs) (see Methods session), has allowed us to build models of single, double and triple nesprin SRs.

The percentage sequence identity between nesprins and the selected templates was low, with maximum values around 20%. Although sequences that share an identity below 20% can assume very different structures, it is known that evolutionarily related protein sequences accumulate substitutions to diverge into what is called ‘twilight zone’, even when their three-dimensional structure is well conserved [25]. The single SR structure extracted from all the templates was a highly conserved coiled-coil triple-helical structure, as confirmed by the low RMSD value (0.98 Å) obtained by superposing the 31 single-SR selected structures.

**Figure 2:**
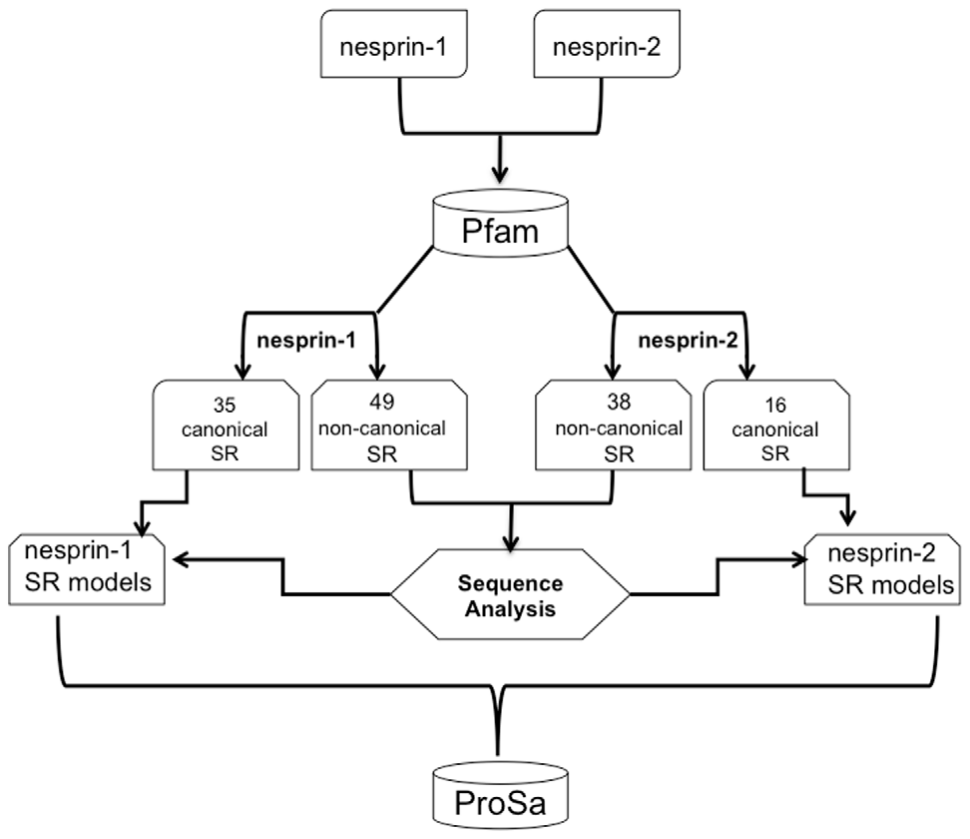
Flowchart for the nesprins SR analysis.

**Figure 3:**
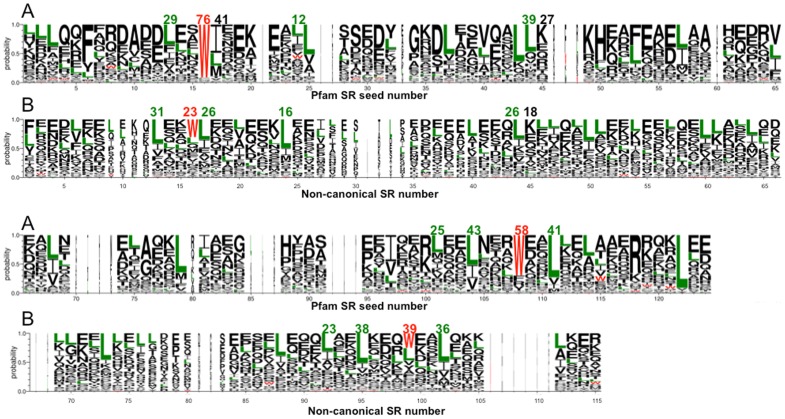
Consensus sequence for the Pfam SR seed (A) and non-canonical nesprins SRs (B). The frequency values for the conserved Trp (red) and the Leu (green) are indicated.

The sequences of nesprin-1 and -2 were analysed using Pfam [20]. This allowed classification of the nesprin SR units as ‘canonical’ (ie. those recognised as SR domains by Pfam) and as ‘non-canonical’ the remaining repeats that Pfam could not recognize as 'canonical' SRs. This analysis identified 35 canonical and 39 non-canonical in nesprin-1 and 16 canonical and 38 non-canonical in nesprin-2 (Figure 2). As nesprins are a novel class of proteins where details of the evolutionary relationship between the SRs composing the rod domain, have only recently been analysed [19], we decided to analyse the relationship of all SRs to the SR Pfam sequences in closer detail. Therefore, the consensus sequence obtained from multiple alignment of the non-canonical SRs sequences was compared with the Pfam seed alignment for SRs (Figure 3). The alignments were processed with WebLogo [26]. The sequence logo generated for the non-canonical SRs highlighted a highly conserved Leucine motif also present as a finger print in the Pfam seed for the canonical SR sequences. Moreover, also the typical Tryptophan in the helix-A highly conserved in all SRs (with a frequency of 76 in the Pfam seed) shows higher frequency when compared to the other amino acids (frequency 23 in the non-canonical SRs). This evidence further suggests the close relationship to this repeat class. In general, it has been observed before that the SR can be variable in length and in spite of low sequence identity they can nevertheless adopt the typical three helix bundle fold [27].

Having established these evolutionary relationships, a comparative modelling approach was applied to build 3-D models for the SR units as defined by Simpson and Roberts [19]. We have therefore predicted the structure of 72 single SRs, 1 double- and 2 triple-SRs of human nesprin-1 and 54 single SRs, 1 double- and 2 triple-SRs of human nesprin-2. The prediction of the nesprin-1 SRs 52 and 54 was not attempted because of their atypical length (up to twice the length of a canonical SR).

### Model Quality Evaluation

**Figure 4:**
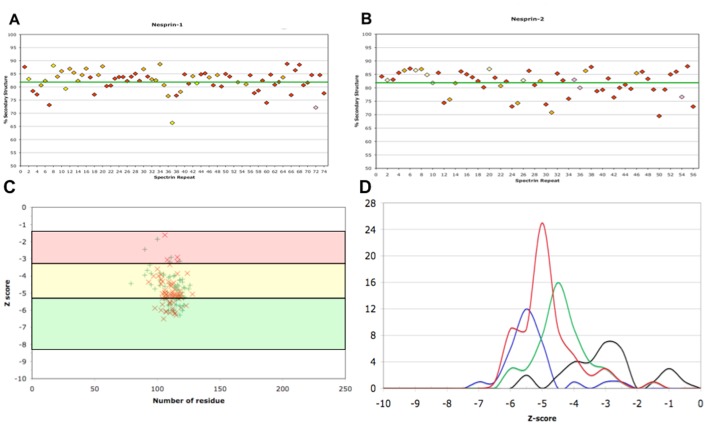
Percentage secondary structure of each SR model. (A) Nesprin-1 SR models. (B) Nesprin-2 SR models. Green lines represent the average percentage secondary structure of the templates. SR units are coloured according Simpson and Roberts [19]: red, SR confidently predicted in two or three paralogues; orange, SR confidently predicted in one paralogue; yellow, region with conserved SR-like secondary structure; pink, confidently predicted SR of atypical length. (C) PsoSA Z-score of the SRs modelled for nesprin-1 (red cross) and for nesprin-2 (green cross) SRs. (D) Zscore distributions of the templates (blue) for the three-helix bundle structure (orange) and for nesperin-1 (red) and nesprin-2 (green).

Since SRs are mostly extended helix-rich structures, evaluation of helical content is a good parameter for inspecting the quality of SR structure models. The templates used to build the model have on average 82% helical content in their structure. We chose this value as a threshold to evaluate the quality of the built SR models and decided to refine the SRs with a helical content within 10% of this value. In Figure 4 the secondary structure content for all the modelled SRs is shown. In the nesprin-1 protein three SR units diverge substantially from this cut-off value. Structure for ^NES1^SR7 (nesprin-1 SR7), ^NES1^SR37 and ^NES1^SR72 show a helical content of 73%, 66% and 72% respectively. Among these three SRs, only ^NES1^SR7 is a canonical SR while the others,^NES1^SR37 and ^NES1^SR72, are assigned as non-canonical. The same analysis performed on nesprin-2 showed that three SRs show a low helical content when compared to the selected threshold value. Two of these SRs, the ^NES2^SR30 and ^NES2^SR36 are non-canonical repeats showing a helical content of 73% and 68%, respectively. The third one, the ^NES2^SR50 is a canonical SR with 69% helical content. For the six SR outliers mentioned above, we used an alternative approach to identify the best fitting template. In this procedure, each outlier sequence was aligned against all selected template structures. This strategy resulted in a better quality model only for ^NES2^SR36. This model was in fact generated using two new templates 3FB2(SR15) and 3F57(SR14) and displayed an increased helical secondary structure content, reaching a value of 80% (Figure 4), significantly improving the helical content obtained using the model generated with the cladogram approach. For all the other outlier SRs the selected templates using this alternative strategy were identical to the ones obtained from the cladogram approach.

The repeat with the lowest value of helical content (66%) is the ^NES1^SR37 is nevertheless well within the helicity range (60% to 80%) reported for typical SRs [21], [22], [23], [24]. The models were further assessed with ProSA [28] (Table S1 and S2) and VERIFY3D [29] with results that indicate good quality models (data not shown).

To assess with precision a reliability score to the obtained models, we used the ProSA Z-score. Template structures where assessed with ProSA to extract the distribution of the template Z-score values (positive control). As a negative control, we extracted from the PDB database three structures (1LVF, 1M7K, 2P32) that are not SRs but have a closely related topology: the three-helix bundle motif (we will refer to these as THB). The sequences of these structures were extracted and several models for each sequence were predicted using the same 10 SR templates selected to model the nesprins SRs (pdb codes: 1AJ3, 10WA, 1CUN, 1HCI, 1SJJ, 2SPC, 3EDV, 1QUU, 1U5P, 3FB2). With this procedure we aimed at checking whether sequences unrelated (but with similar topology) to SRs once modelled with SRs templates would produce comparable scoring values to the ones obtained for the nesprins sequences.

For each of the THB models the ProSA score was extracted and the distribution of the scores calculated. The template distribution (canonical SR) is centred to a value of –5.3 whereas the distribution for the THB models is centred at -3 (Figure 4D). All the models with a value of Z-score smaller or equal to -5.3 were considered **very reliable models**, the models with a Z-score higher or equal to -3 wer considered **less reliable**, while the models in the range between -5.3 and -3 are assumed as **reliable models** (Figure 4C).

### Evolutionary conservation of amino acid positions in the Nesprin

Large-scale modelling was performed also aiming at the identification of potential functional regions of nesprin-1 and nesprin-2 rod domains that might be important for protein-protein interactions. In order to identify significant regions we analyzed the evolutionary conservation of amino acid positions using the ConSurf Server [30]. The Conservation score of ConSurf is defined with a scale that goes from 1 (most variable position) to 9 (most conserved position) [31]. The evolutionary conservation for each residue was calculated and projected onto the model structures. In our analysis we defined as conserved positions those residues showing a score greater than or equal to 7. The conservation analysis for all SRs was based on the alignments for each individual SR unit of vertebrate nesprin-1 and nesprin-2 separately. The alignments used were extracted from the alignment of vertebrate nesprins built by Simpson and Roberts [19].

**Figure 5:**
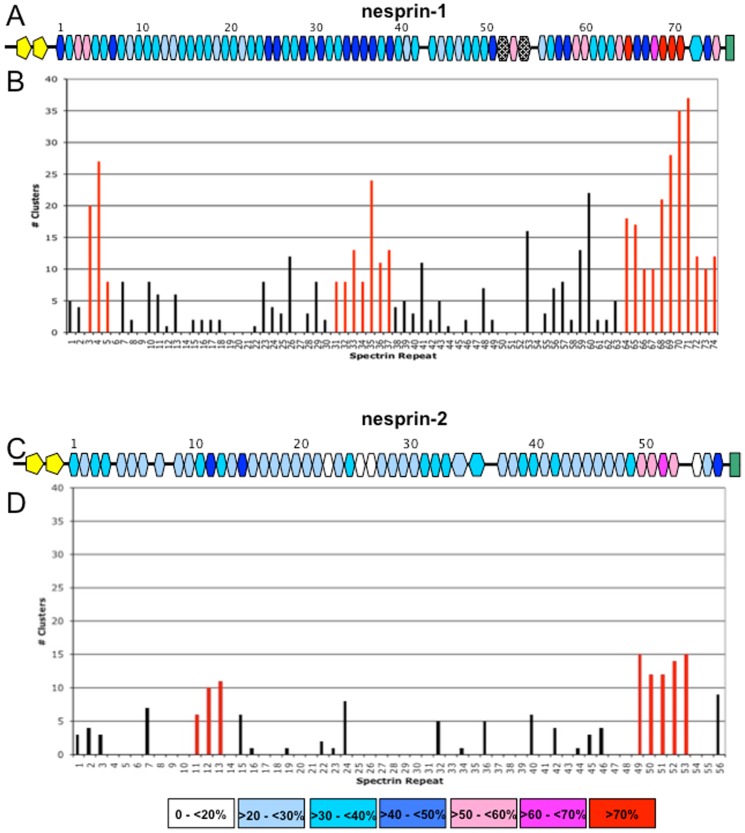
Schematic representation of the evolutionary conservation of SR units. (A) Nesprin-1. (C) Nesprin-2. Each SR unit is coloured according the percentage of conserved residues based on the alignments of vertebrate nesprin-1 and of nesprin-2, separately. The yellow star represents the invariant motif in the unstructured region. Arrows indicate N-termini of short isoforms. (B and D) Number of conservation pbs in each SR unit. Contiguous SR units with >5 pbs are coloured red for nesprin-1 (B) and for nesprin-2 (D).

The analysis of amino acid conservation performed on the nesprin-1 and nesprin-2 vertebrate alignments revealed that the vertebrate nesprin-1 SRs are evolutionarily more conserved compared to vertebrate nesprin-2, this confirms results based on sequence only [19]. In Figure 5A and C a schematic representation shows the SR units coloured according to the percent of conserved residues (conservation score ≥ 7). Along the nesprin-1 rod domain we identified two highly conserved SR units (percentage of conserved residues>50%) at the N-terminus (SR3 and SR4). The other particularly conserved SR units were clustered at the C-terminus in the regions that form the nesprin-1 short isoforms (nesprin-1β and nesprin-1α). The most conserved SRs are SR65 and the consecutive units SR69-SR71, showing a percentage of conserved residues higher than 70%. In addition to these very conserved SRs, other repeats showing a high percentage of residue conservation in this region were observed (Figure S3A and B) and these are in fact in the region of nesprin-1 which has been shown to interact with various protein partners: lamin A/C [11], emerin [11], [15], the muscle A-kinase anchoring protein (mAKAP) [32] and with MuSK, a muscle-specific tyrosine kinase of the neuromuscular junction [33]. Similarly, the two SRs at the N-terminus could represent new potential sites of interaction with other partners since they are also highly conserved among the vertebrates.

The SRs forming the rod domain of nesprin-2 are on average less conserved, however as for nesprin-1, the most conserved SRs lie near the C-terminus (SR50 to SR 53) and have also been shown to interact with emerin and lamin A, as well as the kinases ERK1/2 [8], [10], [15], [34]. The most conserved SR unit in the nesprin-2 rod domain is SR52 with 60% conserved residues.

### Identification of surface conservation pbs: detection of possible binding sites

In order to identify new interaction binding sites on the surface of the SR units we mapped exposed and evolutionary conserved residues onto the three-dimensional SR models generated. The Solvent Accessible Surface Area (SASA) of all models was computed using the POPS program [35], [36] to identify the exposed and buried residues. We defined a residue as buried when its SASA (in the protein) was lower than 20% and exposed when was higher than 20% of its SASA in the tri-peptide Ala-Xxx-Ala (isolated form), where Xxx is the considered residue. These data were merged with the conservation score information to identify those residues, which were both conserved and exposed. The percentage of buried and exposed residues among the ConSurf detected residues was also calculated. Both nesprin-1 and nesprin-2 show a similar ratio between buried and exposed residues; the general trend is that among the conserved residues, one-third is buried and the remaining two-thirds are exposed. A peculiar distribution was observed for some SRs (Figure S3C and D). The ^NES1^SR4 and ^NES1^SR5 have only 16% and 10% of the conserved residues buried, as well as ^NES1^SR33 which also had a small percentage of buried and conserved residues (10%). In nesprin-2 three SRs (^NES2^SR7, ^NES2^SR19, ^NES2^SR31) had a percentage of buried and conserved residues lower than 15%.

To find possible binding sites on the surface we analysed the clusters of conserved residues on the surface of each SR unit. An exposed and conserved residue is defined as the centre of a cluster if it shows more than five exposed and conserved neighbour residues, where neighbours are identified as the Cα atoms of exposed and conserved residues within a distance radius of less than 10Å. With this analysis we were able to map putative binding sites (pbs) onto the surface of the SR models. Along the nesprin rod domains the SR units showing a larger number of pbs were found to cluster together (Figure 5B and D). In particular in the nesprin-1 protein they were distributed in three regions of the rod domain: at the N-terminus (SR3-SR5), at the centre (SR31-SR37) and at the C-terminus (SR64-SR74) (Figure 5B). It is important to stress that the central region would have been missed by the use of ConSurf analysis alone. The ^NES1^SR31-37 fragments show a high number of pbs on their surface, despite not having a residue conservation as high as ^NES1^SR3-5 and ^NES1^SR64-74.

The same analysis performed on nesprin-2 revealed a lower number of pbs on the overall rod domain; nevertheless we were able to identify two putative regions, at the N- and C- termini, that could play an important role in protein-protein interactions, namely the C-terminal region (^NES2^SR49-53), which has been shown interact with lamin A/C and emerin as described above, and the ^NES2^SR11-SR13 region which has not yet been investigated. Some of the nesprin-2 SRs at the C-terminus were highly conserved (between 55% and 60% of conserved residues) but, unexpectedly, they presented a smaller number of pbs on the surface when compared to SRs of nesprin-1 with the same conservation profile (Figure 5D). In summary, this analysis allowed for the identification of additional potential pbs of interaction on the SR surfaces that were not identified by sequence conservation analysis alone. Thus the construction of the 3D models and their analysis has enriched substantially the available information on the conserved SR features of the nesprin family of proteins.

### Nesprin-1α and nesprin-2β: differences in binding properties

**Figure 6:**
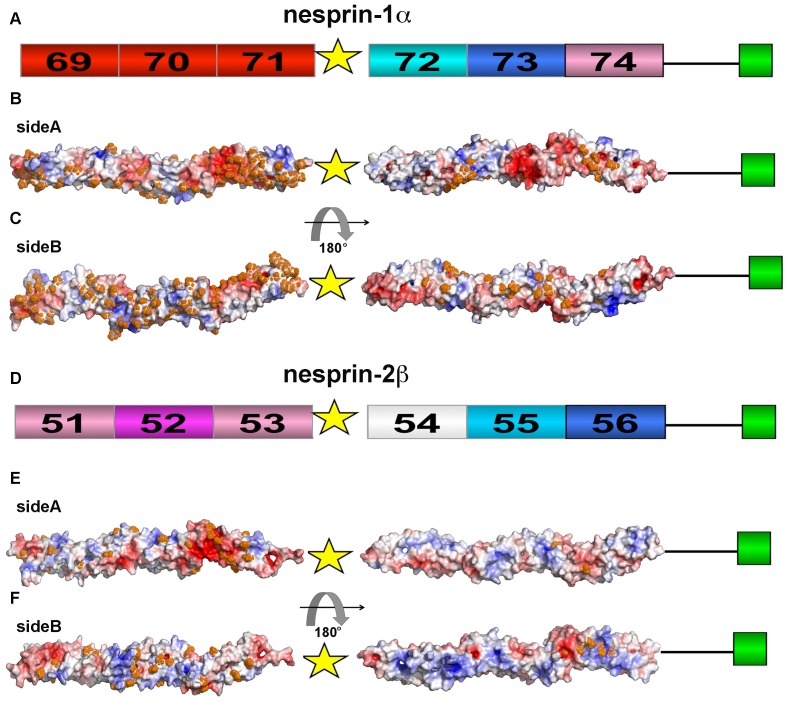
Schematic representation of nesprin-1α (A) and nesprin-2β (D); the SR units are coloured with the same colour code as in **Figure 5** **.** Panels B-E represent the electrostatic potential surfaces (face 1 and face 2) of ^NES1^SR69-71 (B), ^NES1^SR72-74 (C), ^NES2^SR51-53 (E) and ^NES2^SR54-56 (F). Blue – positive potential; red – negative potential. Residues at the centre of a conserved cluster are highlighted with orange dots.

It has been shown that nesprin-1α and nesprin-2β isoforms interact with emerin and lamin A/C [8], [10], [11], [15] and, furthermore that nesprin-1α self-associates in an antiparallel orientation [11]. Since these isoforms bind to the same partners, we decided to investigate more closely the structural features of these two proteins. In Figure 6 the domain organization of nesprin-1α and nesprin-2β is shown schematically. The two isoforms are each composed of six SR units and the C-terminal KASH domain. The first three SRs (^NES1^SR69, ^NES1^SR70, ^NES1^SR71) of nesprin-1α are highly conserved (residue conservation>70%) among vertebrate nesprin-1 proteins, showing a large number of surface pbs, wherepbsas the last three repeats (^NES1^SR72, ^NES1^SR73, ^NES1^SR74) are less conserved with a lower number of pbs. The same trend is observed in nesprin-2β, where the N-terminal SRs are more conserved when compared to the last three SRs. In addition, nesprin-2β ^NES2^SR54 and ^NES2^SR55 do not show any surface pbs.

Each isoform comprises two triple sets of SRs separated by an unstructured region (see Figure 6). The N-terminal regions of nesprin-1α (SR69-SR71) and nesprin-2β (SR51-SR53) show 76% sequence identity whereas the C-terminal ones (^NES1^SR72-SR74 and ^NES2^SR54-SR56) only 38%. This difference could be important to distinguish putative protein binding sites. To better characterize and identify potential proteins binding sites, we analysed the electrostatic surface of the triple SRs and mapped onto this the pbs determined in the conservation analysis (Figure 6). The pbs in ^NES1^SR69 and ^NES1^SR70 are clustered on the same side (Figure 6B and C), while only a few conserved residues are present on this side in ^NES1^SR71. On the other face we observed that ^NES1^SR71 has the majority of the conserved residues of the molecule grouped here. Although the number of pbs in nesprin-2β SR54-SR56 was smaller when compared to nesprin-1α SR69-SR71, we observed a similar scenario in which the ^NES2^SR51 and ^NES2^SR52 pbs are grouped on one face with the ^NES2^SR53 pbs clustered on the other face (Figure 6E and F). The positioning of pbs on the surfaces allowed the identification of a very similar conserved region in ^NES1^SR71 and ^NES2^SR53 that could be involved in binding to other proteins (and possibly their common binding partners emerin or lamin A): nesprin-1 Glu8191, Tyr8195 and Arg8202 and nesprin-2 Glu6326, Glu6327, Tyr6331 and Arg6338 (see also Table S3 and S4). Moreover, comparing these two repeats we identified an extra conserved region on ^NES1^SR71 missing on the surface of ^NES2^SR53, whose electrostatic potential matched (patches with opposite charges on the surface) with the conserved surface region in ^NES1^SR69: ^NES1^SR71 Glu8112, Thr8115, Arg8117, Asp8118, Val8122, Trp8123, Glu8126, Asp8128, Gln8130, Phe8137, Asp8141, Ala8144 and Gln 8148 (see also Table S3 and S4). Since the ^NES1^SR69 and ^NES1^SR71 units mediate the self-association of nesprin-1α we propose that we have here identified a putative surface and residues involved in this dimerization process.

The C-terminal regions of nesprin-1α and nesprin-2β show a sequence identity of 38% and indeed they display different electrostatic surfaces: the C-terminus of nesprin-1α is generally more charged then nesprin-2β.

As expected, being the C-terminal region less conserved and characterised, Pfam predicted only one SR (SR73 and SR55 for nesprin-1 and nesprin-2, respectively) in this region both for nesprin-1α and nesprin-2β, therefore we decided to proceed with further characterization involving experimental analyses.

**Figure 7:**
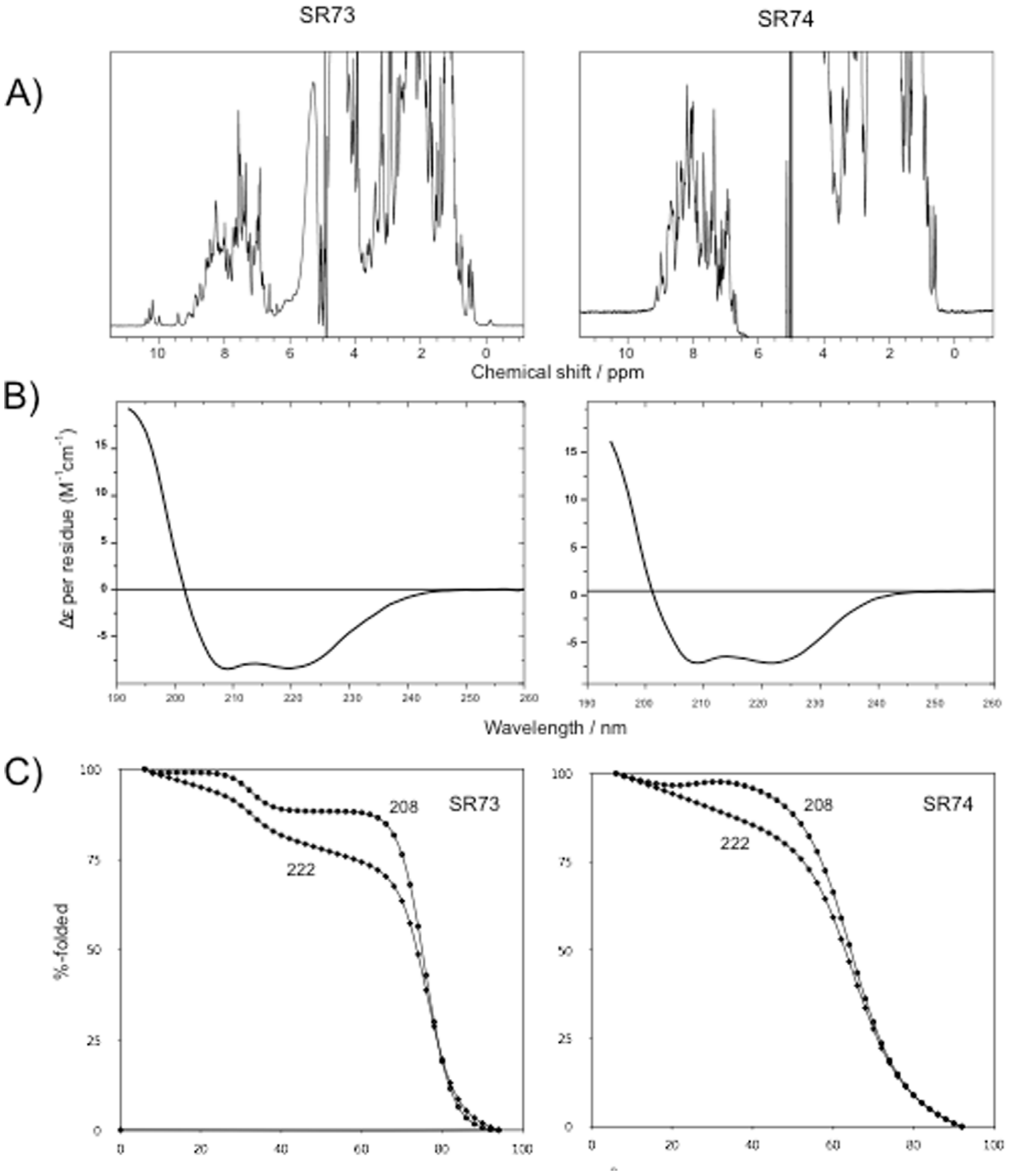
Spectroscopic characterisation of spectrin repeats SR73 and SR74. (A) 1D NMR spectra. (B) CD spectra. (C) Thermal unfolding curves measured by CD at wavelengths of 222 and 208 nm

A selection of predicted spectrin repeats amongst canonical (^NES1^SR73 and ^NES2^SR55) and non-canonical (^NES1^SR74 and ^NES2^SR56) was characterised by circular dichroism (CD) and nuclear magnetic resonance (NMR) spectroscopy. The results of these are summarised in Figure 7 and Figure S4 for a representative established (^NES1^SR73 and ^NES2^SR55) and newly predicted (^NES1^SR74 and ^NES2^SR56) spectrin repeat. Numerical results are summarised in Table 2 for all the characterised SRs. The NMR spectra show excellent dispersion in the high- and low field region indicative of the presence of hydrogen bonds and aliphatic-aromatic contacts, hallmarks of a folded protein. The relatively sharp peaks are indicative of monodisperse proteins and the absence of complex dynamics and aggregation or self-association. The absence of the latter is also confirmed by the measurement of translational diffusion coefficients that agree for all characterised repeats with monomeric species. The CD spectra show a strong, typical α-helical signal with minima at 208 and 222 nm. Fitting the CD spectra to standards of helix, sheet and random coil gives high proportion of helical structure from 60-80% in all of the repeats (Table 2). Thermal denaturation shows high cooperativity of unfolding at high melting temperatures indicative of a well folded and stable domain.

**Table 2:** Summary of the results of the spectroscopic characterisation of the SRs.

construct	% α	%β	% rc	Tm/°C	D/10^−10^ m^2^s^−1^	MW/kD
SR55	65	17	19	72.6 +-1.9	0.988 +- 0.06	16.1
SR56	76	12	12	59.9 +-0.1	1.185 +- 0.03	12.9
SR73	80	20	0	77.7 +-0.1	1.048 +- 0.04	16.3
SR74	75	20	5	68.1 +- 0.1	1.256 +- 0.03	13.1

In case of multiple thermal unfolding events the temperature of the highest transition is listed in the table.

### Molecular Dynamics Simulations of single and double SRs of nesprin

The flexibility of SR proteins was studied to clarify the molecular determinants involved in the elasticity and flexibility of these units. To simplify the comparative analysis and to extrapolate functional mechanical properties we investigated the flexibility of nesprin-1 and nesprin-2 using the most conserved and consecutive SRs among these proteins. As mentioned previously, ^NES1^SR70-SR71 have 78% sequence identity with the corresponding SRs in nesprin-2 (^NES2^SR52-SR53). Therefore these two modules were subjected to Molecular Dynamics (MD) simulations in aqueous solution. For comparison, we also performed MD simulations of the crystal structure of human erythroid β-spectrin HEβ89 (pdb code 1S35) [37], because this structure has been used previously to study the flexibility of double repeats by means of MD simulations [38]. MD simulations were also performed on each single SR forming the double SRs selected.

**Figure 8:**
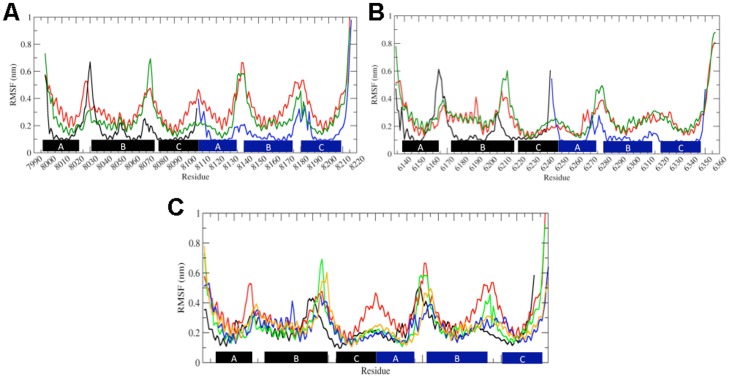
RMSF of the single and double SR units. (A) RMSF of single units (black and blue lines) and replica simulations performed on the double SR unit (red and green lines) of ^NES1^SR70-71. (B) RMSF of single units (black and blue lines) and the replica simulations performed on the double SR unit ^NES2^SR52-53. (C) Comparison of the RMSF of the following double SR units: 1S35 (black line), ^NES1^SR70-71 replicas (red and green lines) and ^NES2^SR52-53 replicas (blue and yellow lines).

The root mean squared fluctuations (RMSFs) were used to highlight differences in terms of flexibility of isolated SRs compared to the same SR in the double repeats (Figure 8A and B). The isolated SRs in solution appeared to be very stable, maintaining all the secondary structure elements of the initial structure. The only flexible regions were the loops AB and BC that show a high mobility mainly due to the interaction with the N- and C-terminal region of the domain (Figure 8A). The double SRs show overall more flexibility. Analysis of correlated motions of the studied systems showed that in all systems the mobility of the AB loop is correlated with the linker region; in particular the loops of ^NES2^SR52-53 seem to have a more independent motion from the linker region when compared to ^NES1^SR70-71 (Figure S5). The observed flexibility did not influence the helical content of the SR structures. During the simulations, the α-helices in the starting X-ray structure (1S35) were stable and only minor secondary structure fluctuations were observed. The two models, ^NES1^SR70-SR71 and ^NES2^SR52-53, show a slight variation of their secondary structure elements during the simulations compared to the crystal structure, although the majority of secondary elements present before the MD simulations remained stable. The major differences between the crystal structure and the SR models (before simulation) were found in the linker region; this does not remain stable as an α-helix, but it is converted to a turn in the ^NES1^SRs70-71 model and to an atypical helix (π-helix) in ^NES2^SR52-53. During the simulations the residues forming the turn in ^NES1^SR70-71 seemed to stabilize and convert into an α-helix, while the residues forming the π-helix in ^NES2^SR52-53 did not show any variation (Figure S6).

### Principal Component Analysis of motion: difference in mobility of nesprin-1 and nesprin-2 SRs?

To obtain information on the motion observed during the simulations, we performed a principal component analysis (PCA) [39] using the trajectories from the MD simulations. In the PCA analysis the motion is decomposed into principal components (PCs) that are associated with an eigenvector and an eigenvalue. The trajectory can be projected onto these eigenvectors to characterize the motion of the system along selected eigenvector directions. In order to characterize and compare the motion of the simulated systems we decided to evaluate the essential space sampled by each system. This evaluation can be performed using the inner product of the eigenvectors; since the eigenvectors are normalized, an inner product with a value of 1 means that eigenvectors are identical and therefore the essential space sampled is identical.

The inner product was extracted by calculating the average square projection of the first 10 eigenvectors of one set onto the first 10 eigenvectors of another set. The inner product between ^NES1^SR70-71 and ^NES2^SR52-53 showed a large overlap (> 0.70) between the first and the second eigenvectors, demonstrating that the motion of double SRs along the first two principal components is very similar. The same analysis was performed to evaluate the overlap of the models *versus* the X-ray structure 1S35 (Figure S7). The essential space analysis between 1S35 and ^NES1^SR70-71 shows a similarity (0.73) only between the second eigenvector (E2) of 1S35 and the first (E1) of ^NES1^SR70-71, whereas ^NES2^SR52-53 shows a large overlap between both the E1s (0.75) and E2s (0.83).

**Figure 9:**
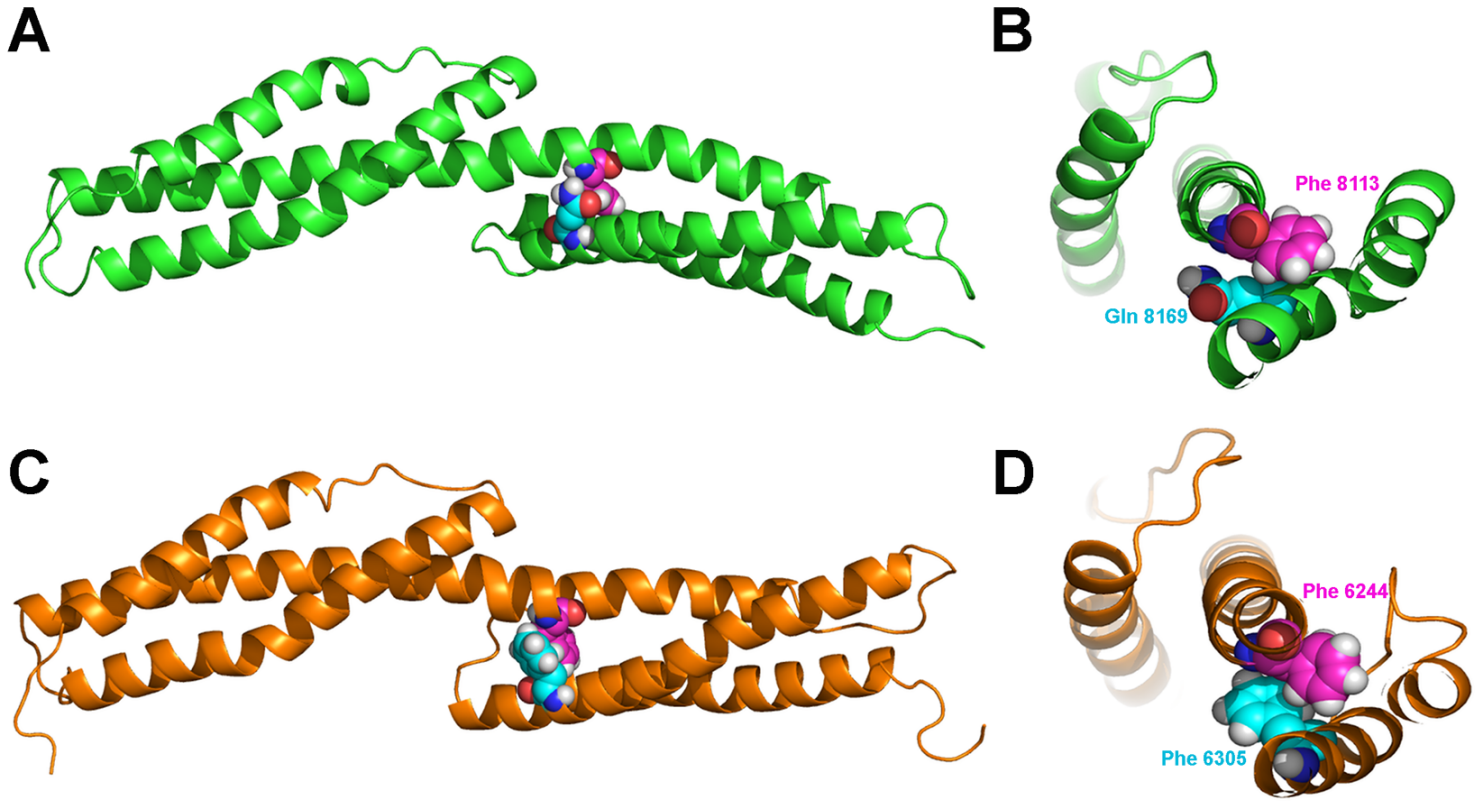
Ribbon representation of ^NES1^SR70-71 (A-B) and ^NES2^SR52-53 (C-D). Close up of the interaction between Phe8113 (magenta spheres) and Gln8169 (cyan spheres) in ^NES1^SR70-71 (B) and between Phe6244 Phe8113 (magenta spheres) and Gln8169 (cyan spheres) in ^NES2^SR52-53 (D).

To visualize the nature of the motion we used porcupine plots displaying the direction of the motion of the C*α* atoms along the first eigenvector (Figure S8). The porcupine plot shows that the flexibility observed during the simulation is mainly due to a bending motion of the two SR units of the double SR with respect to each other. The bending motion appears to be a natural mode in the spectrin protein family, as it has also been observed as natural movement for the α-actinin rod domain [40]. As expected, the observed mobility involved largely the AB and B'C' loops and the linker region. In ^NES1^SR70-71 the motion of both loops is correlated with the linker, while for ^NES2^SR52-53 it correlates only with the loop AB. As mentioned above the sequence identity between ^NES1^SR70-71 and ^NES2^SR52-53 is very high, nevertheless the B'C' loop is one of the regions with a lower identity. A visual inspection of the trajectories revealed that during the simulation there was a strong hydrophobic interaction between ^NES2^Phe6244 (helix A') and ^NES2^Phe6305 (helix B') (Figure 9C-D); this hydrophobic interaction stabilized the two helices, forming a strong interaction in the proximity of the linker and impeding a large degree of flexibility in that region. In comparison, in ^NES1^SR70-71, ^NES1^Phe8113 (helix A') formed a weaker interaction with the ^NES1^Gln8169 (helix B') allowing the B'C' loop to move and to form additional interactions with the linker region participating in the bending motion (Figure 9A-B). The importance of loop sequences and their interaction with the linker region has been shown to be crucial in determining the forced unfolding of the linker region [41]. Furthermore, it has been suggested that the presence of hydrophobic residues in those regions can protect the linker residues from water molecules [41] and make them more resistant to unfolding. The hydrophobic interactions that we observed at the linker region of ^NES2^SR52-53 could explain why its B'C' loop appears less flexible than the B'C' loop of ^NES1^SR70-71.

## Conclusions

It is now well established that nesprin-1 and nesprin-2 are proteins involved in many cellular functions [42], but little information is available on their structure, their binding characteristics and their binding partners. In particular, very little is known about the function of the central rod domain that constitutes the main structural backbone of these proteins. So far the SR units forming the rod domain were considered largely to play the role of spacers between the better-characterized N-terminal CH domains and the C-terminal KASH domain. The information distilled from our large-scale comparative modelling of each SR unit of nesprin-1 and nesprin-2 has been combined with a detailed analysis of the evolutionary conservation and the accessible surface area. The 3D models have afforded a more comprehensive and accurate characterization of the rod domain of these proteins.

One of the most remarkable achievements of this large-scale bioinformatics study is the identification of accurate boundaries of nesprin SRs, that allowed for the design, expression and purification of stable constructs of individual SRs for structural evaluation.

A set of predicted canonical (^NES1^SR73 and ^NES2^SR55) and non-canonical (^NES1^SR74 and ^NES2^SR56) was characterised by CD and NMR spectroscopy, highlighting a stable folded protein as confirmed from temperature denaturation studies, with a high proportion of helical structure from 60 to 80% for both the canonical and non-canonical repeats. These results are strongly indicative of the studied nesprins assuming a typical SR fold.

In addition, new potential binding regions were predicted by our analyses and mapped onto the surface of the SRs. This information can now inform experiments aimed identifying new binding partners and/or defining the interfaces and key residues directly involved in such binding processes.

A more complete analysis of the two short isoforms, nesprin-1α and nesprin-2β, involved in the pathogenesis of EDMD, was also performed. These two isoforms show a very high sequence identity; nevertheless the evolutionary conservation of vertebrate nesprin-1 proteins across this region, is much higher compared to that of nesprin-2. Their similarities in terms of the conserved pbs on the surface led to the identification of potential loci for the interaction with their common partners, emerin and lamin A [8], [10], [11], [12], [13]. A further inspection of the conserved surface, uniquely present in nesprin-1α and not in nesprin-2β, indicates a potential homodimerisation interface [12] for nesprin-1α. EDMD can be caused by the disruption of the interaction between nesprins and the two partners, emerin and lamin A/C, but the molecular determinants of these interactions have been difficult to establish. Our models contribute to identifying these possible molecular determinants and can now be used to guide site-directed mutagenesis to more precisely experimentally define the surfaces involved in these interactions. In addition, these models can now be used to determine the effects of the nesprin mutations identified in EDMD patients on the molecular properties of SRs.

Molecular Dynamics simulation performed on the most conserved and consecutive SR, ^NES1^SR70-71 and ^NES2^SR52-53, showed a preferential bending motion of the SRs with respect to each other. Moreover additional analyses of the simulations have pointed out how a difference in the linker sequence region can influence the flexibility of each SRs

## Methods

### Extraction of single domain SR Structures and Boundary Assignments

Protein Structures containing SRs were extracted by BLAST [43] from the Protein Data Bank (PDB). The selected structures are: human α-actinin 1 (P12814), α-actinin 2 (P35609), α-actinin 3 (Q08043), α-actinin 4 (Q43707), chicken α-actinin 1 (P05094), human α-spectrin 1 (P02549), α-spectrin 2(Q13813), chicken α-spectrin 2 (P07751), human β-spectrin 1 (P11277), β-spectrin 2 (Q01082), chicken β-spectrin 2 (P07751), fruit fly α-spectrin (P13395), human utrophin (P46939), human dystrophin (P11532) and human nesprin-1 (Q8NF91) as reported in Table 1. To be noted that some of the selected structures contain more than a single SR. To extract single SR domain structures from templates with multiple SRs to be used in the modelling procedure, we had to assign the correct boundaries that define the ‘canonical’ SR topology.

The single domain SR templates were extracted by Perl scripts following the SR assignments on their protein sequences by SCOP [44] and SWISSPROT database [45]. The sequences of these single domain SRs, were used with HMMER2.3 [46] to construct a tailored SR seed alignment based on the alignment (PF00435) [20]. Newly defined boundaries were assigned based on SCOP assignment for SR, which divides adjacent SRs into two triple-helix structure units. Structural alignments of these assigned domain structures were carried out with web server MAMMOTH-mult [47].

3DCoffee software [48] has been used to generate the high-quality multiple sequence alignments of nesprins SRs (Figure S1). The sequences of SR templates extracted from SCOP were used to guide the multiple alignments since the structural information extract from the templates can help to improve the quality of the alignments.

### Large-scale Comparative Modelling

Individual SRs in nesprin-1 (74 SRs) and nesprin-2 (56 SRs) were extracted from the alignment of vertebrate nesprins supplied by Simpson and Roberts [19] (their Supplementary Figure 1).

A comparative modelling approach was applied to build 3-D models for the putative nesprin SR sequences. Recognition of SRs in nesprins by Simpson and Roberts [19] based on convergent comparative arguments was adopted in this work. Due to potential boundary assignment errors of the repeats, the flanking residues on both termini of each repeat were included for target-template sequence alignment generation. The extra appending N- and C- terminal residues of target sequences in the alignment were removed before structure modelling.

Cladograms were constructed based on template-target alignments with the program CLUSTALX2 [49] and the web server DrawTree (http://www.phylodiversity.net/rree/drawtree/index.html) (Figure S9-S11).

Here, only SRs with complete structures were included as potential template structures. Structures determined by X-ray and with high solution were preferred to structures solved by other experimental methods like NMR or cryoelectron microscopy (cryo-EM). For each target repeat sequence, the structure closest to it on the cladogram was selected as its optimal template. Alignments of all the nesprins SRs with the selected template(s) are shown in Figure S12A and B. Comparative modelling was then carried out to generate 200 models for each target sequence with MODELLER8v2 [50]. Residues in these models were re-numbered according to their position in the full sequences of human nesprin-1 and -2 by Perl scripts.

### Model Quality Evaluation

Secondary structures of model structures were assigned using DSSP [51]. Helical content of model structures was assigned by a Perl script. The web server Verify3D [29] was used to analyze the geometry of model structures without all-atom contacts and the software ProSA [28] to evaluate model accuracy and statistical significance.

### 3-D Conservation Pattern Analysis

Projection of evolutionary conservation scores of residues calculated from Simpson and Roberts’ alignment [19] (their Supplementary Figure 1) for each SR domain in nesprin-1 or nesprin-2 onto their respective 3-D structure models was performed with the web server ConSurf [30]. The SASA of each residue in the 3-D models was calculated with the program POPS [36]. A Perl script was run to extract residues that are both solvent-exposed and highly conserved (conservation score >7). Non-bonded residues were defined to be neighbours if the 3-D distances between C_α_ atoms of two residues in one model were less than or equal to 10 Å.

Contact i,j  =  0 if {|i-j| ≤ 1};

Contact i,j  =  1 if {|i-j| > 1 & d(i,j) ≤ 10 Å}

Here d(i,j) is the distance between Cα atoms of residues i and j.

Conservation pbs were defined as residues with more than 5 conserved neighbour residues within 10 Å.

### Molecular Dynamics Simulations

Simulations of the single and double SRs were performed with the GROMACS package [52]using the force field ffG53a6 [53]. The molecules were solvated in a box of SPC water [54] and neutralized with Na^+^ ions. The simulated boxes contained ∼ 15000 water molecules for the single SR and ∼ 25000 for the double SRs. Simulations were carried out at a constant temperature of 300 K. The Berendsen algorithm was applied for the temperature and pressure coupling [55]. Prior to the simulations, the potential energy of each system was minimized using a steepest descent and conjugate gradient approaches. 200 ps MD simulations steps with position restraints (using decreasing force constant from 2000 to 500 kJ mol^–1^ nm^–2^) on solute atoms were performed to relax the water molecules. A 2 ns simulation without restrains was performed to equilibrate each system before starting the MD simulations. The particle mesh Ewald method (PME) [56] was used for the calculation of electrostatic contributions to non-bonded interactions (grid spacing of 0.12 nm) with a cut-off of 1.4 nm and a time step of 2 fs. The trajectory length of the single and double SRs was 20 and 50 ns, respectively. The Dynamite server (http://s12ap550.bioch.ox.ac.uk:8078/dynamite_html/index.html) was used to produce further PCA analyses of the MD trajectories. Secondary structure assignment was performed using DSSP [51]. Structure images were produced with Visual Molecular Dynamics (VMD 1.8.5) [57].

### Cloning and protein expression

Multiple cDNAs encoding human nesprin-1 SRs (Ensembl Transcript ID: ENSG00000131018) residues 8416-8552 and 8554-8667, and nesprin-2 SRs (Ensembl Transcript ID: ENSG00000054654) residues 6529-6664 and 6665-6771 were amplified by PCR using full-length ΔTM (lacking the transmembrane region) nesprin-1α and -2β cDNA constructs. Each PCR product was digested with *EcoRI* and *SalI* and cloned into pGEX-4T-3 (Pharmacia Biotech Inc; N-terminal tagged glutathione S- transferase; GST). All constructs were verified by DNA sequencing (source bioscience). The constructs are expressed as a fusion protein containing GST N-terminally attached. For protein expression, constructs were transformed into *Escherichia coli* BL21* cells (Invitrogen). After growth to a *D*_600_ of ∼0.5 at 37°C, the temperature was lowered to 18°C and expression was induced with 0.1 mM IPTG overnight. The cell pellet was resuspended in 30 mL of cold PBS [50 mM NaPi and 150 mM NaCl (pH 7.4)]. Cells were opened by French press followed by centrifugation at 18000 rpm for 40 min in a Sorvall SS34 rotor. The supernatant was loaded on to a gravity flow column filled with 4 ml glutathione-Sepharose 4B beads (GE Healthcare) equilibrated with PBS. The column was washed with 30 ml of PBS. To remove the GST tag, 20 U/ml of Thrombin protease were loaded on to the column at room temperature for 16 h. Where required, the protein was polished on a preparative gel-filtration column (HiLoad 26/60 Sephadex 75; GE Healthcare). The flowthrough and wash fractions were checked by SDS/PAGE, pooled and loaded in a gravity flow PD10 desalting column, (GE Healthcare) against measurement buffer [20 mM sodium phosphate, pH 7.5, 50 mM NaCl, 2 mM DTT (dithiothreitol) and 0.02% sodium azide]. The purity of protein samples was checked on SDS/PAGE 4–12% gradient gels (NuPAGE®, Invitrogen).

### CD and NMR Spectroscopy

CD spectra were recorded on a Photophysics Chirascan spectrometer (Leatherhead, UK) using 1.0 and 0.5 mm spectrasil rectangular quartz cuvettes (Hellma, UK), 1 nm spectral bandwidth, 1 nm stepsize and 1.5 s instrument time per point. Secondary structure content was estimated using in house software tools as described previously [58].

1D NMR spectra were recorded on a 700 MHz Bruker Avance spectrometer using watergate for water suppression [59] as described previously [60]. Diffusion coefficients were measured on a Bruker Avance 500 MHz spectrometer using a convection compensated double stimulated echo experiment [61]. A total of twenty gradient strengths from 4-46 G/cm with a step size of 2 G/cm were used for a duration of 1.75 ms and a diffusion time of 120 ms. All experiments were measured at a temperature of 298 K and in a buffer of 20 mM sodium phosphate pH 7.0, 50 mM sodium chloride, 2 mM dithiothreitol and 0.02% sodium azide with protein concentrations ranging from 40-200 µM.

## Supporting Information

Figure S1 **nesprin-1 (A) and nesprin-2 (B) SRs multiple sequence alignment obtained with 3DCoffee.** Alignment quality based on Blosum 62 scores.
(PDF)

Figure S2 **nesprin-1 (A) and nesprin-2 (B) boundaries.** The first residue for each SR has been indicated. The residue highlighted in gray belong to the linker region at the interface of two SRs.
(PDF)

Figure S3 **Percentage of conserved residue for each SR unit (A-B).** Percentage of buried and exposed residues among the total conserved residue for each SRs (C-D).
(PDF)

Figure S4 **Spectroscopic characterisation of spectrin repeats SR55 and SR56.** (A) 1D NMR spectra. (B) CD spectra.(C) Thermal unfolding curves measured by CD at wavelengths of 222 and 208 nm. (D) Purified SR proteins studied.
(PDF)

Figure S5 **Correlated motions of C*α* residues belonging to 1S35 (A) NES1 SR70-71 and NES2SR52-53 (C). Motions** with >80% correlation are indicated by lines connecting the involved residues.
(PDF)

Figure S6 **Time evolution of the secondary structure elements during MD simulations.** Positions of secondary structure elements α-helices A B C and A’ B’ and C’ 5 are indicated on the y-axis and the simulation time in nanoseconds is indicated on the x-axis. Colour indicate secondary structure elements at a given time point as determined by DSSP classification.
(PDF)

Figure S7 **Inner product of the first 10 eigenvector between 1S35 - NES2SR52-53 (A), 1S35 - NES1 SR70-71 (B) and NES2SR52-53-NES1SR70-71(C).**
(PDF)

Figure S8 **Porcupine plots of the motions corresponding to the first eigenvector of the simulations of NES1SR70-71 (A) and NES2SR52-53 (B).** Each Cα atom has a cone attached pointing in the direction of motion described by the eigenvector corresponding to that atom.
(PDF)

Figure S9 **Cladogram of Nesprin-1 SRs assigned by SWISSPROT based on the alignment templates- targets performed with CLUSTALX2 program and web server DrawTree.** The template used for each group of SRs is highlighted on the side.
(PDF)

Figure S10 **Cladogram of Nesprin-1 SRs not assigned by SWISSPROT based on the alignment templates-targets performed with CLUSTALX2 program and web server DrawTree.** The template used for each group of SRs is highlighted on the side.
(PDF)

Figure S11 **Cladogram of Nesprin-2 SRs based on the alignment templates-targets performed with CLUSTALX2 program and web server DrawTree.** The template used for each group of SRs is highlighted on the side.
(PDF)

Figure S12 **Alignment used to build the models of nesprin-1 (A) and nesprin-2 (B) SRs**
(PDF)

Table S1 **nesprin-1 SRs Z-Score from ProSA [28] In bolded are higlighed the SR assigned also by Pfam.**
(PDF)

Table S2 **nesprin-2 SRs Z-Score from ProSA [28] In bolded are higlighed the SR assigned also by Pfam.**
(PDF)

Table S3 **Nesprin1α hot spots.**
(PDF)

Table S4 **Nesprin2β hot spots.**
(PDF)
