# Supplementary figures and images for: Large-Scale Modelling of the Divergent Spectrin Repeats in Nesprins: Giant Modular Proteins
Source: PLoS One. 2013 May 6;8(5):e63633. doi: 10.1371/journal.pone.0063633 (PMC3646009; doi:10.1371/journal.pone.0063633)

A

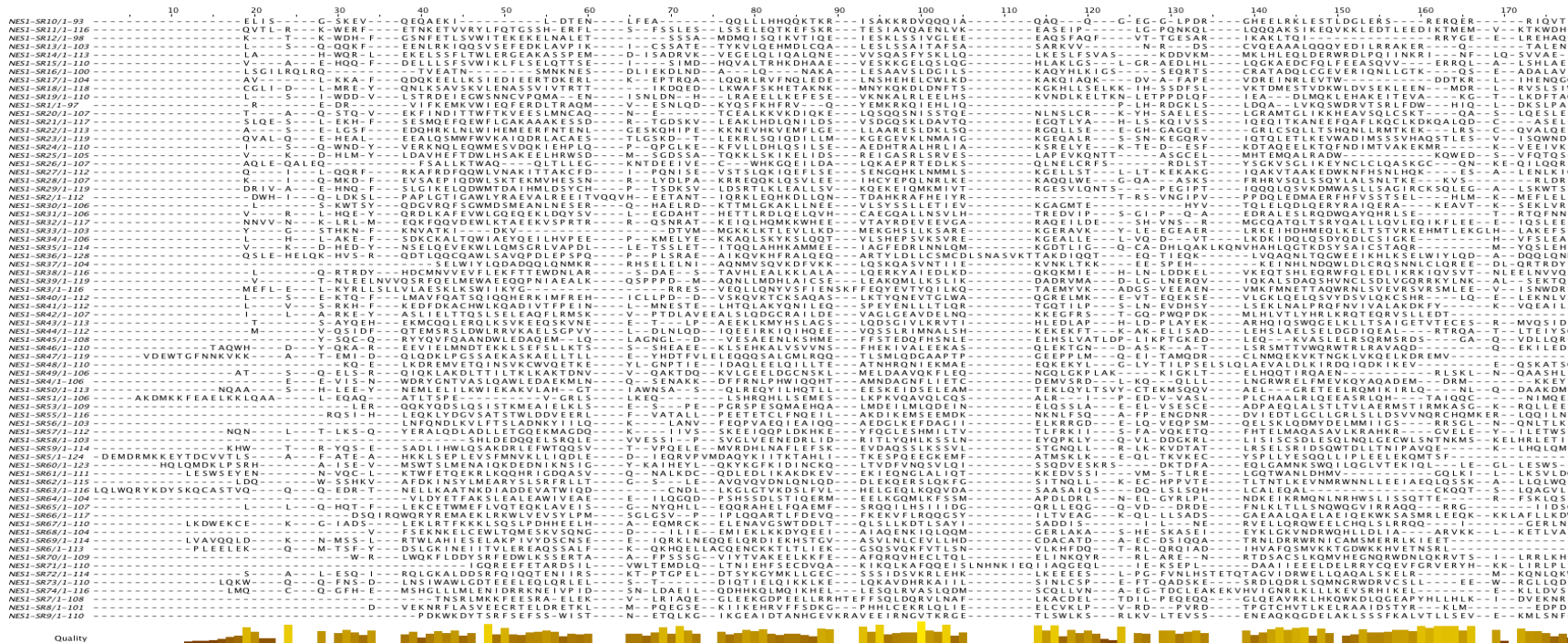

B

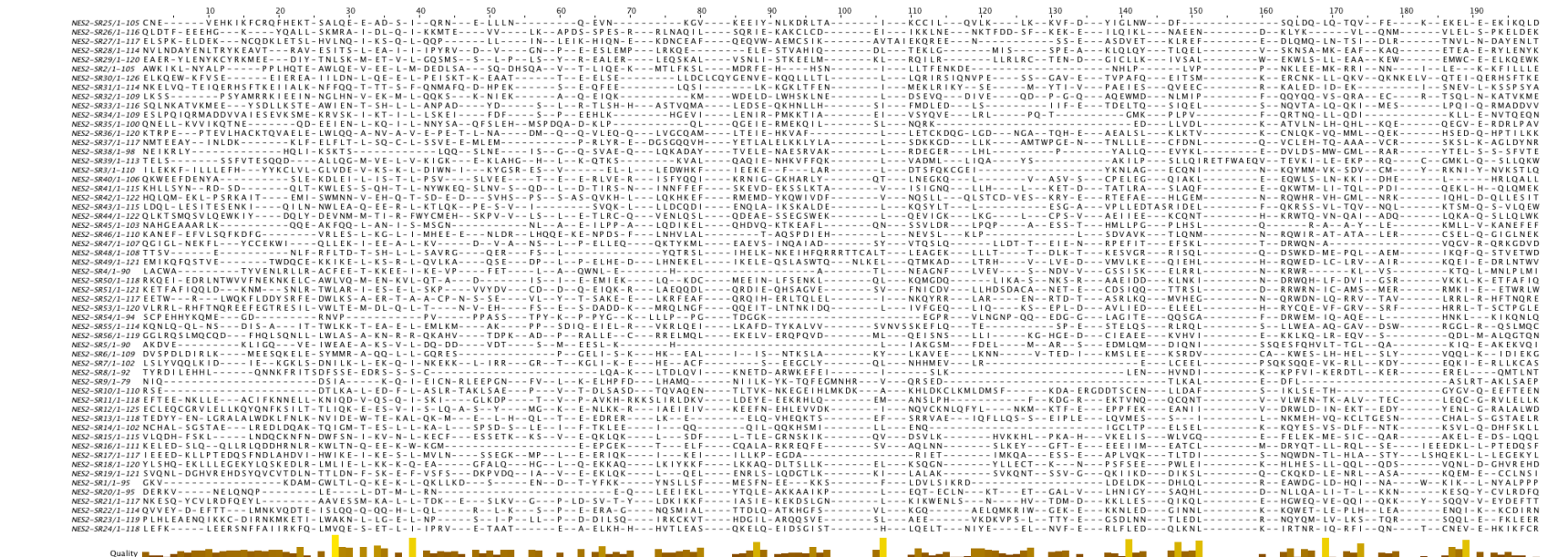

Supplement: Figure S1 — nesprin-1 (A) and nesprin-2 (B) SRs multiple sequence alignment obtained with 3DCoffee. Alignment quality based on Blosum 62 scores. (PDF) [file pone.0063633.s001.pdf]
