# Supplementary material for: Large-Scale Modelling of the Divergent Spectrin Repeats in Nesprins: Giant Modular Proteins
Source: PLoS One. 2013 May 6;8(5):e63633. doi: 10.1371/journal.pone.0063633 (PMC3646009; doi:10.1371/journal.pone.0063633)
Supplement: Figure S2 — nesprin-1 (A) and nesprin-2 (B) boundaries. The first residue for each SR has been indicated. The residue highlighted in gray belong to the linker region at the interface of two SRs. (PDF) [file pone.0063633.s002.pdf]

Figure S2A: nesprin-1 boundaries

A

|CH domain
MATSRGASRCPRDIANVMQRLQDEQEIVQKRTFTKWINSHLAKRKPPMVVDDLFEDMKDGVKLLALLEVLSGQKLPCEQGRRMKRIHAVANIGTALKFLEGRKIKLVNINSTDIADGRPS 120

| |CH domain
IVLGLMWTIILYFQIEELTSNLPQLQSLSSSASSVDSIVSSETPSPPSKRKVTTKIQGNAKKALLKWVQYTAGKQTGIEVKDFGKSWRSGVAFHSVIHAIRPELVDLETVKGRSNRENLE 240

| |R314 SR1
DAFTIAETELGIPRLLDPEDVDVDKPDEKSIMTYVAQFLKHYPDIHNASTDGQEDDEILPGFPSFANSVQNFKREDRVIFKEMKVWIEQFERDLTRAQMVESNLQDKYQSFKHFRVQYEM 360

|D398 SR2
KRKQIEHLIQPLHRDGKLSLDQALVKQSWDRVTSRLFDWHIQLDKSLPAPLGTIGAWLYRAEVALREEITVQQVHEETANTIQRKLEQHKDLLQNTDAHKRAFHEIYRTRSVNGIPVPPD 480

|M503 SR3
QLEDMAERFHFVSSTSELHLMKMEFLELKYRLLSLLVLAESKLKSWIIKYGRRESVEQLLQNYVSFIENSKFFEQYEVTYQILKQTAEMYVKADGSVEEAENVMKFMNETTAQWRNLSVE 600

|E610 SR4 |D704 SR5
VRSVRSMLEEVISNWDRYGNTVASLQAWLEDAEKMLNQSENAKKDFFRNLPHWIQQHTAMNDAGNFLIETCDEMVSRDLKQQLLLLNGRWRELFMEVKQYAQADEMDRMKKEYTDCVVTL 720

|P816 SR6
SAFATEAHKKLSEPLEVSFMNVKLLIQDLEDIEQRVPVMDAQYKIITKTAHLITKESPQEEGKEMFATMSKLKEQLTKVKECYSPLLYESQQLLIPLEELEKQMTSFYDSLGKINEIITV 840

|T924 SR7
LEREAQSSALFKQKHQELLACQENCKKTLTLIEKGSQSVQKFVTLSNVLKHFDQTRLQRQIADIHVAFQSMVKKTGDWKKHVETNSRLMKKFEESRAELEKVLRIAQEGLEEKGDPEELL 960

|D1025 SR8
RRHTEFFSQLDQRVLNAFLKACDELTDILPEQEQQGLQEAVRKLHKQWKDLQGEAPYHLLHLKIDVEKNRFLASVEECRTELDRETKLMPQEGSEKIIKEHRVFFSDKGPHHLCEKRLQL 1080

|P1123 SR9
IEELCVKLPVRDPVRDTPGTCHVTLKELRAAIDSTYRKLMEDPDKWKDYTSRFSEFSSWISTNETQLKGIKGEAIDTANHGEVKRAVEEIRNGVTKRGETLSWLKSRLKVLTEVSSENEA 1200

| |E1247 SR10
QKQGDELAKLSSSFKALVTLLSEVEKMLSNFGDCVQYKEIVKNSLEELISGSKEVQEQAEKILDTENLFEAQQLLLHHQQKTKRISAKKRDVQQQIAQAQQGEGGLPDRGHEELRKLEST 1320

Figure S2A: nesprin-1 boundaries

|Q1336 SR11
LDGLERSRERQERRIQVTLRKWERFETNKETVVRYLFQTGSSHERFLSFSSLESLSSELEQTKEFSKRTESIAVQAENLVKEASEIPLGPQNKQLLQQQAKSIKEQVKKLEDTLEEDIKT 1440

|K1445 SR12 |L1551 SR13
MEMVKTKWDHFGSNFETLSVWITEKEKELNALETSSSAMDMQISQIKVTIQEIESKLSSIVGLEEEAQSFAQFVTTGESARIKAKLTQIRRYGEELREHAQCLEGTILGHLSQQQKFEEN 1560

|L1654 SR14
LRKIQQSVSEFEDKLAVPIKICSSATETYKVLQEHMDLCQALESLSSAITAFSASARKVVNRDSCVQEAAALQQQYEDILRRAKERQTALENLLAHWQRLEKELSSFLTWLERGEAKASS 1680

|V1764 SR15
PEMDISADRVKVEGELQLIQALQNEVVSQASFYSKLLQLKESLFSVASKDDVKMMKLHLEQLDERWRDLPQIINKRINFLQSVVAEHQQFDELLLSFSVWIKLFLSELQTTSEISIMDHQ 1800

| |L1880 SR16
VALTRHKDHAAEVESKKGELQSLQGHLAKLGSLGRAEDLHLLQGKAEDCFQLFEEASQVVERRQLALSHLAEFLQSHASLSGILRQLRQTVEATNSMNKNESDLIEKDLNDALQNAKALE 1920

|A1977 SR17
SAAVSLDGILSKAQYHLKIGSSEQRTSCRATADQLCGEVERIQNLLGTKQSEADALAVLKKAFQDQKEELLKSIEDIEERTDKERLKEPTRQALQQRLRVFNQLEDELNSHEHELCWLKD 2040

||C2082 SR18
KAKQIAQKDVAFAPEVDREINRLEVTWDDTKRLIHENQGQCCGLIDLMREYQNLKSAVSKVLENASSVIVTRTTIKDQEDLKWAFSKHETAKNKMNYKQKDLDNFTSKGKHLLSELKKIH 2160

|L2196 SR19
SSDFSLVKTDMESTVDKWLDVSEKLEENMDRLRVSLSIWDDVLSTRDEIEGWSNNCVPQMAENISNLDNHLRAEELLKEFESEVKNKALRLEELHSKVNDLKELTKNLETPPDLQFIEAD 2280

|T2304 SR20
LMQKLEHAKEITEVAKGTLKDFTAQSTQVEKFINDITTWFTKVEESLMNCAQNETCEALKKVKDIQKELQSQQSNISSTQENLNSLCRKYHSAELESLGRAMTGLIKKHEAVSQLCSKTQ 2400

|S2402 SR21 |A2514 SR22
ASLQESLEKHFSESMQEFQEWFLGAKAAAKESSDRTGDSKVLEAKLHDLQNILDSVSDGQSKLDAVTQEGQTLYAHLSKQIVSSIQEQITKANEEFQAFLKQCLKDKQALQDCASELGSF 2520

|Q2620 SR23
EDQHRKLNLWIHEMEERFNTENLGESKQHIPEKKNEVHKVEMFLGELLAARESLDKLSQRGQLLSEEGHGAGQEGRLCSQLLTSHQNLLRMTKEKLRSCQVALQEHEALEEALQSMWFWV 2640

Figure S2A: nesprin-1 boundaries

|I2732 SR24

KAIQDRLACAESTLGSKDTLEKRLSQIQDILLMKGEGEVKLNMAIGKGEQALRSSNKEGQRVIQTQLETLKEVWADIMSSSVHAQSTLESVISQWNDYVERKNQLEQWMESVDQKIEHPL 2760

|V2839 SR25

QPQPGLKEKFVLLDHLQSILSEAEDHTRALHRLIAKSRELYEKTEDESFKDTAQEELKTQFNDIMTVAKEKMRKVEEIVKDHLMYLDAVHEFTDWLHSAKEELHRWSDMSGDSSATQKKL 2880

| |A2963 SR26

SKIKELIDSREIGASRLSRVESLAPEVKQNTTASGCELMHTEMQALRADWKQWEDSVFQTQSCLENLVSQMALSEQEFSGQVAQLEQALEQFSALLKTWAQQLTLLEGKNTDEEIVECWH 3000

|Q3063 SR27

KGQEILDALQKAEPRTEDLKSQLNELCRFSRDLSTYSGKVSGLIKEYNCLCLQASKGCQNKEQILQQRFRKAFRDFQQWLVNAKITTAKCFDIPQNISEVSTSLQKIQEFLSESENGQHK 3120

|K3172 SR28

LNMMLSKGELLSTLLTKEKAKGIQAKVTAAKEDWKNFHSNLHQKESALENLKIQMKDFEVSAEPIQDWLSKTEKMVHESSNRLYDLPAKRREQQKLQSVLEEIHCYEPQLNRLKEKAQQL 3240

|D3276 SR29

WEGQAASKSFRHRVSQLSSQYLALSNLTKEKVSRLDRIVAEHNQFSLGIKELQDWMTDAIHMLDSYCHPTSDKSVLDSRTLKLEALLSVKQEKEIQMKMIVTRGESVLQNTSPEGIPTIQ 3360

|L3388 SR30

QQLQSVKDMWASLLSAGIRCKSQLEGALSKWTSYQDGVRQFSGWMDSMEANLNESERQHAELRDKTTMLGKAKLLNEEVLSYSSLLETIEVKGAGMTEHYVTQLELQDLQERYRAIQERA 3480

|V3491 SR31 |N3594 SR32

KEAVTKSEKLVRLHQEYQRDLKAFEVWLGQEQEKLDQYSVLEGDAHTHETTLRDLQELQVHCAEGQALLNSVLHTREDVIPSGIPQAEDRALESLRQDWQAYQHRLSETRTQFNNVVNKL 3600

|

RLMEQKFQQVDEWLKTAEEKVSPRTRRQSNRATKEIQLHQMKKWHEEVTAYRDEVEEVGARAQEILDESHVNSRMGCQATQLTSRYQALLLQVLEQIKFLEEEIQSLEESESSLSSYSDW 3720

|Y721 SR33 |L3815 SR34

YGSTHKNFKNVATKIDKVDTVMMGKKLKTLEVLLKDMEKGHSLLKSAREKGERAVKYLEEGEAERLRKEIHDHMEQLKELTSTVRKEHMTLEKGLHLAKEFSDKCKALTQWIAEYQEILH 3840

|V3921 SR35

VPEEPKMELYEKKAQLSKYKSLQQTVLSHEPSVKSVREKGEALLELVQDVTLKDKIDQLQSDYQDLCSIGKEHVFSLEAKVKDHEDYNSELQEVEKWLLQMSGRLVAPDLLETSSLETIT 3960

|Q4029 SR36

QQLAHHKAMMEEIAGFEDRLNNLQMKGDTLIGQCADHLQAKLKQNVHAHLQGTKDSYSAICSTAQRMYQSLEHELQKHVSRQDTLQQCQAWLSAVQPDLEPSPQPPLSRAEAIKQVKHFR 4080

Figure S2A: nesprin-1 boundaries

|S4140 SR37
ALQEQARTYLDLLCSMCDLSNASVKTTAKDIQQTEQTIEQKLVQAQNLTQGWEEIKHLKSELWIYLQDADQQLQNMKRRHSELELNIAQNMVSQVKDFVKKLQSKQASVNTIIEKVNKLT 4200

|L4236 SR38
KKEESPEHKEINHLNDQWLDLCRQSNNLCLQREEDLQRTRDYHDCMNVVEVFLEKFTTEWDNLARSDAESTAVHLEALKKLALALQERKYAIEDLKDQKQKMIEHLNLDDKELVKEQTSH 4320

|K4340 SR39
LEQRWFQLEDLIKRKIQVSVTNLEELNVVQSRFQELMEWAEEQQPNIAEALKQSPPPDMAQNLLMDHLAICSELEAKQMLLKSLIKDADRVMADLGLNERQVIQKALSDAQSHVNCLSDL 4440

|L4452 SR40
VGQRRKYLNKALSEKTQFLMAVFQATSQIQQHERKIMFREHICLLPDDVSKQVKTCKSAQASLKTYQNEVTGLWAQGRELMKEVTEQEKSEVLGKLQELQSVYDSVLQKCSHRLQELEKN 4560

|L4561 SR41 |I4670 SR42
LVSRKHFKEDFDKACHWLKQADIVTFPEINLMNESSELHTQLAKYQNILEQSPEYENLLLTLQRTGQTILPSLNEVDHSYLSEKLNALPRQFNVIVALAKDKFYKVQEAILARKEYASLI 4680

|T4777 SR43
ELTTQSLSELEAQFLRMSKVPTDLAVEEALSLQDGCRAILDEVAGLGEAVDELNQKKEGFRSTGQPWQPDKMLHLVTLYHRLKRQTEQRVSLLEDTTSAYQEHEKMCQQLERQLKSVKEE 4800

|M4883 SR44
QSKVNEETLPAEEKLKMYHSLAGSLQDSGIVLKRVTIHLEDLAPHLDPLAYEKARHQIQSWQGELKLLTSAIGETVTECESRMVQSIDFQTEMSRSLDWLRRVKAELSGPVYLDLNLQDI 4920

|Y4992 SR45
QEEIRKIQIHQEEVQSSLRIMNALSHKEKEKFTKAKELISADLEHSLAELSELDGDIQEALRTRQATLTEIYSQCQRYYQVFQAANDWLEDAQELLQLAGNGLDVESAEENLKSHMEFFS 5040

|T5100 SR46
TEDQFHSNLEELHSLVATLDPLIKPTGKEDLEQKVASLELRSQRMSRDSGAQVDLLQRCTAQWHDYQKAREEVIELMNDTEKKLSEFSLLKTSSSHEAEEKLSEHKALVSVVNSFHEKIV 5160

|V5210 SR47
ALEEKASQLEKTGNDASKATLSRSMTTVWQRWTRLRAVAQDQEKILEDAVDEWTGFNNKVKKATEMIDQLQDKLPGSSAEKASKAELLTLLEYHDTFVLELEQQQSALGMLRQQTLSMLQ 5280

|K5319 SR48
DGAAPTPGEEPPLMQEITAMQDRCLNMQEKVKTNGKLVKQELKDREMVETQINSVKCWVQETKEYLGNPTIEIDAQLEELQILLTEATNHRQNIEKMAEEQKEKYLGLYTILPSELSLQL 5400

|A5425 SR49
AEVALDLKIRDQIQDKIKEVEQSKATSQELSRQIQKLAKDLTTILTKLKAKTDNVVQAKTDQKVLGEELDGCNSKLMELDAAVQKFLEQNGQLGKPLAKKIGKLTELHQQTIRQAENRLS 5520

Figure S2A: nesprin-1 boundaries

|N5523 SR50 |A5631 SR51

KLNQAASHLEEYNEMLELILKWIEKAKVLAHGTIAWNSASQLREQYILHQTLLEESKEIDSELEAMTEKLQYLTSVYCTEKMSQQVAELGRETEELRQMIKIRLQNLQDAAKDMKKFEAE 5640

|

LKKLQAALEQAQATLTSPEVGRLSLKEQLSHRQHLLSEMESLKPKVQAVQLCQSALRIPEDVVASLPLCHAALRLQEEASRLQHTAIQQCNIMQEAVVQYEQYEQEMKHLQQLIEGAHRE 5760

IEDKPVATSNIQELQAQISRHEELAQKIKGYQEQIASLNSKCKMLTMKAKHATMLLTVTEVEGLAEGTEDLDGELLPTPSAHPSVVMMTAGRCHTLLSPVTEESGEEGTNSEISSPPACR 5880

|L5962 SR53

SPSPVANTDASVNQDIAYYQALSAERLQTDAAKIHPSTSASQEFYEPGLEPSATAKLGDLQRSWETLKNVISEKQRTLYEALERQQKYQDSLQSISTKMEAIELKLSESPEPGRSPESQM 6000

|

AEHQALMDEILMLQDEINELQSSLAEELVSESCEADPAEQLALQSTLTVLAERMSTIRMKASGKRQLLEEKLNDQLEEQRQEQALQRYRCEADELDSWLLSTKATLDTALSPPKEPMDME 6120

AQLMDCQNMLVEIEQKVVALSELSVHNENLLLEGKAHTKDEAEQLAGKLRRLKGSLLELQRALHDKQLNMQGTAQEKEESDVDLTATQSPGVQEWLAQARTTWTQQRQSSLQQQKELEQE 6240

LAEQKSLLRSVASRGEEILIQHSAAETSGDAGEKPDVLSQELGMEGEKSSAEDQMRMKWESLHQEFSTKQKLLQNVLEQEQEQVLYSRPNRLLSGVPLYKGDVPTQDKSAVTSLLDGLNQ 6360

|K6374 SR55

AFEEVSSQSGGAKRQSIHLEQKLYDGVSATSTWLDDVEERLFVATALLPEETETCLFNQEILAKDIKEMSEEMDKNKNLFSQAFPENGDNRDVIEDTLGCLLGRLSLLDSVVNQRCHQMK 6480

|L6486 SR56 |N6582 SR57

ERLQQILNFQNDLKVLFTSLADNKYIILQKLANVFEQPVAEQIEAIQQAEDGLKEFDAGIIELKRRGDKLQVEQPSMQELSKLQDMYDELMMIIGSRRSGLNQNLTLKSQYERALQDLAD 6600

|S6692 SR58

LLETGQEKMAGDQKIIVSSKEEIQQLLDKHKEYFQGLESHMILTETLFRKIISFAVQKETQFHTELMAQASAVLKRAHKRGVELEYILETWSHLDEDQQELSRQLEVVESSIPSVGLVEE 6720

|K6796 SR59

NEDRLIDRITLYQHLKSSLNEYQPKLYQVLDDGKRLLISISCSDLESQLNQLGECWLSNTNKMSKELHRLETILKHWTRYQSESADLIHWLQSAKDRLEFWTQQSVTVPQELEMVRDHLN 6840

|H6903 SR60

AFLEFSKEVDAQSSLKSSVLSTGNQLLRLKKVDTATLRSELSRIDSQWTDLLTNIPAVQEKLHQLQMDKLPSRHAISEVMSWISLMENVIQKDEDNIKNSIGYKAIHEYLQKYKGFKIDI 6960

Figure S2A: nesprin-1 boundaries

|L7021 SR61
NCKQLTVDFVNQSVLQISSQDVESKRSDKTDFAEQLGAMNKSWQILQGLVTEKIQLLEGLLESWSEYENNVQCLKTWFETQEKRLKQQHRIGDQASVQNALKDCQDLEDLIKAKEKEVEK 7080

|L7129 SR62
IEQNGLALIQNKKEDVSSIVMSTLRELGQTWANLDHMVGQLKILLKSVLDQWSSHKVAFDKINSYLMEARYSLSRFRLLTGSLEAVQVQVDNLQNLQDDLEKQERSLQKFGSITNQLLKE 7200

|L7238 SR63
CHPPVTETLTNTLKEVNMRWNNLLEEIAEQLQSSKALLQLWQRYKDYSKQCASTVQQQEDRTNELLKAATNKDIADDEVATWIQDCNDLLKGLGTVKDSLFFLHELGEQLKQQVDASAAS 7320

|V7351 SR64
AIQSDQLSLSQHLCALEQALCKQQTSLQAGVLDYETFAKSLEALEAWIVEAEEILQGQDPSHSSDLSTIQERMEELKGQMLKFSSMAPDLDRLNELGYRLPLNDKEIKRMQNLNRHWSLI 7440

|L7455 SR65 |D7559
SR66
SSQTTERFSKLQSFLLQHQTFLEKCETWMEFLVQTEQKLAVEISGNYQHLLEQQRAHELFQAEMFSRQQILHSIIIDGQRLLEQGQVDDRDEFNLKLTLLSNQWQGVIRRAQQRRGIIDS 7560

|L7672 SR67
QIRQWQRYREMAEKLRKWLVEVSYLPMSGLGSVPIPLQQARTLFDEVQFKEKVFLRQQGSYILTVEAGKQLLLSADSGAEAALQAELAEIQEKWKSASMRLEEQKKKLAFLLKDWEKCEK 7680

|V7784 SR68
GIADSLEKLRTFKKKLSQSLPDHHEELHAEQMRCKELENAVGSWTDDLTQLSLLKDTLSAYISADDISILNERVELLQRQWEELCHQLSLRRQQIGERLNEWAVFSEKNKELCEWLTQME 7800

|7884 SR69
SKVSQNGDILIEEMIEKLKKDYQEEIAIAQENKIQLQQMGERLAKASHESKASEIEYKLGKVNDRWQHLLDLIAARVKKLKETLVAVQQLDKNMSSLRTWLAHIESELAKPIVYDSCNSE 7920

|W7998 SR70
EIQRKLNEQQELQRDIEKHSTGVASVLNLCEVLLHDCDACATDAECDSIQQATRNLDRRWRNICAMSMERRLKIEETWRLWQKFLDDYSRFEDWLKSSERTAAFPSSSGVIYTVAKEELK 8040

|I8107 SR71
KFEAFQRQVHECLTQLELINKQYRRLARENRTDSACSLKQMVHEGNQRWDNLQKRVTSILRRLKHFIGQREEFETARDSILVWLTEMDLQLTNIEHFSECDVQAKIKQLKAFQQEISLNH 8160

|
NKIEQIIAQGEQLIEKSEPLDAAIIEEELDELRRYCQEVFGRVERYHKKLIRLPLPDDEHDLSDRELELEDSAALSDLHWHDRSADSLLSPQPSSNLSLSLAQPLRSERSGRDTPASVDS 8280

Figure S2A: nesprin-1 boundaries

```
                                                                      |S8329 SR72
IPLEWDHDYDLSRDLESAMSRALPSEDEEGQDDKDFYLRGAVGLSGDHSALESQIRQLGKALDDSRFQIQQTENIIRSKTPTGPELDTSYKGYMKLLGECSSSIDSVKRLEHKLKEEEES  8400

                                 |L8439 SR73
LPGFVNLHSTETQTAGVIDRWELLQAQALSKELRMKQNLQKWQQFNSDLNSIWAWLGDTEEELEQLQRLELSTDIQTIELQIKKLKELQKAVDHRKAIILSINLCSPEFTQADSKESRDL  8520

                                        |L8549 SR74
QDRLSQMNGRWDRVCSLLEEWRGLLQDALMQCQGFHEMSHGLLLMLENIDRRKNEIVPIDSNLDAEILQDHHKQLMQIKHELLESQLRVASLQDMSCQLLVNAEGTDCLEAKEKVHVIGN  8640

                                     |                                                                                                   |KASH domain
RLKLLLKEVSRHIKELEKLLDVSSSQQDLSSWSSADELDTSGSVSPTSGRSTPNRQKTPRGKCSLSQPGPSVSSPHSRSTKGGSDSSLSEPGPGRSGRGFLFRVLRAALPLQLLLLLLLIG  8760

LACLVPMSEEDYSCALSNNFARSFHPMLRYTNGPPPL  8797
```

Figure S2B: nesprin-2 boundaries

```
                               |CH domain
MASSPELPTEDEQGSWGIDDLHISLQAEQEDTQKKAFTCWINSQLARHTSPSVISDLFTDIKKGHVLLDLLEVLSGQQLPRDKGSNTFQCRINIEHALTFLRNRSIKLINIHVTDIIDGN  120

                     |                                                |CH domain
PSIILGLIWTIILHFHIEKLAQTLSCNYNQPSLDDVSVVDSSPASSPPAKKCSKVQARWQMSARKALLLWAQEQCATYESVNVTDFKSSWRNGMAFLAIIHALRPDLIDMKSVKHRSNKD  240

                                        |              |G297 SR1
NLREAFRIAEQELKIPRLLEPEDVDVVDPDEKSIMTYVAQFLQYSKDAPGTGEEAQGKVKDAMGWLTLQKEKLQKLLKDSENDTYFKKYNSLLSFMESFNEEKKSFLDVLSIKRDLDELD  360

                  |A379 SR2                                                                                    |I473 SR3
KDHLQLREAWDGLDHQINAWKIKLNYALPPPLHQTEAWLQEVEELMDEDLSASQDHSQAVTLIQEKMTLFKSLMDRFEHHSNILLTFENKDENHLPLVPPNKLEEMKRRINNILEKKFIL  480

                                                                                               |N576 SR4
LLEFHYYKCLVLGLVDEVKSKLDIWNIKYGSRESVELLLEDWHKFIEEKEFLARLDTSFQKCGEIYKNLAGECQNINKQYMMVKSDVCMYRKNIYNVKSTLQKVLACWATYVENLRLLRA  600

                                                                               |
CFEETKKEEIKEVPFETLAQWNLEHATLNEAGNFLVEVSNDVVGSSISKELRRLNKRWRKLVSKTQLEMNLPLMIKKQDQPTFDNSGNILSKEEKATVEFSTDMSVELPENYNQNIKAGE  720

              |V735 SR5                                                                                    |        |D839 SR6
KHEKENEEFTGQLKVAKDVEKLIGQVEIWEAEAKSVLDQDDVDTSMEESLKHLIAKGSMFDELMARSEDMLQMDIQNISSQESFQHVLTTGLQAKIQEAKEKVQINVVKLIAALKNLTDV  840

                                                                                            |L933 SR7
SPDLDIRLKMEESQKELESYMMRAQQLLGQRESPGELISKHKEALIISNTKSLAKYLKAVEELKNNVTEDIKMSLEEKSRDVCAKWESLHHELSLYVQQLKIDIEKGKLSDNILKLEKQI  960

                                                                          |
NKEKKLIRRGRTKGLIKEHEACFSEEGCLYQLNHHMEVLRELCEELPSQKSQQEVKRLLKDYEQKIERLLKCASEIHMTLQPTAGGTSKNEGTITTSENRGGDPHSEAPFAKSDNQPSTE  1080

                                        |T1121 SR8
KAMEPTMKFSLASVLRPLQEESIMEKDYSASINSLLERYDTYRDILEHHLQNNKFRITSDFSSEEDRSSSCLQAKLTDLQVIKNETDARWKEFEIISLKLENHVNDIKKPFVIKERDTLK  1200

            |                                                 |N1263 SR9
ERERELQMTLNTRMESLETALRLVLPVEKASLLLCGSDLPLHKMAIQGFHLIDADRIYQHLRNIQDSIAKQIEICNRLEEPGNFVLKELHPFDLHAMQNIILKYKTQFEGMNHRVQRSED  1320

    |L1324 SR10                                                                                    |E1420 SR11
TLKALEDFLASLRTAKLSAEPVTDLSASDTQVAQENTLTVKNKEGEIHLMKDKAKHLDKCLKMLDMSFKDAERGDDTSCENLLDAFSIKLSETHGYGVQEEFTEENKLLEACIFKNNELL  1440

                                                                                    |E1525 SR12
KNIQDVQSQISKIGLKDPTVPAVKHRKKSLIRLDKVLDEYEEEKRHLQEMANSLPHFKDGREKTVNQQCQNTVVLWENTKALVTECLEQCGRVLELLKQYQNFKSILTTLIQKEESVISL  1560
```

Figure S2B: nesprin-2 boundaries

|Y1637 SR13
QASYMGKENLKKRIAEIEIVKEEFNEHLEVVDKINQVCKNLQFYLNKMKTFEEPPFEKEANIIVDRWLDINEKTEDYYENLGRALALWDKLFNLKNVIDEWTEKALQKMELHQLTEEDRE 1680

|N1739 SR14
RLKEELQVHEQKTSEFSRRVAEIQFLLQSSEIPLELQVMESSILNKMEHVQKCLTGESNCHALSGSTAELREDLDQAKTQIGMTESLLKALSPSDSLEIFTKLEEIQQQILQQKHSMILL 1800

|V1831 SR15
ENQIGCLTPELSELKKQYESVSDLFNTKKSVLQDHFSKLLNDQCKNFNDWFSNIKVNLKECFESSETKKSVEQKLQKLSDFLTLEGRNSKIKQVDSVLKHVKKHLPKAHVKELISWLVGQ 1920

|E1939 SR16 |K2037 SR17
EFELEKMESICQARAKELEDSLQQLLRLQDDHRNLRKWLTNQEEKWKGMEEPGEKTELFCQALARKREQFESVAQLNNSLKEYGFTEEEEIIMEATCLMDRYQTLLRQLSEIEEEDKLLP 2040

|Y2133 SR18
TEDQSFNDLAHDVIHWIKEIKESLMVLNSSEGKMPLEERIQKIKEIILLKPEGDARIETIMKQAESSEAPLVQKTLTDISNQWDNTLHLASTYLSHQEKLLLEGEKYLQSKEDLRLMLIE 2160

|L2244 SR19
LKKKQEAGFALQHGLQEKKAQLKIYKKFLKKAQDLTSLLKELKSQGNYLLECTKNPSFSEEPWLEIKHLHESLLQQLQDSVQNLDGHVREHDSYQVCVTDLNTTLDNFSKEFVSFSDKPV 2280

|
DQIAVEEKLQKLQELENRLSLQDGTLKKILALAKSVKQNTSSVGQKIIKDDIKSLQCKQKDLENRLASAKQEMECCLNSILKSKRSTEKKGKFTLPGREKQATSDVQESTQESAAVEKLE 2400

|D2432 SR20 |N2514 SR21
EDWEINKDSAVEMAMSKQLSLNAQESMKNTEDERKVNELQNQPLELDTMLRNEQLEEIEKLYTQLEAKKAAIKPLEQTECLNKTETGALVLHNIGYSAQHLDNLLQALITLKKNKESQYC 2520

|Q2621 SR22
VLRDFQEYLAAVESSMKALLTDKESLKVGPLDSVTYLDKIKKFIASIEKEKDSLGNLKIKWENLSNHVTDMDKKLLESQIKQLEHGWEQVEQQIQKKYSQQVVEYDEFTTLMNKVQDTEI 2640

|P2718 SR23
SLQQQQQHLQLRLKSPEERAGNQSMIALTTDLQATKHGFSVLKGQAELQMKRIWGEKEKKNLEDGINNLKKQWETLEPLHLEAENQIKKCDIRNKMKETILWAKNLLGELNPSIPLLPDD 2760

|K2832 SR24
ILSQIRKCKVTHDGILARQQSVESLAEEVKDKVPSLTTYEGSDLNNTLEDLRNQYQMLVLKSTQRSQQLEFKLEERSNFFAIIRKFQLMVQESETLIIPRVETAATEAELKHHHVTLEAS 2880

|C2934 SR25
QKELQEIDSGISTHLQELTNIYEELNVFERLFLEDQLKNLKIRTNRIQRFIQNTCNEVEHKIKFCRQFHEKTSALQEEADSIQRNELLLNQEVNKGVKEEIYNLKDRLTAIKCCILQVLK 3000

Figure S2B: nesprin-2 boundaries

|Q3037 SR26

LKKVFDYIGLNWDFSQLDQLQTQVFEKEKELEEKIKQLDTFEEEHGKYQALLSKMRAIDLQIKKMTEVVLKAPDSSPESRRLNAQILSQRIEKAKCLCDEIIKKLNENKTFDDSFKEKEI 3120

|E3143 SR27

LQIKLNAEENDKLYKVLQNMVLELSPKELDEKNCQDKLETSLHVLNQIKSQLQQPLLINLEIKHIQNEKDNCEAFQEQVWAEMCSIKAVTAIEKQREENSSEASDVETKLREFEDLQMQL 3240

|N3249 SR28 |E3353 SR29

NTSIDLRTNVLNDAYENLTRYKEAVTRAVESITSLEAIIIPYRVDVGNPEESLEMPLRKQEELESTVAHIQDLTEKLGMISSPEAKLQLQYTLQELVSKNSAMKEAFKAQETEAERYLEN 3360

|E3466 SR30

YKCYRKMEEDIYTNLSKMETVLGQSMSSLPLSYREALERLEQSKALVSNLISTKEELMKLRQILRLLRLRCTENDGICLLKIVSALWEKWLSLLEAAKEWEMWCEELKQEWKFVSEEIER 3480

|N3574 SR31

EAIILDNLQEELPEISKTKEAATTEELSELLDCLCQYGENVEKQQLLLTLLLQRIRSIQNVPESSGAVETVPAFQEITSMKERCNKLLQKVQKNKELVQTEIQERHSFTKEIIALKNFFQ 3600

|L3680 SR32

QTTTSFQNMAFQDHPEKSEQFEELQSILKKGKLTFENIMEKLRIKYSEMYTIVPAEIESQVEECRKALEDIDEKISNEVLKSSPSYAMRRKIEEINNGLHNVEKMLQQKSKNIEKAQEIQ 3720

|S3778 SR33

KKMWDELDLWHSKLNELDSEVQDIVEQDPGQAQEWMDNLMIPFQQYQQVSQRAECRTSQLNKATVKMEEYSDLLKSTEAWIENTSHLLANPADYDSLRTLSHHASTVQMALEDSEQKHNL 3840

|S3881 SR34

LHSIFMDLEDLSIIFETDELTQSIQELSNQVTALQQKIMESLPQIQRMADDVVAIESEVKSMEKRVSKIKTILLSKEIFDFSPEEHLKHGEVILENIRPMKKTIAEIVSYQVELRLPQTG 3960

|Q3987 SR35

MKPLPVFQRTNQLLQDIKLLENVTQEQNELLKVVIKQTNEWDEEIENLKQILNNYSAQFSLEHMSPDQADKLPQLQGEIERMEKQILSLNQRKEDLLVDLKATVLNLHQHLKQEQEGVER 4080

|

DRLPAVTSEEGGVAERDASERKLNRRGSMSYLAAVEEEVEESSVKSDNGDEKAEPSPQSWSSLWKHDKDMEEDRASSSSGTIVQEAYGKISTSDNSMAQILTPDSLNTEQGPECSLRPNQ 4200

|K4229 SR36

TEEGTTPPIEADTLDSSDAQGGLEPRVEKTRPEPTEVLHACKTQVAELELWLQQANVAVEPETLNADMQQVLEQQLVGCQAMLTEIEHKVAFLLETCKDQGLGDNGATQHEAEALSLKLK 4320

|

TVKCNLEKVQMMLQEKHSEDQHPTILKKSSEPEHQEALQPVNLSELESIVTERPQFSRQKDFQQQQVLELKPMEQKDFIKFIEFNAKKMWPQYCQHDNDTTQESSASNQASSPENDVPDS 4440

Figure S2B: nesprin-2 boundaries

|N4520 SR37

ILSPQGQNGDKWQYLHHELSSKIKLPLPQLVEPQVSTNMGILPSVTMYNFRYPTTEELKTYTTQLEDLRQEASNLQTQENMTEEAYINLDKKLFELFLTLSQCLSSVEEMLEMPRLYRED 4560

|N4640 SR38

GSGQQVHYETLALELKKLYLALSDKKGDLLKAMTWPGENTNLLLECFDNLQVCLEHTQAAAVCRSKSLKAGLDYNRSYQNEIKRLYHQLIKSKTSLQQSLNEISGQSVAEQLQKADAYTV 4680

|T4728 SR39

ELENAESRVAKLRDEGERLHLPYALLQEVYKLEDVLDSMWGMLRARYTELSSPFVTESQQDALLQGMVELVKIGKEKLAHGHLKQTKSKVALQAQIENHKVFFQKLVADMLLIQAYSAKI 4800

|Q4838 SR40

LPSLLQNRETFWAEQVTEVKILEEKSRQCGMKLQSLLQKWEEFDENYASLEKDLEILISTLPSVSLVEETEERLVERISFYQQIKRNIGGKHARLYQTLNEGKQLVASVSCPELEGQIAK 4920

|K4944 SR41

LEEQWLSLNKKIDHELHRLQALLKHLLSYNRDSDQLTKWLESSQHTLNYWKEQSLNVSQDLDTIRSNINNFFEFSKEVDEKSSLKTAVISIGNQLLHLKETDTATLRASLAQFEQKWTML 5040

|H5052 SR42

ITQLPDIQEKLHQLQMEKLPSRKAITEMISWMNNVEHQTSDEDSVHSPSSASQVKHLLQKHKEFRMEMDYKQWIVDFVNQSLLQLSTCDVESKRYERTEFAEHLGEMNRQWHRVHGMLNR 5160

|L5165 SR43 |Q5267 SR44

KIQHLEQLLESITESENKIQILNNWLEAQEERLKTLQKPESVISVQKLLLDCQDIENQLAIKSKALDELKQSYLTLESGAVPLLEDTASRIDELFQKRSSVLTQVNQLKTSMQSVLQEWK 5280

|N5392 SR45

IYDQLYDEVNMMTIRFWYCMEHSKPVVLSLETLRCQVENLQSLQDEAESSEGSWEKLQEVIGKLKGLCPSVAEIIEEKCQNTHKRWTQVNQAIADQLQKAQSLLQLWKAYSNAHGEAAAR 5400

|K5488 SR46

LKQQEAKFQQLANISMSGNNLAEILPPALQDIKELQHDVQKTKEAFLQNSSVLDRLPQPAESSTHMLLPGPLHSLQRAAYLEKMLLVKANEFEFVLSQFKDFGVRLESLKGLIMHEEENL 5520

|Q5590 SR47

DRLHQQEKENPDSFLNHVLALTAQSPDIEHLNEVSLKLPLSDVAVKTLQNMNRQWIRATATALERCSELQGIGLNEKFLYCCEKWIQLLEKIEEALKVDVANSLPELLEQQKTYKMLEAE 5640

| |F5705 SR48

VSINQTIADSYVTQSLQLLDTTEIENRPEFITEFSKLTDRWQNAVQGVRQRKGDVDGLVRQWQDFTTSVENLFRFLTDTSHLLSAVKGQERFSLYQTRSLIHELKNKEIHFQRRRTTCAL 5760

|E5800 SR49

TLEAGEKLLLTTDLKTKESVGRRISQLQDSWKDMEPQLAEMIKQFQSTVETWDQCEKKIKELKSRLQVLKAQSEDPLPELHEDLHNEKELIKELEQSLASWTQNLKELQTMKADLTRHVL 5880

Figure S2B: nesprin-2 boundaries

```
                                        |R5908 SR50
VEDVMVLKEQIEHLHRQWEDLCLRVAIRKQEIEDRLNTWVVFNEKNKELCAWLVQMENKVLQTADISIEEMIEKLQKDCMEEINLFSENKLQLKQMGDQLIKASNKSRAAEIDDKLNKIN   6000

                         |K6018 SR51
DRWQHLFDVIGSRVKKLKETFAFIQQLDKNMSNLRTWLARIESELSKPVVYDVCDDQEIQKRLAEQQDLQRDIEQHSAGVESVFNICDVLLHDSDACANETECDSIQQTTRSLDRRWRNI   6120

                   |W6136 SR52
CAMSMERRMKIEETWRLWQKFLDDYSRFEDWLKSAERTAACPNSSEVLYTSAKEELKRFEAFQRQIHERLTQLELINKQYRRLARENRTDTASRLKQMVHEGNQRWDNLQRRVTAVLRRL   6240

  |T6244 SR53                                                                                                        |
RHFTNQREEFEGTRESILVWLTEMDLQLTNVEHFSESDADDKMRQLNGFQQEITLNTNKIDQLIVFGEQLIQKSEPLDAVLIEDELEELHRYCQEVFGRVSRFHRRLTSCTPGLEDEKEA   6360

                                                                                                    |S6461 SR54
SENETDMEDPREIQTDSWRKRGESEEPSSPQSLCHLVAPGHERSGCETPVSVDSIPLEWDHTGDVGGSSSHEEDEEGPYYSALSGKSISDGHSWHVPDSPSCPEHHYKQMEGDRNVPPVP   6480

                                                                        |K6550 SR55
PASSTPYKPPYGKLLLPPGTDGGKEGPRVLNGNPQQEDGGLAGITEQQSGAFDRWEMIQAQELHNKLKIKQNLQQLNSDISAITTWLKKTEAELEMLKMAKPPSDIQEIELRVKRLQEIL   6600

                                                                    |F6666 SR56
KAFDTYKALVVSVNVSSKEFLQTESPESTELQSRLRQLSLLWEAAQGAVDSWRGGLRQSLMQCQDFHQLSQNLLLWLASAKNRRQKAHVTDPKADPRALLECRRELMQLEKELVERQPQV   6720

                                                                 |                                          |KASH domain
DMLQEISNSLLIKGHGEDCIEAEEKVHVIEKKLKQLREQVSQDLMALQGTQNPASPLPSFDEVDSGDQPPATSVPAPRAKQFRAVRTTEGEEETESRVPGSTRPQRSFLSRVVRAALPLQ   6840

LLLLLLLLLACLLPSSEEDYSCTQANNFARSFYPMLRYTNGPPPT   6885
```

Figure S2: nesprin-1 (A) and nesprin-2 (B) boundaries. The first residue for each SR has been indicated. The residue highlighted in gray belong to the linker region at the interface of two SRs
