# Supplementary material for: Large-Scale Modelling of the Divergent Spectrin Repeats in Nesprins: Giant Modular Proteins
Source: PLoS One. 2013 May 6;8(5):e63633. doi: 10.1371/journal.pone.0063633 (PMC3646009; doi:10.1371/journal.pone.0063633)
Supplement: Figure S3 — Percentage of conserved residue for each SR unit (A-B). Percentage of buried and exposed residues among the total conserved residue for each SRs (C-D). (PDF) [file pone.0063633.s003.pdf]

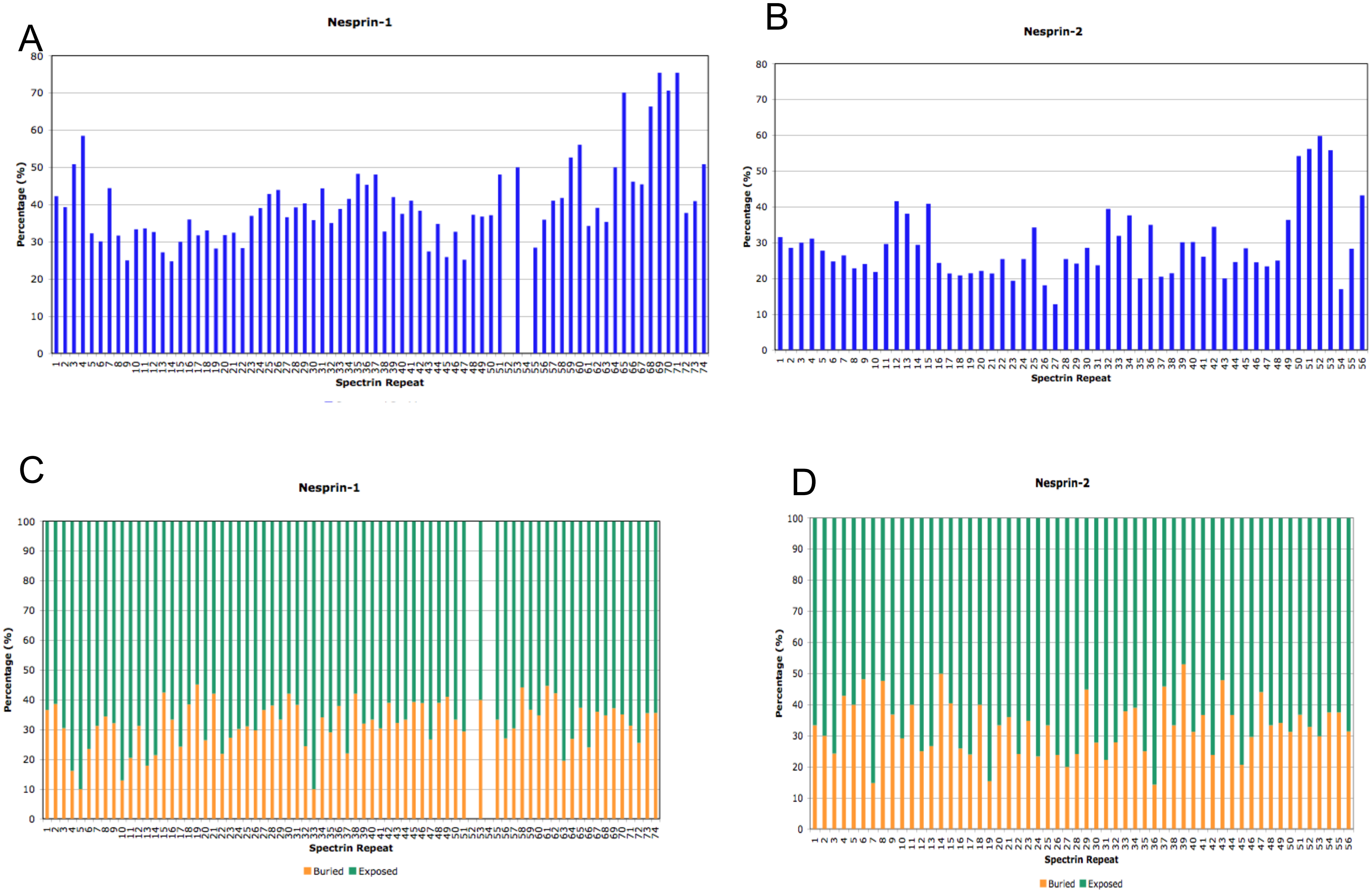

Figure S3: (A-B) Percentage of conserved residue for each SR unit. (C-D) Percentage of buried and exposed residues among the total conserved residue for each SRs
