# Supplementary material for: Large-Scale Modelling of the Divergent Spectrin Repeats in Nesprins: Giant Modular Proteins
Source: PLoS One. 2013 May 6;8(5):e63633. doi: 10.1371/journal.pone.0063633 (PMC3646009; doi:10.1371/journal.pone.0063633)
Supplement: Figure S4 — Spectroscopic characterisation of spectrin repeats SR55 and SR56. (A) 1D NMR spectra. (B) CD spectra.(C) Thermal unfolding curves measured by CD at wavelengths of 222 and 208 nm. (D) Purified SR proteins studied. (PDF) [file pone.0063633.s004.pdf]

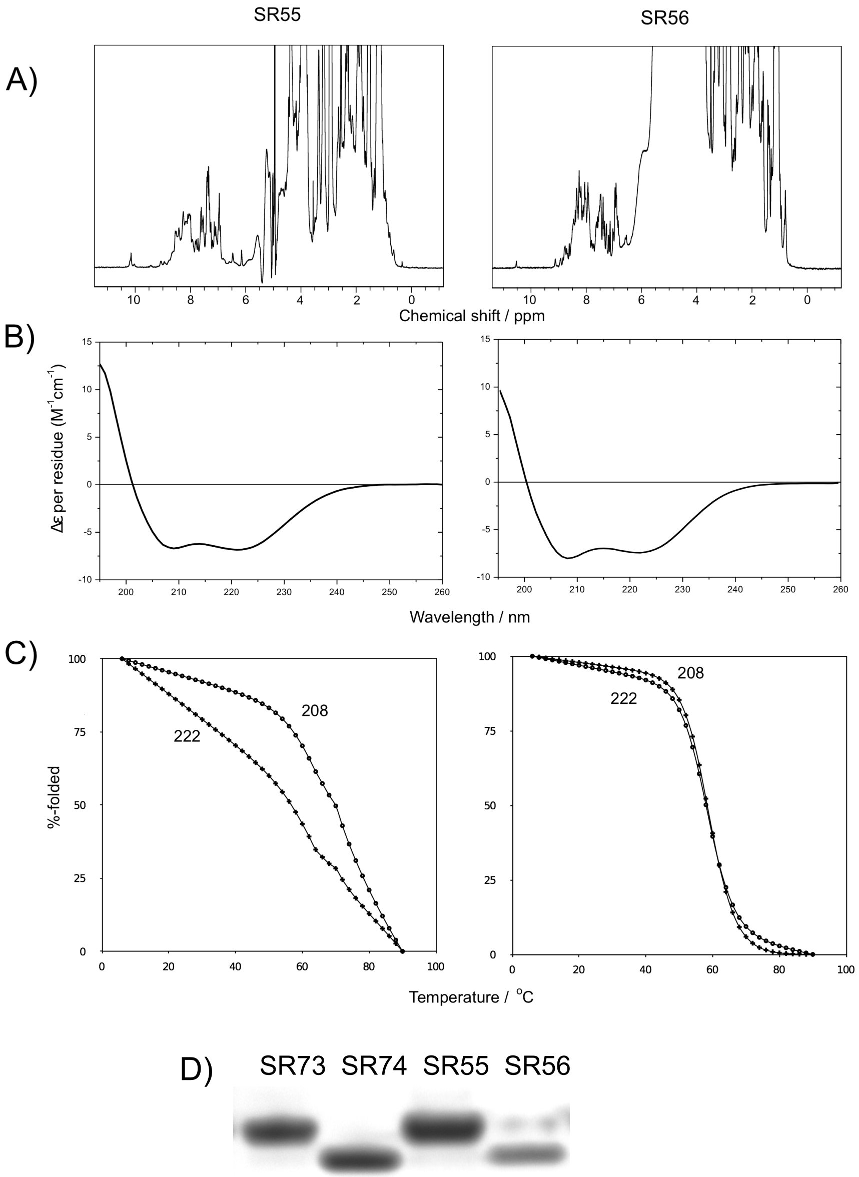

Figure S4: Spectroscopic characterisation of spectrin repeats SR55 and SR56. (A) 1D NMR spectra. (B) CD spectra.(C) Thermal unfolding curves measured by CD at wavelengths of 222 and 208nm. (D) Purified SR proteins studied.
