# Supplementary material for: Large-Scale Modelling of the Divergent Spectrin Repeats in Nesprins: Giant Modular Proteins
Source: PLoS One. 2013 May 6;8(5):e63633. doi: 10.1371/journal.pone.0063633 (PMC3646009; doi:10.1371/journal.pone.0063633)
Supplement: Figure S5 — Correlated motions of Cα residues belonging to 1S35 (A) NES1 SR70-71 and NES2SR52-53 (C). Motions with >80% correlation are indicated by lines connecting the involved residues. (PDF) [file pone.0063633.s005.pdf]

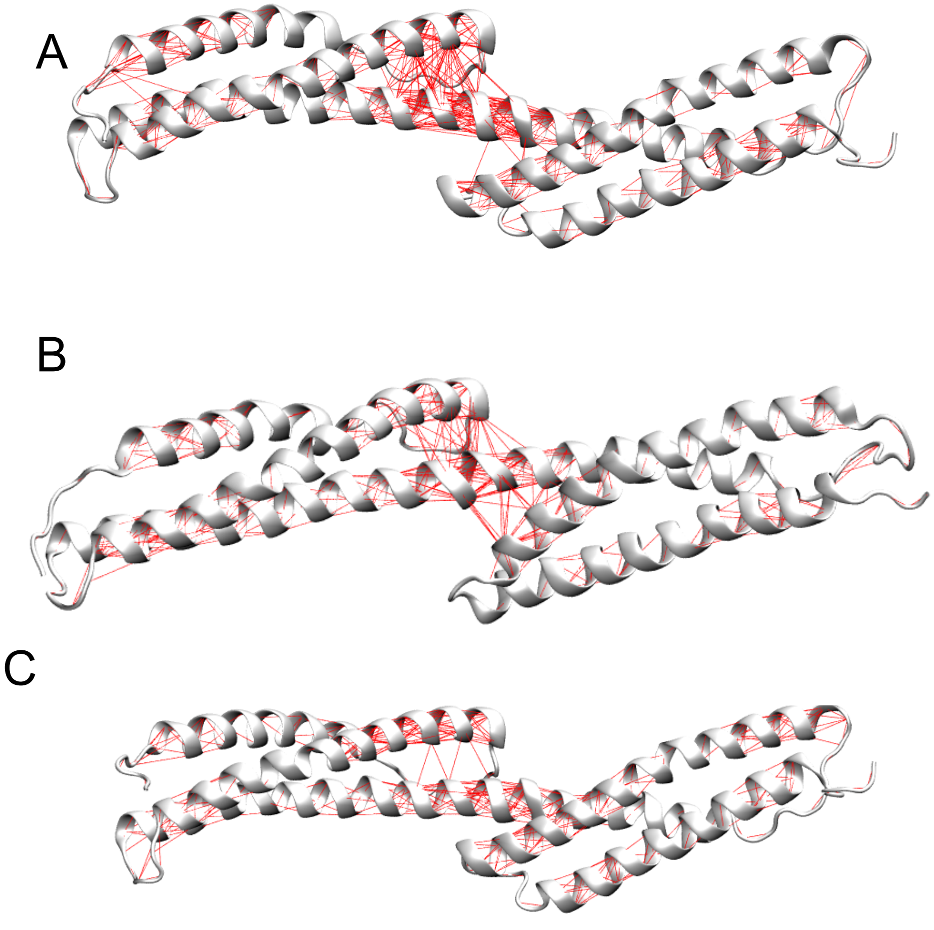

Figure S5: Correlated motions of C*α* residues belonging to 1S35 (A) ^{NES1}SR70-71 and ^{NES2}SR52-53 (C). Motions with >80% correlation are indicated by lines connecting the involved residues.
