# Supplementary material for: Large-Scale Modelling of the Divergent Spectrin Repeats in Nesprins: Giant Modular Proteins
Source: PLoS One. 2013 May 6;8(5):e63633. doi: 10.1371/journal.pone.0063633 (PMC3646009; doi:10.1371/journal.pone.0063633)
Supplement: Figure S6 — Time evolution of the secondary structure elements during MD simulations. Positions of secondary structure elements α-helices A B C and A’ B’ and C’ 5 are indicated on the y-axis and the simulation time in nanoseconds is indicated on the x-axis. Colour indicate secondary structure elements at a given time point as determined by DSSP classification. (PDF) [file pone.0063633.s006.pdf]

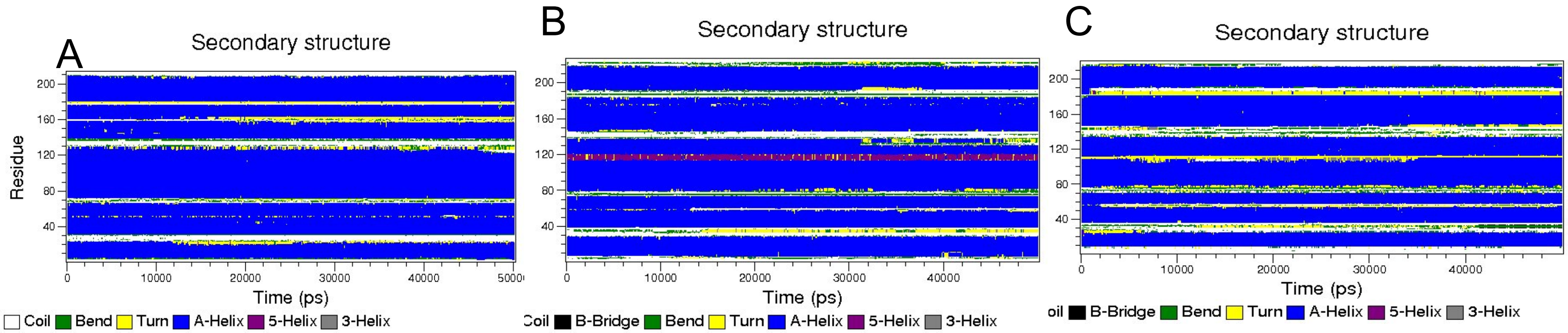

Figure S6 : Time evolution of the secondary structure elements during MD simulations. Positions of secondary structure elements α-helices A B C and A' B' and C' 5 are indicated on the y-axis and the simulation time in nanoseconds is indicated on the x-axis. Colour indicate secondary structure elements at a given time point as determined by DSSP classification.
