# Supplementary material for: Large-Scale Modelling of the Divergent Spectrin Repeats in Nesprins: Giant Modular Proteins
Source: PLoS One. 2013 May 6;8(5):e63633. doi: 10.1371/journal.pone.0063633 (PMC3646009; doi:10.1371/journal.pone.0063633)
Supplement: Figure S7 — Inner product of the first 10 eigenvector between 1S35 - NES2SR52-53 (A), 1S35 - NES1 SR70-71 (B) and NES2SR52-53-NES1SR70-71(C). (PDF) [file pone.0063633.s007.pdf]

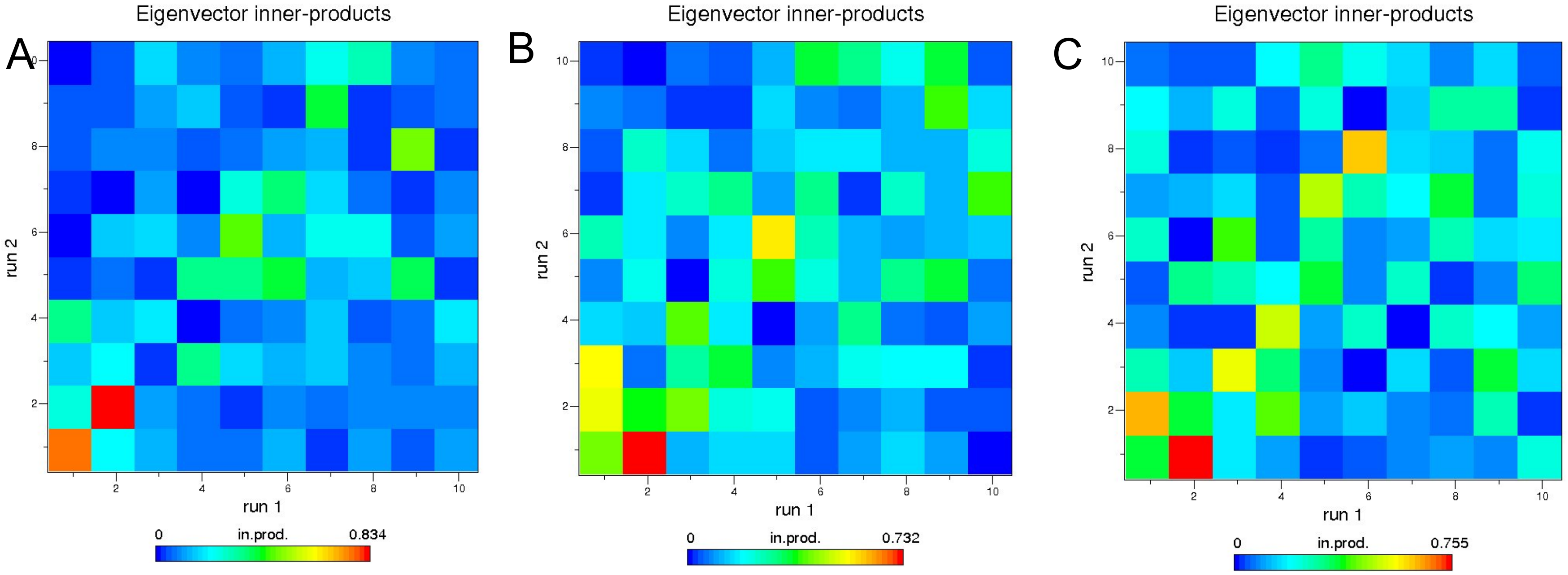

Figure S7: Inner product of the first 10 eigenvector between 1S35 - ^{NES2}SR52-53 (A), 1S35 - ^{NES1}SR70-71 (B) and ^{NES2}SR52-53- ^{NES1}SR70-71(C)
