# Supplementary material for: Large-Scale Modelling of the Divergent Spectrin Repeats in Nesprins: Giant Modular Proteins
Source: PLoS One. 2013 May 6;8(5):e63633. doi: 10.1371/journal.pone.0063633 (PMC3646009; doi:10.1371/journal.pone.0063633)
Supplement: Figure S8 — Porcupine plots of the motions corresponding to the first eigenvector of the simulations of NES1SR70-71 (A) and NES2SR52-53 (B). Each Cα atom has a cone attached pointing in the direction of motion described by the eigenvector corresponding to that atom. (PDF) [file pone.0063633.s008.pdf]

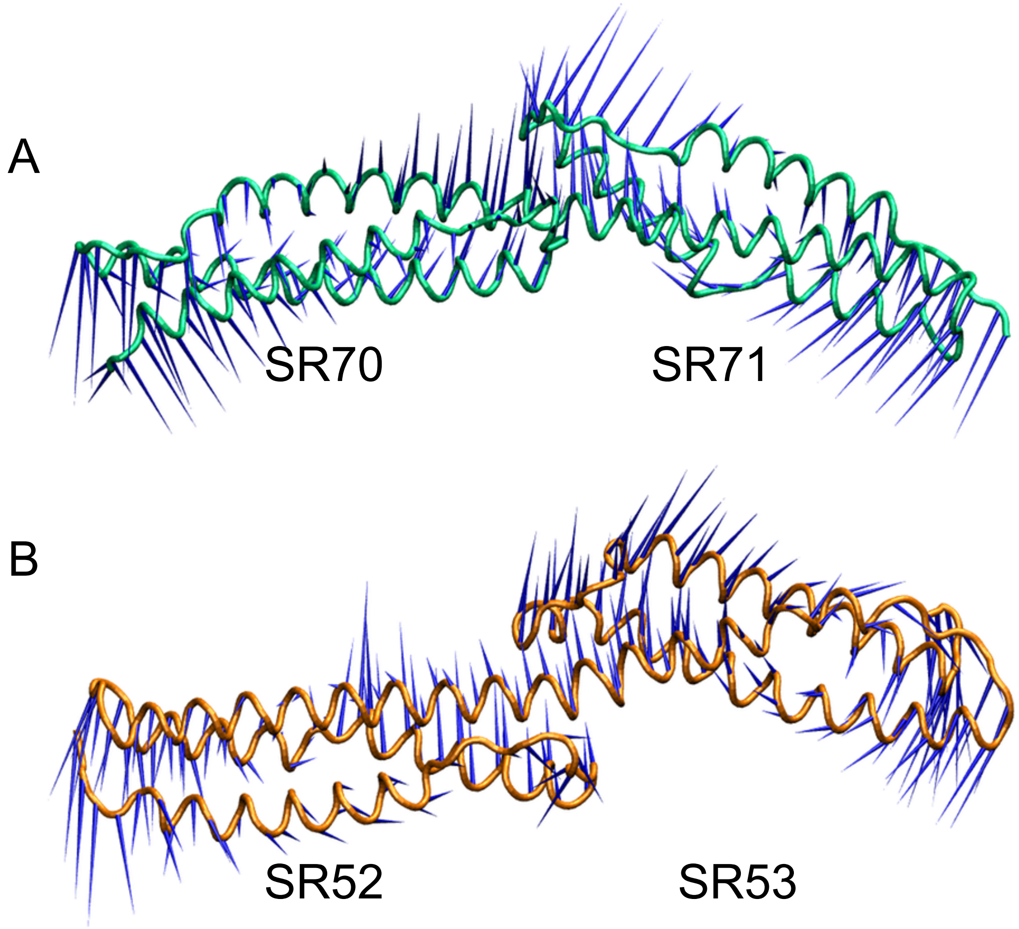

Figure S8: Porcupine plots of the motions corresponding to the first eigenvector of the simulations of ^{NES1}SR70-71 (A) and ^{NES2}SR52-53 (B). Each Cα atom has a cone attached pointing in the direction of motion described by the eigenvector corresponding to that atom.
