# Supplementary material for: Large-Scale Modelling of the Divergent Spectrin Repeats in Nesprins: Giant Modular Proteins
Source: PLoS One. 2013 May 6;8(5):e63633. doi: 10.1371/journal.pone.0063633 (PMC3646009; doi:10.1371/journal.pone.0063633)
Supplement: Figure S11 — Cladogram of Nesprin-2 SRs based on the alignment templates-targets performed with CLUSTALX2 program and web server DrawTree. The template used for each group of SRs is highlighted on the side. (PDF) [file pone.0063633.s011.pdf]

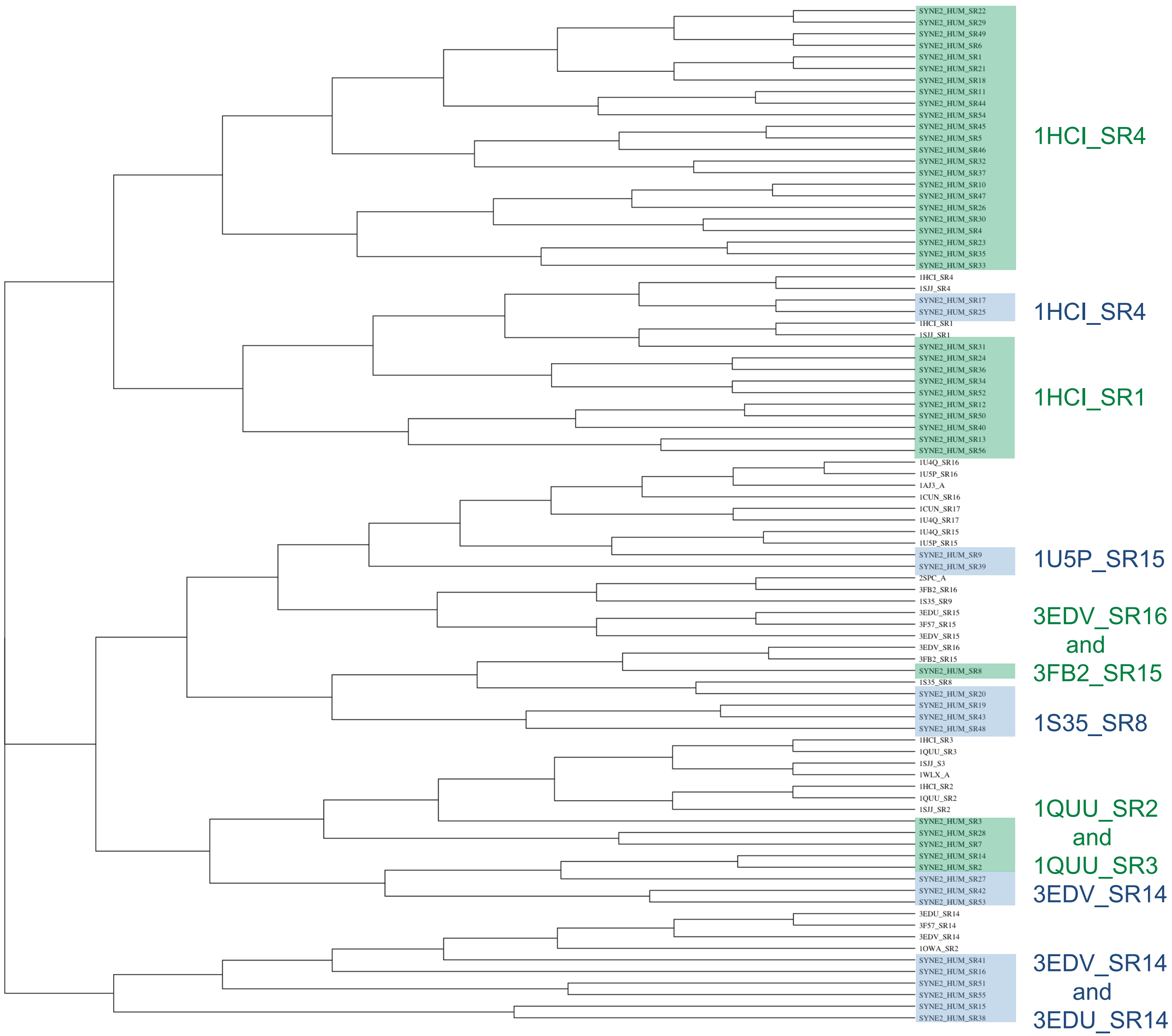

Figure S11: Cladogram of Nesprin-2 SRs based on the alignment templates-targets performed with CLUSTALX2 program and web server DrawTree. The template used for each group of SRs is highlighted on the side.
