# Supplementary material for: Large-Scale Modelling of the Divergent Spectrin Repeats in Nesprins: Giant Modular Proteins
Source: PLoS One. 2013 May 6;8(5):e63633. doi: 10.1371/journal.pone.0063633 (PMC3646009; doi:10.1371/journal.pone.0063633)
Supplement: Figure S12 — Alignment used to build the models of nesprin-1 (A) and nesprin-2 (B) SRs (PDF) [file pone.0063633.s012.pdf]

Figure S12A: Alignments used to build the models of nesprin-1 SRs

A

```
>P1;NESPRIN-1_SR1
sequence:NESPRIN-1_SR1
-------REDRVIFKEMKVWIEQFERDLT-----RAQMVESNLQDKYQSF
KHFRVQYEMKRKQIEHLIQPLH------RDGKLSLDQALVKQSWDRVTSR
LFDWHIQLDKSLPAP*
>P1;3EDV_SR14
structure:3EDV_SR14
SHMRHRLFQLNREVDDLEQWIAEREVVAGSHELGQDYEHVTMLQERFREF
ARDTGNIGQERVDTVNHLADELINSGHSDAATIAEWKDGLNEAWADLLEL
IDTRTQILAASYELH*
```

```
>P1;NESPRIN-1_SR2
sequence:NESPRIN-1_SR2
DWHIQLDKSLPAPLGTIGAWLYRAEVAL-----REEITVQQVHEETANTI
QRKLEQHKDLLQNTDAHKRAFHEIYRTRSVN---GIPVPPDQLEDMAERF
HFVSSTSELHLMKMEFLELK----*
>P1;1QUU_SR3
structure:1QUU_SR3
ETIDQLHLEFAKRAAPFNNWMEGAMEDLQDMFIVHSIEEIQSLITAHEQF
KATLPEADGERQSIMAIQNEVEKVIQSYNIRISSSNPYSTVTMDELRTKW
DKVKQLVPIRDQSLQEELARQHAN*
```

```
>P1;NESPRIN-1_SR3
sequence:NESPRIN-1_SR3
--MEFLELKYRLLS--LLVLAESKLKSWIIKYGR--RESVEQLLQNYVSF
IENSKFFEQYEVTYQILKQTAEMYVKADGSVEEAEN--VMKFMNETTAQW
RNLSVEVRSVRSMLEEVISNWDRY*
>P1;1QUU_SR3
structure:1QUU_SR3
ETIDQLHLEFAKRAAPFNNWMEGAMEDLQDMFIVHSIEEIQSLITAHEQF
KATLPEADGERQSIMAIQNEVEKVIQSYNIRISSSNPYSTVTMDELRTKW
DKVKQLVPIRDQSLQEELARQHAN*
```

```
>P1;NESPRIN-1_SR4
sequence:NESPRIN-1_SR4
E-VISNWDRYGNTVASLQAWLEDAEKMLNQSE------------NAKKDF
FRNLPHWIQQHTAMNDAGNFLIETCDEMVSRD------LKQQLLLLNGRW
RELFMEVKQYAQADEMDRMKKEYT*
>P1;1QUU_SR3
structure:1QUU_SR3
ETIDQLHLEFAKRAAPFNNWMEGAMEDLQDMFIVHSIEEIQSLITAHEQF
KATLPEADGERQSIMAIQNEVEKVIQSYNIRISSSNPYSTVTMDELRTKW
DKVKQLVPIRDQSLQEELARQHAN*
```

Figure S12A: Alignments used to build the models of nesprin-1 SRs

```
>P1;NESPRIN-1_SR5
sequence:NESPRIN-1_SR5
DEMDRMKKEYTDCVVTLSAFATEAHKKLSEPLEVS-FMNVKLLIQDLEDI
EQRVPVMDAQYKIITKTAHLITKESPQEEGKEMFATMSKLKEQL
TKVKECYSPLLYESQQLLIPLEELEKQMTSF*
>P1;1QUU_SR3
structure:1QUU_SR3
ETIDQLHLEFAKRAAPFNNWMEGAMEDLQDMFIVHSIEEIQSLITAHEQF
KATLPEADGERQSIMAIQNEVEKVIQSYNIRISSSNPYSTVTMDELRTKW
DKVKQLVPIRDQSLQEELARQHAN-------*
```

```
>P1;NESPRIN-1_SR6
sequence:NESPRIN-1_SR6
--PLEELEKQMTSFYDSLGKINEIITVLEREAQSSALFKQKHQELLACQE
NCKKTLTLIEKGSQSVQKFVTLSNVLKHFDQTRLQRQIADIHVAFQSMVK
KTGDWKKHVETNSRL*
>P1;1S35_SR9
structure:1S35_SR9
--GFQEFQKDAKQAEAILSNQEYTLAHLEP-----PDSLEAAEAGIRKFE
DFLGSMENNRDKVLSPVDSGNKLVAEGNLYSDKIKEKVQLIEDRHRKNNE
KAQEASVLLRDN---*
>P1;1U5P_SR15
structure:1U5P_SR15
ANKQQNFNTGIKDFDFWLSEVEALLASEDY-----GKDLASVNNLLKKHQ
LLEADISAHEDRLKDLNSQADSLMTSSAFDTSQVKDKRETINGRFQRIKS
MAAARRAKLNESHRL*
```

```
>P1;NESPRIN-1_SR7
sequence:NESPRIN-1_SR7
-TNSRLMKKFEESRAELEKVLRIAQEGL-----EE-KGDPEELLRRHTEF
FSQL--DQRVLNAFLKACDELTDILPEQEQQGLQE------AVRKLHKQW
KDLQGEAPYHLLHLKIDVEKNRF-*
>P1;1QUU_SR3
structure:1QUU_SR3
ETIDQLHLEFAKRAAPFNNWMEGAMEDLQDMFIVHSIEEIQSLITAHEQF
KATLPEADGERQSIMAIQNEVEKVIQSYNIRISSSNPYSTVTMDELRTKW
DKVKQLVPIRDQSLQEELARQHAN*
```

Figure S12A: Alignments used to build the models of nesprin-1 SRs

```
>P1;NESPRIN-1_SR8
sequence:NESPRIN-1_SR8
----DVEKNRFLASVEECRTELDRETKLMP---QEGSEKIIKEHRVFFSD
KGPHHLCEKRLQLIEELCVKL--PVRDPVRDTPGTCHVTLKELRAAIDST
YRKLMEDPDK*
>P1;1S35_SR9
structure:1S35_SR9
--GFQEFQKDAKQAEAILSNQEYTLAHLEPPDSLEAAEAGIRKFEDFLGS
MENNRDKVLSPVDSGNKLVAEGNLYSDKIKEKVQLIEDRHRKNNEKAQEA
SVLLRDN---*
>P1;1U5P_SR15
structure:1U5P_SR15
ANKQQNFNTGIKDFDFWLSEVEALLASEDYGKDLASVNNLLKKHQLLEAD
ISAHEDRLKDLNSQADSLMTSSAFDTSQVKDKRETINGRFQRIKSMAAAR
RAKLNESHRL*


>P1;NESPRIN-1_SR9
sequence:NESPRIN-1_SR9
PDKWKDYTSRFSEFSSWISTNETQLKGIKGEAIDTANHGEVKRAVEEIRN
GVTKRGETLSWLKSRLKVLTEVSSENEAQKQGDELAKLSSSFKALVTLLS
EVEKMLSNFGDC*
>P1;1S35_SR9
structure:1S35_SR9
--GFQEFQKDAKQAEAILSNQEYTLAHLEPP-DSLEAAEAGIRKFEDFLG
SMENNRDKVLSPVDSGNKLVAEG-NLYSDKIKEKVQLIEDRHRKNNEKAQ
EASVLLRDN---*
>P1;1U5P_SR15
structure:1U5P_SR15
ANKQQNFNTGIKDFDFWLSEVEALLASEDYG-KDLASVNNLLKKHQLLEA
DISAHEDRLKDLNSQADSLMTSS-AFDTSQVKDKRETINGRFQRIKSMAA
ARRAKLNESHRL*


>P1;NESPRIN-1_SR10
sequence:NESPRIN-1_SR10
ELISGSKEVQ---EQAEKILDTE----NLFEAQQLLLHHQQKTKRISAKK
RDVQQQIAQAQQGEGG---LPDRGHEELRKLESTLDGLERSRERQERRIQ
VTL*
>P1;3EDV_SR16
structure:3EDV_SR16
RFFSMVRDLMLWMEDVIRQIEAQEKPRDVSSVELLMNNHQGIKAEIDARN
DSFTTCIELGKSLLARKHYASEEIKEKLLQLTEKRKEMIDKWEDRWEWLR
L--*
```

Figure S12A: Alignments used to build the models of nesprin-1 SRs

```
>P1;NESPRIN-1_SR11
sequence:NESPRIN-1_SR11
QVTLRKWERFETNKETVVRYLFQTGSSHERFLSFSSLESLSSELEQTKEF
SKRTESIAVQAENLVKEASEI------PLGPQNKQLLQQQAKSIKEQVKK
LEDTLEEDIKTMEMVKTKWDHF--------*
>P1;1QUU_SR3
structure:1QUU_SR3
ETIDQLHLEFAKRAAPFNNWMEGAMEDLQDMFIVHSIEEIQSLITAHEQF
KATLPEADGERQSIMAIQNEV------EKVIQSYNIRISSSNPYSTVTMD
ELRTKWDKVKQLVPIRDQSLQEELARQHAN*


>P1;NESPRIN-1_SR12
sequence:NESPRIN-1_SR12
KTKWDHFGSNFETLSVWITEKEKELNALE--TSSSAMDMQISQIKVTIQE
IESKLSSIVGLEEEAQSFAQFVTTGESARIKAKLTQIRRYGEELREHAQC
LEGTILGHL--*
>P1;1S35_SR9
structure:1S35_SR9
--GFQEFQKDAKQAEAILSNQEYTLAHLEPPDSLEAAEAGIRKFEDFLGS
MENNRDKVLSPVDSGNKLVAEGNL-YSDKIKEKVQLIEDRHRKNNEKAQE
ASVLLRDN---*
>P1;1U5P_SR15
structure:1U5P_SR15
ANKQQNFNTGIKDFDFWLSEVEALLASEDYGKDLASVNNLLKKHQLLEAD
ISAHEDRLKDLNSQADSLMTSSAF-DTSQVKDKRETINGRFQRIKSMAAA
RRAKLNESHRL*


>P1;NESPRIN-1_SR13
sequence:NESPRIN-1_SR13
LSQQQKFEENLRKIQQSVSEFEDKLAVPIKICSSATETYKVLQEHMDLCQ
ALESLSSAITAFSASARKVVN-----RDSCVQEAAALQQQYEDILRRAKE
RQTALENL*
>P1;1S35_SR9
structure:1S35_SR9
--GFQEFQKDAKQAEAILSNQEYTLAHL-EPPDSLEAAEAGIRKFEDFLG
SMENNRDKVLSPVDSGNKLVAEGNLYSDKIKEKVQLIEDRHRKNNEKAQE
ASVLLRDN*
```

Figure S12A: Alignments used to build the models of nesprin-1 SRs

```
>P1;NESPRIN-1_SR14
sequence:NESPRIN-1_SR14
LAHWQRLEKELSSFLTWLERGEAKASSPEMDISADRVKVEGELQLIQALQ
NEVVSQASFYSKLLQLKESLFSVASKDDVKMMKLHLEQLDERWRDLPQII
NKRINFLQSVVAE*
>P1;1S35_SR9
structure:1S35_SR9
--GFQEFQKDAKQAEAILSNQEYTLAHLE--PPDSLEAAEAGIRKFEDFL
GSMENNRDKVLSPVDSGNKLVAEG-NLYSDKIKEKVQLIEDRHRKNNEKA
QEASVLLRDN---*
>P1;1U5P_SR15
structure:1U5P_SR15
ANKQQNFNTGIKDFDFWLSEVEALLASED--YGKDLASVNNLLKKHQLLE
ADISAHEDRLKDLNSQADSLMTSS-AFDTSQVKDKRETINGRFQRIKSMA
AARRAKLNESHRL*
```

```
>P1;NESPRIN-1_SR15
sequence:NESPRIN-1_SR15
VAEHQQFDELLLSFSVWIKLFLSELQTTS-EISIMDHQVALTRHKDHAAE
VESKKGELQSLQGHLAKLGSLGRAEDLHLLQGKAEDCFQLFEEASQVVER
RQLALSHLAEF*
>P1;1S35_SR9
structure:1S35_SR9
--GFQEFQKDAKQAEAILSNQEYTLAHLEPPDSLEAAEAGIRKFEDFLGS
MENNRDKVLSPVDSGNKLVAEGNLYSDKIKE-KVQLIEDRHRKNNEKAQE
ASVLLRDN---*
>P1;1U5P_SR15
structure:1U5P_SR15
ANKQQNFNTGIKDFDFWLSEVEALLASEDYGKDLASVNNLLKKHQLLEAD
ISAHEDRLKDLNSQADSLMTSSAFDTSQVKD-KRETINGRFQRIKSMAAA
RRAKLNESHRL*
```

```
>P1;NESPRIN-1_SR16
sequence:NESPRIN-1_SR16
-----LSGILRQLRQTVEATNSMNKNESDLIEKDLNDALQNAKALESAAV
SLDGILSKAQYHLKIGSSEQRT---SCRATADQLCGEVERIQNLLGTKQS
EADALAVL---*
>P1;1S35_SR9
structure:1S35_SR9
---GFQEFQKDAKQAEAILSNQEYTLAHLEPPDSLEAAEAGIRKFEDFLG
SMENNRDKVLSPVDSGNKLVAEGNLYSDKIKEKVQLIEDRHRKNNEKAQE
ASVLLRDN---*
>P1;1U5P_SR15
structure:1U5P_SR15
-ANKQQNFNTGIKDFDFWLSEVEALLASEDYGKDLASVNNLLKKHQLLEA
DISAHEDRLKDLNSQADSLMTSSAFDTSQVKDKRETINGRFQRIKSMAAA
RRAKLNESHRL*
```

Figure S12A: Alignments used to build the models of nesprin-1 SRs

```
>P1;NESPRIN-1_SR17
sequence:NESPRIN-1_SR17
AVLKKAFQDQKEELLKSIEDIEERTDKERLKEPTRQALQQRLRVFNQLED
ELNSHEHELCWLKDKAKQIAQKDVAFAPEVDREINRLEVTWDDTKRLIHE
NQGQ-------*
>P1;1S35_SR9
structure:1S35_SR9
--GFQEFQKDAKQAEAILSNQEYTLAHLEPPD-SLEAAEAGIRKFEDFLG
SMENNRDKVLSPVDSGNKLVAEGNLYSDKIKEKVQLIEDRHRKNNEKAQE
ASVLLRDN---*
>P1;1U5P_SR15
structure:1U5P_SR15
ANKQQNFNTGIKDFDFWLSEVEALLASEDYGK-DLASVNNLLKKHQLLEA
DISAHEDRLKDLNSQADSLMTSSAFDTSQVKDKRETINGRFQRIKSMAAA
RRAKLNESHRL*
```

```
>P1;NESPRIN-1_SR18
sequence:NESPRIN-1_SR18
CGLIDLMREYQNLKSAVSKVLENASSVIVTRTTIKDQEDLKWAFSKHETA
KNKMNYKQKDLDNFTSKGKHLLSELKKIHSSDFSLVKTDMESTVDKWLDV
SEKLEENMDRLRVSLSIW-*
>P1;1HCI_SR4
structure:1HCI_SR4
HANERLRRQFAAQANAIGPWIQNKMEEIARSS-IQITGALEDQMNQLKQY
EHNIINYKNNIDKLEGDHQLIQEALV----FDNKHTNYTMEHIRVGWELL
LTTIARTINEVETQILTRD*
```

```
>P1;NESPRIN-1_SR19
sequence:NESPRIN-1_SR19
LSIWDDVLSTRDEIEGWSNNCVPQMAENI--SNLDNHLRAEELLKEFESE
VKNKALRLEELHSKVNDLKELTKNLETPPDLQFIEADLMQKLEHAKEITE
VAKGTLKDFTAQ*
>P1;1S35_SR9
structure:1S35_SR9
--GFQEFQKDAKQAEAILSNQEYTLAHLEPPDSLEAAEAGIRKFEDFLGS
MENNRDKVLSPVDSGNKLVAEGNLYS--DKIKEKVQLIEDRHRKNNEKAQ
EASVLLRDN---*
>P1;1U5P_SR15
structure:1U5P_SR15
ANKQQNFNTGIKDFDFWLSEVEALLASEDYGKDLASVNNLLKKHQLLEAD
ISAHEDRLKDLNSQADSLMTSSAFDT--SQVKDKRETINGRFQRIKSMAA
ARRAKLNESHRL*
```

Figure S12A: Alignments used to build the models of nesprin-1 SRs

```
>P1;NESPRIN-1_SR20
sequence:NESPRIN-1_SR20
TAQSTQVEKFINDITTWFTKVEESLMN----CAQNETCEALKKVKDIQKE
LQSQQSNISSTQENLNSLCRKYHSAELESLGRAMTGLIKKHEAVSQLCSK
TQASLQESLEK*
>P1;1S35_SR9
structure:1S35_SR9
--GFQEFQKDAKQAEAILSNQEYTLAHLEPPDSLEAAEAGIRKFEDFLGS
MENNRDKVLSPVDSGNKLVAE-GNLYSDKIKEKVQLIEDRHRKNNEKAQE
ASVLLRDN---*
>P1;1U5P_SR15
structure:1U5P_SR15
ANKQQNFNTGIKDFDFWLSEVEALLASEDYGKDLASVNNLLKKHQLLEAD
ISAHEDRLKDLNSQADSLMTS-SAFDTSQVKDKRETINGRFQRIKSMAAA
RRAKLNESHRL*


>P1;NESPRIN-1_SR21
sequence:NESPRIN-1_SR21
SLQESLEKHFSESMQEFQEWFLGAKAAAKESSDRTGDSKVLEAKLHDLQN
ILDSVSDGQSKLDAVTQEGQTLYAHLSKQIVSSIQEQITKANEEFQAFLK
QCLKDKQALQDCASELG*
>P1;1HCI_SR4
structure:1HCI_SR4
HANERLRRQFAAQANAIGPWIQNKMEEIARSS--IQITGALEDQMNQLKQ
YEHNIINYKNNIDKLEGDHQLIQEAL-VFDNKHTNYTMEHIRVGWELLLT
TIARTINEVETQILTRD*


>P1;NESPRIN-1_SR22
sequence:NESPRIN-1_SR22
ASELGSFEDQHRKLNLWIHEMEERFNTENLGESKQHIPEKKNEVHKVEMF
LGELLAARESLDKLSQRGQLLSEEGH-GAGQEGRLCSQLLTSHQNLLRMT
KEKLRSCQVALQEH*
>P1;1S35_SR9
structure:1S35_SR9
--GFQEFQKDAKQAEAILSNQEYTLAHLEPPDS---LEAAEAGIRKFEDF
LGSMENNRDKVLSPVDSGNKLVAEGNLYSDKIKEKVQLIEDRHRKNNEKA
QEASVLLRDN----*


>P1;NESPRIN-1_SR23
sequence:NESPRIN-1_SR23
QVALQEHEALEEALQSMWFWVKAIQDRLACAESTLGSKDTLEKRLSQIQD
ILLMKGEGEVKLNMAIGKGEQALRSSNKEGQRV------IQTQLETLKEV
WADIMSSSVHAQSTLESVISQWNDY*
>P1;1QUU_SR3
structure:1QUU_SR3
ETIDQLHLEFAKRAAPFNNWMEGAMEDLQDMFIVH-SIEEIQSLITAHEQ
FKATLPEADGERQSIMAIQNEVEKVIQSYNIRISSSNPYSTVTMDELRTK
WDKVKQLVPIRDQSLQEELARQHAN*
```

Figure S12A: Alignments used to build the models of nesprin-1 SRs

```
>P1;NESPRIN-1_SR24
sequence:NESPRIN-1_SR24
IISQWNDYVERKNQLEQWMESVDQKIEHPLQPQPGLKEKFVLLDHLQSILS
EAEDHTRALHRLIAKSRELYEKTE-DESFKDTAQEELKTQFNDIMTVAKE
KMRKVEEIVKD*
>P1;1S35_SR9
structure:1S35_SR9
--GFQEFQKDAKQAEAILSNQEYTLAH-LEPPDSLEAAEAGIRKFEDFLG
SMENNRDKVLSPVDSGNKLVAEGNLYSDKIKEKVQLIEDRHRKNNEKAQE
ASVLLRDN---*
>P1;1U5P_SR15
structure:1U5P_SR15
ANKQQNFNTGIKDFDFWLSEVEALLAS-EDYGKDLASVNNLLKKHQLLEA
DISAHEDRLKDLNSQADSLMTSSAFDTSQVKDKRETINGRFQRIKSMAAA
RRAKLNESHRL*
```

```
>P1;NESPRIN-1_SR25
sequence:NESPRIN-1_SR25
VKDHLMYLDAVHEFTDWLHSAKEELHRWSDMSGDSSATQKKLSKIKELID
SREIGASRLSRVESLAPEVKQNTTASGCELMHTEMQALRADWKQWEDSVF
QTQSC-------*
>P1;1S35_SR9
structure:1S35_SR9
--GFQEFQKDAKQAEAILSNQEYTLAHL-EPPDSLEAAEAGIRKFEDFLG
SMENNRDKVLSPVDSGNKLVAE-GNLYSDKIKEKVQLIEDRHRKNNEKAQ
EASVLLRDN---*
>P1;1U5P_SR15
structure:1U5P_SR15
ANKQQNFNTGIKDFDFWLSEVEALLASE-DYGKDLASVNNLLKKHQLLEA
DISAHEDRLKDLNSQADSLMTS-SAFDTSQVKDKRETINGRFQRIKSMAA
ARRAKLNESHRL*
```

```
>P1;NESPRIN-1_SR26
sequence:NESPRIN-1_SR26
---AQLEQALEQFSALLKTWAQQLTLLEGKNTDEE---IVECWHKGQEIL
DALQKAEPRTEDLKSQLNELCRFSRDLSTYSG-KVSGLIKEYNCLCLQAS
KGCQNKEQILQQRF*
>P1;1HCI_SR4
structure:1HCI_SR4
HANERLRRQFAAQANAIGPWIQNKMEEIARSSIQITGALEDQMNQLKQYE
HNIINYKNNIDKLEGDHQLIQEALVFDNKHTNYTMEHIRVGWELLLTTIA
RTINEVETQILTRD*
```

Figure S12A: Alignments used to build the models of nesprin-1 SRs

```
>P1;NESPRIN-1_SR27
sequence:NESPRIN-1_SR27
QILQQRFRKAFRDFQQWLVNAKITTAKCFDIPQNISEVSTSLQKIQEFLS
ESENGQHKLNMMLSKGELLSTLLTKEKAKGIQAKVTAAKEDWKNFHSNLH
QKESALENLKIQ*
>P1;1S35_SR9
structure:1S35_SR9
--GFQEFQKDAKQAEAILSNQEYTLAHL-EPPDSLEAAEAGIRKFEDFLG
SMENNRDKVLSPVDSGNKLVAE-GNLYSDKIKEKVQLIEDRHRKNNEKAQ
EASVLLRDN---*
>P1;1U5P_SR15
structure:1U5P_SR15
ANKQQNFNTGIKDFDFWLSEVEALLASE-DYGKDLASVNNLLKKHQLLEA
DISAHEDRLKDLNSQADSLMTS-SAFDTSQVKDKRETINGRFQRIKSMAA
ARRAKLNESHRL*
```

```
>P1;NESPRIN-1_SR28
sequence:NESPRIN-1_SR28
KIQMKDFEVSAEPIQDWLSKTEKMVHESSNRLYDLPAKRREQQKLQSVLE
EIHCYEPQLNRLKEKAQQLWEG-QAASKSFRHRVSQLSSQYLALSNLTKE
KVSRLDRI*
>P1;1S35_SR9
structure:1S35_SR9
--GFQEFQKDAKQAEAILSNQEYTLAHLEPPDS-LEAAEAGIRKFEDFLG
SMENNRDKVLSPVDSGNKLVAEGNLYSDKIKEKVQLIEDRHRKNNEKAQE
ASVLLRDN*
```

```
>P1;NESPRIN-1_SR29
sequence:NESPRIN-1_SR29
DRIVAEHNQFSLGIKELQDWMTDAIHMLDSYCHPTSDKSVLDSRTLKLEA
LLSVKQEKEIQMKMIVTRGESVLQNTSPEGIPT------IQQQLQSVKDM
WASLLSAGIRCKSQLEGALSKWTSY*
>P1;1QUU_SR3
structure:1QUU_SR3
ETIDQLHLEFAKRAAPFNNWMEGAMEDLQDMFIVH-SIEEIQSLITAHEQ
FKATLPEADGERQSIMAIQNEVEKVIQSYNIRISSSNPYSTVTMDELRTK
WDKVKQLVPIRDQSLQEELARQHAN*
```

Figure S12A: Alignments used to build the models of nesprin-1 SRs

```
>P1;NESPRIN-1_SR30
sequence:NESPRIN-1_SR30
LSKWTSYQDGVRQFSGWMDSMEANLNESERQHAELRDKTTMLGKAKLLNE
EVLSYSSLLETIEVKGAGMTE-----HYVTQLELQDLQERYRAIQERAKE
AVTKSEKLVRL*
>P1;1S35_SR9
structure:1S35_SR9
--GFQEFQKDAKQAEAILSNQEYTLA-HLEPPDSLEAAEAGIRKFEDFLG
SMENNRDKVLSPVDSGNKLVAEGNLYSDKIKEKVQLIEDRHRKNNEKAQE
ASVLLRDN---*
>P1;1U5P_SR15
structure:1U5P_SR15
ANKQQNFNTGIKDFDFWLSEVEALLA-SEDYGKDLASVNNLLKKHQLLEA
DISAHEDRLKDLNSQADSLMTSSAFDTSQVKDKRETINGRFQRIKSMAAA
RRAKLNESHRL*
```

```
>P1;NESPRIN-1_SR31
sequence:NESPRIN-1_SR31
VRLHQEYQRDLKAFEVWLGQEQEKLDQYSVLEGDAHTHETTLRDLQELQV
HCAEGQALLNSVLHTREDVIPSG--IPQAEDRALESLRQDWQAYQHRLSE
TRTQFNNV*
>P1;1S35_SR9
structure:1S35_SR9
--GFQEFQKDAKQAEAILSNQEYTLAHLEP-PDSLEAAEAGIRKFEDFLG
SMENNRDKVLSPVDSGNKLVAEGNLYSDKIKEKVQLIEDRHRKNNEKAQE
ASVLLRDN*
```

```
>P1;NESPRIN-1_SR32
sequence:NESPRIN-1_SR32
NNVVNKLRL-MEQKFQQVDEWLKTAEEKVSPRTRRQSNRATKEIQLHQMK
KWHEEVTAYRDEVEEVGARAQEILDESHVNSR--------MGCQATQLTS
RYQALLLQVLEQIKFLEEEIQSLEES*
>P1;1QUU_SR3
structure:1QUU_SR3
-ETIDQLHLEFAKRAAPFNNWMEGAMEDLQ-DMFIVHSIEEIQSLITAHE
QFKATLPEADGERQSIMAIQNEVEKVIQSYNIRISSSNPYSTVTMDELRT
KWDKVKQLVPIRDQSLQEELARQHAN*
```

```
>P1;NESPRIN-1_SR33
sequence:NESPRIN-1_SR33
--------YGSTHKNFKNVATKIDKVDTVMMGKKLKTLEVLLKDMEKGHS
LLKSAREKGERAVKYLEEGEAERL-------RKEIHDHMEQLKELTSTVR
KEHMTLEKGLHLAKEFSD*
>P1;3EDV_SR14
structure:3EDV_SR14
-SHMRHRLFQLNREVDDLEQWIAEREVVAGSHELGQDYEHVTMLQERFRE
FARDTGNIGQERVDTVN-HLADELINSGHSDAATIAEWKDGLNEAWADLL
ELIDTRTQILAASYELH-*
```

Figure S12A: Alignments used to build the models of nesprin-1 SRs

```
>P1;NESPRIN-1_SR34
sequence:NESPRIN-1_SR34
LHLAKEFSDKCKALTQWIAEYQEILHVPEEPKMELYEKKAQLSKYKSLQQ
TVLSHEPSVKSVREKGEALLEL--VQDVTLKDKIDQLQSDYQDLCSIGKE
HVFSLEAK*
>P1;1S35_SR9
structure:1S35_SR9
--GFQEFQKDAKQAEAILSNQEYTLAHLE-PPDSLEAAEAGIRKFEDFLG
SMENNRDKVLSPVDSGNKLVAEGNLYSDKIKEKVQLIEDRHRKNNEKAQE
ASVLLRDN*


>P1;NESPRIN-1_SR35
sequence:NESPRIN-1_SR35
VKDHEDYNSELQEVEKWLLQMSGRLVAPDLLETSSLETITQQLAHHKAMM
EEIAGFEDRLNNLQMKGDTLIGQCADHLQAKLKQNVHAHLQGTKDSYSAI
CSTAQRMYQSLEHE*
>P1;1S35_SR9
structure:1S35_SR9
--GFQEFQKDAKQAEAILSNQEYTLAHL--EPPDSLEAAEAGIRKFEDFL
GSMENNRDKVLSPVDSGNKLVAEGNLY-----SDKIKEKVQLIEDRHRKN
NEKAQEASVLLRDN*


>P1;NESPRIN-1_SR36
sequence:NESPRIN-1_SR36
QSLEHELQKHVSRQDTLQQCQAWLSAVQPDLEPSPQPPLSRAEAIKQVKH
FRALQEQARTYLDLLCSMCDLSNASVKTTAKDIQQTEQTIEQKLVQAQNL
TQGWEEIKHLKSELWIYLQDADQQLQNM*
>P1;1QUU_SR3
structure:1QUU_SR3
ETIDQ-LHLE--FAKRAAPFNNWMEGAMEDLQ-DMFIVHSIEEIQSLITA
HEQFKATLPEADGERQSIMAIQNEVEKVIQSYNIRISSSNPYSTVTMDEL
RTKWDKVKQLVPIRDQSLQEELARQHAN*


>P1;NESPRIN-1_SR37
sequence:NESPRIN-1_SR37
--------SELWIYLQDADQQLQNMKRRHSE-LE-------LNIAQNMVS
QVKDFV-----KKLQSKQASVNTIIEKVNKLTKKEES---P-EHKEINHL
NDQWLDLCRQSNNLCLQREEDLQRTRDYH*
>P1;1HCI_SR1
structure:1HCI_SR1
SSAVNQENERLMEEYERLASELLEWIRRTIPWLENRTPEKTMQAMQKKLE
DFRDYRRKHKPPKVQEKCQLEINFNTLQTKLRISNRPAFMPSEGKMVSDI
AGAWQRLEQAEKGYEEWLLNEIRRL----*
```

Figure S12A: Alignments used to build the models of nesprin-1 SRs

```
>P1;NESPRIN-1_SR38
sequence:NESPRIN-1_SR38
---LQRTRDYHDCMNVVEVFLEKFTTEWDNLARSDAESTAVHLEALKKLA
LALQERKYAIEDLKDQKQKMIEHLNLDDKELVKEQTSHLEQRWFQLEDLI
KRKIQVSVTNLEELNVVQS*
>P1;1HCI_SR4
structure:1HCI_SR4
HANERLRRQFAAQANAIGPWIQNKMEEIARSSIQITGALEDQMNQLKQYE
HNIINYKNNIDKLEGDHQLIQEALVF-DNKHTNYTMEHIRVGWELLLTTI
ARTINEVETQILTRD----*


>P1;NESPRIN-1_SR39
sequence:NESPRIN-1_SR39
VTNLEELNV-VQSRFQELMEWAEEQQPNIAEALKQSPPPDMAQNLLMDHL
AICSELEAKQMLLKSLIKDADRVMADLGLNERQVIQKALSDAQSHVNCLS
DLVGQRRKYLNKALSEKTQF------*
>P1;1QUU_SR3
structure:1QUU_SR3
-ETIDQLHLEFAKRAAPFNNWMEGAMEDLQDMFIVH-SIEEIQSLITAHE
QFKATLPEADGERQSIMAIQNEVEKVIQSYNIRISSSNPYSTVTMDELRT
KWDKVKQLVPIRDQSLQEELARQHAN*


>P1;NESPRIN-1_SR40
sequence:NESPRIN-1_SR40
LSEKTQFLMAVFQATSQIQQHERKIMFREHICLLPDDVSKQVKTCKSAQA
SLKTYQNEVTGLWAQGRELMKEVTEQEKSEVLGKLQELQSVYDSVLQKCS
HRLQELEKNLVS*
>P1;1S35_SR9
structure:1S35_SR9
--GFQEFQKDAKQAEAILSNQEYTLAHLE-PPDSLEAAEAGIRKFEDFLG
SMENNRDKVLSPVDSGNKLVAE-GNLYSDKIKEKVQLIEDRHRKNNEKAQ
EASVLLRDN---*
>P1;1U5P_SR15
structure:1U5P_SR15
ANKQQNFNTGIKDFDFWLSEVEALLASED-YGKDLASVNNLLKKHQLLEA
DISAHEDRLKDLNSQADSLMTS-SAFDTSQVKDKRETINGRFQRIKSMAA
ARRAKLNESHRL*
```

Figure S12A: Alignments used to build the models of nesprin-1 SRs

```
>P1;NESPRIN-1_SR41
sequence:NESPRIN-1_SR41
LVSRKHFKEDFDKACHWLKQADIVTFPEINLMNESTELHTQLAKYQNILE
QSPEYENLLLTLQRTGQTILPSLNEVDHSYLSEKLNALPRQFNVIVALAK
DKFYKVQEAILA*
>P1;1S35_SR9
structure:1S35_SR9
--GFQEFQKDAKQAEAILSNQEYTLAHL-EPPDSLEAAEAGIRKFEDFLG
SMENNRDKVLSPVDSGNKLVAE-GNLYSDKIKEKVQLIEDRHRKNNEKAQ
EASVLLRDN---*
>P1;1U5P_SR15
structure:1U5P_SR15
ANKQQNFNTGIKDFDFWLSEVEALLASE-DYGKDLASVNNLLKKHQLLEA
DISAHEDRLKDLNSQADSLMTS-SAFDTSQVKDKRETINGRFQRIKSMAA
ARRAKLNESHRL*


>P1;NESPRIN-1_SR42
sequence:NESPRIN-1_SR42
ILARKEYASLIELTTQSLSELEAQFLRMSKVPTDLAVEEALSLQDGCRAI
LDEVAGLGEAVDELNQKKEGFRSTG---QPWQPDKMLHLVTLYHRLKRQT
EQRVSLLEDT*
>P1;1S35_SR9
structure:1S35_SR9
--GFQEFQKDAKQAEAILSNQEYTLAHLEP---PDSLEAAEAGIRKFEDF
LGSMENNRDKVLSPVDSGNKLVAEGNLYSDKIKEKVQLIEDRHRKNNEKA
QEASVLLRDN*


>P1;NESPRIN-1_SR43
sequence:NESPRIN-1_SR43
TSAYQEHEKMCQQLERQLKSVKEEQSK--VNEETLPAEEKLKMYHSLAGS
LQDSGIVLKRVTIHLEDLAPHLDPLAYEKARHQIQSWQGELKLLTSAIGE
TVTECESRMVQSIDF*
>P1;1S35_SR9
structure:1S35_SR9
G--GFQEFQKDAKQAEAILSNQEYTLAHLEPPDSLEAAEAGIRKFEDFLGS
MENNRDKVLSPVDSGNKL-----VAEGNLYSDKIKEKVQLIEDRHRKNNE
KAQEASVLLRDN---*
>P1;1U5P_SR15
structure:1U5P_SR15
ANKQQNFNTGIKDFDFWLSEVEALLASEDYGKDLASVNNLLKKHQLLEAD
ISAHEDRLKDLNSQADSL-----MTSSAFDTSQVKDKRETINGRFQRIKS
MAAARRAKLNESHRL*
```

Figure S12A: Alignments used to build the models of nesprin-1 SRs

```
>P1;NESPRIN-1_SR44
sequence:NESPRIN-1_SR44
MVQSIDFQTEMSRSLDWLRRVKAELSGPVYLDLNLQDIQEEIRKIQIHQE
EVQSSLRIMNALSHKEKEKFTKAKELISADLEHSLAELSELDGDIQEALR
TRQATLTEIYSQ*
>P1;1S35_SR9
structure:1S35_SR9
--GFQEFQKDAKQAEAILSNQEYTLAHLEPPD-SLEAAEAGIRKFEDFLG
SMENNRDKVLSPVDSG-NKLVAEGNLYSDKIKEKVQLIEDRHRKNNEKAQ
EASVLLRDN---*
>P1;1U5P_SR15
structure:1U5P_SR15
ANKQQNFNTGIKDFDFWLSEVEALLASEDYGK-DLASVNNLLKKHQLLEA
DISAHEDRLKDLNSQA-DSLMTSSAFDTSQVKDKRETINGRFQRIKSMAA
ARRAKLNESHRL*
```

```
>P1;NESPRIN-1_SR45
sequence:NESPRIN-1_SR45
YSQCQRYYQVFQAANDWLEDAQEMLQLAGNGLDVESAEENLKSHMEFFST
EDQFHSNLEELHSLVATLDPLIKPTGKEDLEQKVASLELRSQRMSRDSGA
QVDLLQRC*
>P1;1S35_SR9
structure:1S35_SR9
--GFQEFQKDAKQAEAILSNQEYTLAHLEPPDSLEAAEAGIRKFEDFLGS
MENNRDKVLSPVDSGNKLVAE-GNLYSDKIKEKVQLIEDRHRKNNEKAQE
ASVLLRDN*
```

```
>P1;NESPRIN-1_SR46
sequence:NESPRIN-1_SR46
TAQWHDYQKAREEVIELMNDTEKKLSEFSLLKTSSSHEAEEKLSEHKALV
SVVNSFHEKIVALEEKASQLEKTGNDASKATLSRSMTTVWQRWTRLRAVA
QDQEKILEDA*
>P1;1S35_SR9
structure:1S35_SR9
--GFQEFQKDAKQAEAILSNQEYTLAHLEPPDS--LEAAEAGIRKFEDFL
GSMENNRDKVLSPVDSGNKLVAE-GNLYSDKIKEKVQLIEDRHRKNNEKA
QEASVLLRDN*
```

Figure S12A: Alignments used to build the models of nesprin-1 SRs

```
>P1;NESPRIN-1_SR47
sequence:NESPRIN-1_SR47
VDEWTGFNNKVKKATEMIDQLQDKLPGSSAEKASKAELLTLLEYHDTFVL
ELEQQQSALGMLRQQTLSMLQDGAAPTPGEEPPLMQEITAMQDRCLNMQE
KVKTNGKLVKQELKDREMV*
>P1;1S35_SR9
structure:1S35_SR9
--GFQEFQKDAKQAEAILSNQEYTL-AHLEPPDSLEAAEAGIRKFEDFLG
SMENNRDKVLSPVDSGNKLVAEGNL--------YSDKIKEKVQLIEDRHR
KNNEKAQEASVLLRDN---*
>P1;1U5P_SR15
structure:1U5P_SR15
ANKQQNFNTGIKDFDFWLSEVEALL-ASEDYGKDLASVNNLLKKHQLLEA
DISAHEDRLKDLNSQADSLMTSSAF--------DTSQVKDKRETINGRFQ
RIKSMAAARRAKLNESHRL*


>P1;NESPRIN-1_SR48
sequence:NESPRIN-1_SR48
KQELKDREMVETQINSVKCWVQETKEYLGNPTIE----IDAQLEELQILL
TEATNHRQNIEKMAEEQKEKYLGLYTILP-SELSLQLAEVALDLKIRDQI
QDKIKEVEQSKATSQ*
>P1;1HCI_SR4
structure:1HCI_SR4
HANERLRRQFAAQANAIGPWIQNKMEEIARSSIQITGALEDQMNQLKQYE
HNIINYKNNIDKLEGDHQLIQEALVFDNKHTNYTMEHIRVGWE-LLLTTI
ARTINEVETQILTRD*


>P1;NESPRIN-1_SR49
sequence:NESPRIN-1_SR49
ATSQELSRQIQKLAKDLTT-ILTKLKAKTDNVVQAKTDQKVLGEELDGCN
SKLMELDAAVQKFLEQNGQLGKPLA--KK-----IGKLTELHQQTIRQAE
NRLSKLNQAASHLE*
>P1;1HCI_SR4
structure:1HCI_SR4
HANERLRRQFAAQANAIGPWIQNKMEEIARSSIQITGALEDQMNQLKQYE
HNIINYKNNIDKLEGDHQLIQEALVFDNKHTNYTMEHIRVGWELLLTTIA
RTINEVETQILTRD*


>P1;NESPRIN-1_SR50
sequence:NESPRIN-1_SR50
NQAASHLEEYNEMLELILKWIEKAKVLAHGTIAWNSASQLREQYILHQTL
LEESKEIDSELEAMTEKLQYLTSVYCTEK--MSQQVAELGRETEELRQMI
KIRLQNLQDAAKDMK*
>P1;1HCI_SR4
structure:1HCI_SR4
HANERLRRQFAAQANAIGPWIQNKMEEIAR-SSIQITGALEDQMNQLKQY
EHNIINYKNNIDKLEGDHQLIQEALVFDNKHTNYTMEHIRVGWELLLTTI
ARTINEVETQILTRD*
```

Figure S12A: Alignments used to build the models of nesprin-1 SRs

```
>P1;NESPRIN-1_SR51
sequence:NESPRIN-1_SR51
AKDMKKFEAELKKLQAALEQAQATLTSPEVG--RLSLKEQLSHRQHLLSE
MESLKPKVQAVQLCQSALRIPEDVVASLPLCHAALRLQEEASRLQHTAIQ
QCNIMQEA*
>P1;1S35_SR9
structure:1S35_SR9
--GFQEFQKDAKQAEAILSNQEYTLAHLEPPDSLEAAEAGIRKFEDFLGS
MENNRDKVLSPVDSGNKLVAEGNLYSD-KIKEKVQLIEDRHRKNNEKAQE
ASVLLRDN*


>P1;NESPRIN-1_SR53
sequence:NESPRIN-1_SR53
LERQQKYQDSLQSISTKMEAIELKLSESP--EPGRSPESQMAEHQALMDE
ILMLQDEINELQSSLAEELVSESCEADPAEQLALQSTLTVLAERMSTIRM
KASGKRQLLEEK*
>P1;1S35_SR9
structure:1S35_SR9
--GFQEFQKDAKQAEAILSNQEYTLAHLEPPDSLEAAEAGIRKFEDFLGS
MENNRDKVLSPVDS-GNKLVA----EGNLYSDKIKEKVQLIEDRHRKNNE
KAQEASVLLRDN*


>P1;NESPRIN-1_SR55
sequence:NESPRIN-1_SR55
RQSIHLEQKLYDGVSATSTWLDDVEERL---FVATALLPEETETCLFNQE
ILAKDIKEMSEEMDKNKNLFSQAFPENGDNRDVIE---------DTLGCL
LGRLSLLDSVVNQRCHQMKERLQQILNF*
>P1;1QUU_SR3
structure:1QUU_SR3
ETIDQLHLEFAKRAAPFNNWMEGAMEDLQDMFIVHSI--EEIQSLITAHE
QFKATLPEADGERQSIMAIQNEVEKV--IQSYNIRISSSNPYSTVTMDEL
RTKWDKVKQLVPIRDQSLQEELARQHAN*


>P1;NESPRIN-1_SR56
sequence:NESPRIN-1_SR56
LNFQNDLKVLFTSLADNKYIILQKLANVFEQPVAEQIEAIQQAEDGLKEF
DAGIIELKRRGDELQVEQPSMQE------------LSKLQDMYDELMMII
GSRRSGLNQNLTLKS*
>P1;1HCI_SR4
structure:1HCI_SR4
-HANERLRRQFAAQANAIGPWIQNKMEEIARSSIQITGALEDQMNQLKQY
EHNIINYKNNIDKLEGDHQLIQEALVFDNKHTNYTMEHIRVGWELLLTTI
ARTINEVETQILTRD*
```

Figure S12A: Alignments used to build the models of nesprin-1 SRs

```
>P1;NESPRIN-1_SR57
sequence:NESPRIN-1_SR57
NQNLTLKSQYERALQDLADLLETGQEKMAGDQKIIVSSKEEIQQPLDKHK
EYFQGLESHMILTVTLFRKIISFAVQKETQF----HTELMAQASAVLKRA
HKRGVELEYILETWSH----------*
>P1;1QUU_SR3
structure:1QUU_SR3
ETIDQLHLEFAKRAAPFNNWMEGAMEDLQ--DMFIVHSIEEIQSLITAHE
QFKATLPEADGERQSIMAIQNEVEKVIQSYNIRISSSNPYSTVTMDELRT
KWDKVKQLVPIRDQSLQEELARQHAN*


>P1;NESPRIN-1_SR58
sequence:NESPRIN-1_SR58
---SHLDEDQQELSRQLEV-VESSIPSVGLVEENEDR-LIDRITLYQHLK
SSLNEYQPKLYQVLDDGKRLLISISCSDLESQ--LNQLGECWLSNTNKMS
KELHRLETIL----*
>P1;1HCI_SR4
structure:1HCI_SR4
HANERLRRQFAAQANAIGPWIQNKMEEIARSSIQITGALEDQMNQLKQYE
HNIINYKNNIDKLEGDHQLIQEALVFDNKHTNYTMEHIRVGWELLLTTIA
RTINEVETQILTRD*


>P1;NESPRIN-1_SR59
sequence:NESPRIN-1_SR59
KH-WTRYQSESADLIHWLQSAKDRLEFWTQQSVTVPQELEMVRDHLNAFL
EFSKEVDAQSSLKSSVLSTGNQLLRLKKVDTATLRSELSRIDSQWTDLLT
NIPAVQEKLHQLQMD*
>P1;1S35_SR9
structure:1S35_SR9
--GFQEFQKDAKQAEAILSNQEYTLAHLEPPD-----SLEAAEAGIRKFE
DFLGSMENNRDKVLSPVDSGNKLVAEGNLYSDKIKEKVQLIEDRHRKNNE
KAQEASVLLRDN---*
>P1;1U5P_SR15
structure:1U5P_SR15
ANKQQNFNTGIKDFDFWLSEVEALLASEDYGK-----DLASVNNLLKKHQ
LLEADISAHEDRLKDLNSQADSLMTSSAFDTSQVKDKRETINGRFQRIKS
MAAARRAKLNESHRL*


>P1;NESPRIN-1_SR60
sequence:NESPRIN-1_SR60
HQLQMDKLPSRHAISEVMSWTSLMENAIQKDEDNIKNSIGYKAIHEYLQK
YKGFKIDINCKQLTVDFVNQSVLQISSQDVESKRSDKTDFAEQLGAMNKS
WQILQGLVTEKIQLLEGLLESWS*
>P1;1HCI_SR4
structure:1HCI_SR4
HANERLRRQFAAQANAIGPWIQNKMEEIA--RSSIQITGALEDQMNQLKQ
YEHNII--NYKNNIDKLEGDHQLIQEALVFDN---KHTNYT--MEHIRVG
WELLLTTIARTINEVETQILTRD*
```

Figure S12A: Alignments used to build the models of nesprin-1 SRs

```
>P1;NESPRIN-1_SR61
sequence:NESPRIN-1_SR61
LESWSEYENNVQCLKTWFETQEKRLKQQHRIGDQASVQNALKDCQDLEDL
IKAKDKEVEKIEQNGLALIQTKKEDVSSIVMSTLRELGQTWANLDHMVGQ
LKILLKSVLDQ*
>P1;1S35_SR9
structure:1S35_SR9
--GFQEFQKDAKQAEAILSNQEYTLAHLEPPDSLEAAEAGIRKFEDFLGS
MENNRDKVLSPVDSGNKLVAEGNL-YSDKIKEKVQLIEDRHRKNNEKAQE
ASVLLRDN---*
>P1;1U5P_SR15
structure:1U5P_SR15
ANKQQNFNTGIKDFDFWLSEVEALLASEDYGKDLASVNNLLKKHQLLEAD
ISAHEDRLKDLNSQADSLMTSSAF-DTSQVKDKRETINGRFQRIKSMAAA
RRAKLNESHRL*


>P1;NESPRIN-1_SR62
sequence:NESPRIN-1_SR62
LDQWSSHKVAFDKINSYLMEARYSLSRFRLLTGSLEAVQVQVDNLQNLQD
DLEKQERSLQKFGSITNQLLKECHPPVTETLTNTLKEVNMRWNNLLEEIA
EQLQSSKALLQLWQR*
>P1;1S35_SR9
structure:1S35_SR9
--GFQEFQKDAKQAEAILSNQEYTLAHLE-PPDSLEAAEAGIRKFEDFLG
SMENNRDKVLSPVDSGNKLVAE-GNLYSDKIKEKVQLIEDRHRKNNEKAQ
EASVLLRDN------*
>P1;1U5P_SR15
structure:1U5P_SR15
ANKQQNFNTGIKDFDFWLSEVEALLASED-YGKDLASVNNLLKKHQLLEA
DISAHEDRLKDLNSQADSLMTS-SAFDTSQVKDKRETINGRFQRIKSMAA
ARRAKLNESHRL---*


>P1;NESPRIN-1_SR63
sequence:NESPRIN-1_SR63
LQLWQRYKDYSKQCASTVQQQEDRTNELLKAATNKDIADDEVATWIQDCN
DLLKGLGTVKDSLFVLHELGEQLKQQVDASAASAIQSDQLSLSQHLCALE
QALCKQQTSLQAGVLD*
>P1;1S35_SR9
structure:1S35_SR9
-----GFQEFQKDAKQAEAILSNQEYTLAHLEPPDSL--EAAEAGIRKFE
DFLGSMENNRDKVLSPVDSGNKLVAE-GNLYSDKIKEKVQLIEDRHRKNN
EKAQEASVLLRDN---*
>P1;1U5P_SR15
structure:1U5P_SR15
---ANKQQNFNTGIKDFDFWLSEVEALLASEDYGKDL--ASVNNLLKKHQ
LLEADISAHEDRLKDLNSQADSLMTS-SAFDTSQVKDKRETINGRFQRIK
SMAAARRAKLNESHRL*
```

Figure S12A: Alignments used to build the models of nesprin-1 SRs

```
>P1;NESPRIN-1_SR64
sequence:NESPRIN-1_SR64
VLDYETFAKSLEALEAWIVEAEEILQGQDPSHSSDLSTIQERMEELKGQM
LKFSSMAPDLDRLNELGYRLPL-----NDKEIKRMQNLNRHWSLISSQTT
ERFSKLQSF*
>P1;1S35_SR9
structure:1S35_SR9
--GFQEFQKDAKQAEAILSNQEYTLAHLE--PPDSLEAAEAGIRKFEDFL
GSMENNRDKVLSPVDSGNKLVAEGNLYSDKIKEKVQLIEDRHRKNNEKAQ
EASVLLRDN*


>P1;NESPRIN-1_SR65
sequence:NESPRIN-1_SR65
LLQHQTFLEKCETWMEFLVQTEQKLAVEISG-NYQHLLEQQRAHELFQAE
MFSRQQILHSIIIDGQRLLEQGQVDDRDEFNLKLTLLSNQWQGVIRRAQQ
RRGIIDSQ*
>P1;1S35_SR9
structure:1S35_SR9
--GFQEFQKDAKQAEAILSNQEYTLAHLEPPDSLEAAEAGIRKFEDFLGS
MENNRDKVLSPVDSGNKLVAEGNL-YSDKIKEKVQLIEDRHRKNNEKAQE
ASVLLRDN*


>P1;NESPRIN-1_SR66
sequence:NESPRIN-1_SR66
DSQIRQWQRYREMAEKLRKWLVEVSYLPMSGLGSVPIPLQ-QARTLFDEV
QFKEKVFLRQQGSYILTVEAGKQLLLSADSGAEAALQAELAEIQEKWKSA
SMRLEEQKKKLAFLLKDW-*
>P1;1HCI_SR4
structure:1HCI_SR4
HANERLRRQFAAQANAIGPWIQNKMEEIAR----SSIQITGALEDQMNQL
KQYEHNIINYKNNIDKLEGDHQLIQEAL-VFDNKHTNYTMEHIRVGWELL
LTTIARTINEVETQILTRD*


>P1;NESPRIN-1_SR67
sequence:NESPRIN-1_SR67
LKDWEKCEKGIADSLEKLRTFKKKLSQS-LPDHHEELHAEQMRCKELENA
VGSWTDDLTQLSLLKDTLSAYISADDISILNERVELLQRQWEELCHQLSL
RRQQIGERLNE*
>P1;1S35_SR9
structure:1S35_SR9
--GFQEFQKDAKQAEAILSNQEYTLAHLEPPDSLEAAEAGIRKFEDFLGS
MENNRDKVLSPVDSGNKLVAE-GNLYSDKIKEKVQLIEDRHRKNNEKAQE
ASVLLRDN---*
>P1;1U5P_SR15
structure:1U5P_SR15
ANKQQNFNTGIKDFDFWLSEVEALLASEDYGKDLASVNNLLKKHQLLEAD
ISAHEDRLKDLNSQADSLMTS-SAFDTSQVKDKRETINGRFQRIKSMAAA
RRAKLNESHRL*
```

Figure S12A: Alignments used to build the models of nesprin-1 SRs

```
>P1;NESPRIN-1_SR68
sequence:NESPRIN-1_SR68
VFSEKNKELCEWLTQMESKVSQN--GDILIEEMIEKLKKDYQEEIAIAQE
NKIQLQQMGERLAKASHESKASEIEYKLGKVNDRWQHLLDLIAARVKKLK
ETLVAV*
>P1;3EDV_SR15
structure:3EDV_SR15
KFYHDAKEIFGRIQDKHKKLPEELGRDQNTVETLQRMHTTFEHDIQALGT
QVRQLQEDAARLQAAYAGDKADDIQKRENEVLEAWKSLLDACESRRVRLV
DTGDKF*


>P1;NESPRIN-1_SR69
sequence:NESPRIN-1_SR69
LVAVQQLDKNMSSLRTWLAHIESELAKPIVYDSCNSEEIQRKLNEQQELQ
RDIEKHSTGVASVLNLCEVLLHDCDACATDAECDSIQQATRNLDRRWRNI
CAMSMERRLKIEET*
>P1;1S35_SR9
structure:1S35_SR9
--GFQEFQKDAKQAEAILSNQEYTLAHLEPPD--SLEAAEAGIRKFEDFL
GSMENNRDKVLSPVDSGNKLVAE-----GNLYSDKIKEKVQLIEDRHRKN
NEKAQEASVLLRDN*


>P1;NESPRIN-1_S70
sequence:NESPRIN-1_SR70
--------WRLWQKFLDDYSRFEDWLKSSERTAAFPSSSGVIYTVAKEEL
KKFEAFQR----QVHECLTQLELINKQYRRLARENRTDS-ACSLKQMVHE
GNQRWDNLQKRVTSILRRLKHF-----*
>P1;1HCI_SR4
structure:1HCI_SR4
-----HANERLRRQFAAQANAIGPWIQNKMEEIARS----S-IQITGALE
DQMNQLKQY-EHNIINYKNNIDKLEGDHQLIQEALVFD--NKHTNYTMEH
IRVGWELLLTTIARTINEVETQILTRD*
>P1;1HCI_SR1
structure:1HCI_SR1
SSAVNQENERLMEEYERLASELLEWIRRTIPWLENRTPEKT-MQAMQKKL
EDFRDYRRKHKPPKVQEKCQLEINFNTLQTKLRISNRPAFMPSEGKMVSD
IAGAWQRLEQAEKGYEEWLLNEIR-RL*
```

Figure S12A: Alignments used to build the models of nesprin-1 SRs

```
>P1;NESPRIN-1_S71
sequence:NESPRIN-1_SR71
---IGQ-----REEFETARDSILVWLTEMDLQLTNIE-HFSECDVQAKIK
QLKAFQQ----EISLNHNKIEQIIAQGEQLIEKSE-----PLDAAIIEEE
LDELRRYCQEVFGRVERYHKKLIRLPLP*
>P1;1HCI_SR4
structure:1HCI_SR4
-----HANERLRRQFAAQANAIGPWIQNKMEEIARS----SIQITGALED
QMNQLKQY-EHNIINYKNNIDKLEGDHQLIQEALVFD--NKHTNYTMEHI
RVGWELLLTTIARTINEVETQILTRD--*
>P1;1HCI_SR1
structure:1HCI_SR1
SSAVNQENERLMEEYERLASELLEWIRRTIPWLENRTPEKTMQAMQKKLE
DFRDYRRKHKPPKVQEKCQLEINFNTLQTKLRISNRPAFMPSEGKMVSDI
AGAWQRLEQAEKGYEEWLLNEIR-RL--*
```

```
>P1;NESPRIN-1_SR72
sequence:NESPRIN-1_SR72
S---SALESQIRQLGKALDD-SRFQIQQTENIIRSKTPTGPELDTSYKGYM
KLLGECSSSIDSVKRLEHKLKEEEESLPGFVNLHSTETQTAGVIDRWELL
QAQALSKELRMKQNLQKW-*
>P1;1HCI_SR4
structure:1HCI_SR4
HANERLRRQFAAQANAIGPWIQNKMEEIARSSIQITGALEDQMNQLKQYE
HNIINYKNNIDKLEGDHQLIQEALVFDNKHTNYT-----MEHIRVGWELL
LTTIARTINEVETQILTRD*
```

```
>P1;NESPRIN-1_SR73
sequence:NESPRIN-1_SR73
LQKWQQFNSDLNSIWAWLGDTEEELEQLQRLELSTDIQTIELQIKKLKEL
QKAVDHRKAIILSINLCSPEFTQADSKESRDLQDRLSQMNGRWDRVCSLL
EEWRGLLQDA*
>P1;1S35_SR9
structure:1S35_SR9
--GFQEFQKDAKQAEAILSNQEYTLAHL---EPPDSLEAAEAGIRKFEDF
LGSMENNRDKVLSPVDSGNKLVAEGNLYSDKIKEKVQLIEDRHRKNNEKA
QEASVLLRDN*
```

```
>P1;NESPRIN-1_SR74
sequence:NESPRIN-1_SR74
---LMQCQGFHEMSHGLLLMLENIDRRKNEIVPIDSNLDAE-ILQDHHKQ
LMQIKHELLESQLRVASLQDMSCQLLVNAEGTDCLEAKEKVHVIGNRLKL
LLKEVSRHIKELEKLLDVSS*
>P1;1HCI_SR4
structure:1HCI_SR4
HANERLRRQFAAQANAIGPWIQNKMEEIARS-----SIQITGALEDQMNQ
LKQYEHNIINYKNNIDKLEGDHQLIQEALV-FDNKHTNYTMEHIRVGWEL
LLTTIARTINEVETQILTRD*
```

Figure S12B: Alignments used to build the models of nesprin-2 SRs

A

```
>P1;NESPRIN2_SR1
sequence:NESPRIN2_SR1
--------------------GKVKDAMGWLTLQKEKLQKLL----KDSEN
DTYFKKYNSLLSFM-------ESFNEEKKSFLDVLSIKR-DLDELDKDHL
QLREAWDGLDHQINAWKIKLNYALPPP-*
>P1;1HCI_SR4
structure:1HCI_SR4
-HANERLRR--------QFAAQANAIGPWIQNKMEEIARS-----SIQIT
GALEDQMNQLKQYEHNIINYKNNIDKLEGDHQLIQEALVFDNKHTNYTME
HIRVGWELLLTTIARTINEVETQILTRD*
>P1;3EDV_SR14
structure:3EDV_SR14
--SHMRHRL-------FQLNREVDDLEQWIAEREVVAGSHELGQ-DYEHV
TMLQERFREFARDTGNI--GQERVDTVNHLADELINSGHSDAATIAEWKD
GLNEAWADLLELIDTRTQILAASYELH-*
>P1;1QUU_SR2
structure:1QUU_SR2
GSSNEIRRLERLEHLAEKFRQKASTHETWAYGKEQILLQKDYESASLTEV
RALLRKHEAFESDLAA---HQDRVEQIAAIAQELNELDYHDAVNVNDRCQ
KICDQWDRLGTLTQKRREALERMEKLL-*
```

```
>P1;NESPRIN2_SR2
sequence:NESPRIN2_SR2
---------AWKIKLNYALPPPLHQTEAWLQEVEELMDEDLSASQDHSQA
VTLIQEKMTLFKSLMDRFEHHSNILLTFENKDENHLP------------L
VPPNKLEEMKRRINNILEKKFILLLE--------*
>P1;1QUU_SR2
structure:1QUU_SR2
GSSNEIRRLERLEHLAEKFRQKASTHETWAYGKEQILLQKDYESASLTEV
RALLRKHEAFESDLAAHQDRVEQIAAIAQELNELDYHDAVNV-------N
DRCQKICDQWDRLGTLTQKRREALERMEKLL---*
>P1;1QUU_SR3
structure:1QUU_SR3
---------ETIDQLHLEFAKRAAPFNNWMEGAMEDL-QDMFIVHSIEEI
QSLITAHEQFKATLPEADGERQSIMAIQNEVEKVIQSYNIRISSSNPYST
VTMDELRTKWDKVKQLVPIRDQSLQEELARQHAN*
```

Figure S12B: Alignments used to build the models of nesprin-2 SRs

```
>P1;NESPRIN2_SR3
sequence:NESPRIN2_SR3
---------ILEKKFILLLEFHYYKCLVLGLVDEVKSKLDIWNIKYGS--
RESVELLLEDWHKFIEEKEFLARLDTSFQKCGEIYKNLA-----------
----GECQNINKQYMMVKSDVCMYRKNIYNVKSTLQKV*
>P1;1QUU_SR2
structure:1QUU_SR2
-GSSNEIRRLERLEHLAE--KFRQKAST-HETWAYGKEQILLQKDYESAS
LTEVRALLRKHEAFESDLAAHQDRVEQIAAIAQELNELDYHDAVNV----
---NDRCQKICDQWDRLGTLTQKRREALERMEKLL---*
>P1;1QUU_SR3
structure:1QUU_SR3
----------ETIDQLHL--EFAKRAAP-FNNWMEGAMEDL-QDMFIVHS
IEEIQSLITAHEQFKATLPEADGERQSIMAIQNEVEKVIQSYNIRISSSN
PYSTVTMDELRTKWDKVKQLVPIRDQSLQEELARQHAN*


>P1;NESPRIN2_SR4
sequence:NESPRIN2_SR4
----------------NVKSTLQKVLACWATYVENLRLLRA----CFEET
KKEEIKEVPFET-LAQW---NLEHATLNEAGNFLVEVSNDVVGSSISKEL
RRLNKRWRKLVSKTQLMNLPLMIKKQDQ-*
>P1;1HCI_SR4
structure:1HCI_SR4
-HANERLRR--------QFAAQANAIGPWIQNKMEEIARS-----SIQIT
GALEDQMNQLKQYEHNIINYKNNIDKLEGDHQLIQEA-LVFDNKHTNYTM
EHIRVGWELLLTTIARTINEVETQILTRD*
>P1;3EDV_SR14
structure:3EDV_SR14
SHMRHRL
--SHMRHRL-------FQLNREVDDLEQWIAEREVVAGSHELGQ-DYEHV
TMLQERFREFARDTGNI--GQERVDTVNHLADELINS-GHSDAATIAEWK
DGLNEAWADLLELIDTRTQILAASYELH-*
>P1;1QUU_SR2
structure:1QUU_SR2
GSSNEIRRLERLEHLAEKFRQKASTHETWAYGKEQILLQKDYESASLTEV
RALLRKHEAFESDLAA---HQDRVEQIAAIAQELNEL-DYHDAVNVNDRC
QKICDQWDRLGTLTQKRREALERMEKLL-*


>P1;NESPRIN2_SR5
sequence:NESPRIN2_SR5
-----VAKDVEKLIGQVEIWEAEAKSVLDQDDVD---TSMEESLKHLIAK
GSMFDELMARSEDMLQMDIQNISSQ-----------ESFQHVLTTGLQAK
IQEAKEKVQINVVKL-*
>P1;1HCI_SR4
structure:1HCI_SR4
HANERLRRQFAAQANAIGPWIQNKMEEIARSSI-QITGALEDQMNQLKQY
EHNIINY-KNNIDKLEGDHQLIQEALVFDNKHTNYTMEHIRVGWELLLTT
IARTINEVETQILTRD*
>P1;3EDV_SR14
structure:3EDV_SR14
SHMRHRLFQLNREVDDLEQWIAEREVVAGSHELGQDYEHVTMLQERFREF
ARDTGNIGQERVDTVNHLADELINSGHSDAATIAEWKDGLNEAWADLLEL
IDTRTQILAASYELH-*
```

Figure S12B: Alignments used to build the models of nesprin-2 SRs

```
>P1;NESPRIN2_SR6
sequence:NESPRIN2_SR6
DVSPDLDIRLKMEESQKELESYMMRAQQLLGQRE------SPGELISKHK
EALIISN---TKSLAKYLKAVEELKNNVTEDIKMSLEEKSRDVCAKWESL
HHELSLYVQQLKIDIEKG-*
>P1;1HCI_SR4
structure:1HCI_SR4
--HANERLRRQFAAQANAIGPWIQNKMEEIARSSI-QITGALEDQMNQLK
QYEHNIINY-KNNIDKLEGDHQLIQEA-LVFDNKHTNYTMEHIRVGWELL
LTTIARTINEVETQILTRD*
>P1;3EDV_SR14
structure:3EDV_SR14
--SHMRHRLFQLNREVDDLEQWIAEREVVAGSHELGQDYEHVTMLQERFR
EFARDTGNIGQERVDTVNHLADELINS-GHSDAATIAEWKDGLNEAWADL
LELIDTRTQILAASYELH-*
```

```
>P1;NESPRIN2_SR7
sequence:NESPRIN2_SR7
-----LSLYVQQLKIDIEKGKLSDNILKLEKQINKEKKLIR-----RGRT
KGLIKEHEACFSEEGCLYQLNHHMEVLRELCEELP--SQKSQQEVKRLLK
DYEQKIERLLKCASEI--------*
>P1;1QUU_SR2
structure:1QUU_SR2
GSSNEIRRLERLEHLAEKFRQKASTHETWAYGKEQILLQKDYESASLTEV
RALLRKHEAFESDLAAHQDRVEQIAAIAQELNELDYHDAVNVNDRCQKIC
DQWDRLGTLTQKRREALERMEKLL*
```

```
>P1;NESPRIN2_SR8
sequence:NESPRIN2_SR8
------------TYRDILEHHLQNNKFRITSDFSSEEDRSSSCLQAKLTD
LQVIKNETDARWKEFEIISLKLEN------HVNDIKKPFVIKERDTLKER
ERELQMTLNT-----*
>P1;3EDV_SR16
structure:3EDV_SR16
---------RFFSMVRDLMLWMEDVIRQIEAQEKPRDVSSVELLMNNHQG
IKAEIDARNDSFTTCIELGKSLLARKHYASEEIKEKLLQLTEKRKEMIDK
WEDRWEWLRL-----*
>P1;3FB2_SR15
structure:3FB2_SR15
HSHDSHDLQRFLSDFRDLMSWINGIRGLVSSDELAKDVTGAEALLERHQE
HRTEIDARAGTFQAFEQFGQQLLAHGHYASPEIKQKLDILDQERADLEKA
WVQRRMMLDQCLELQ*
```

```
>P1;NESPRIN2_SR9
sequence:NESPRIN2_SR9
-----------NIQDSIAKQIEICNR-------LEEPGNFVLK-ELHPFD
LHAMQNIILKYKTQFEG--------MNHRVQRSEDTLKALEDFLASLRTA
KLSAEP----*
>P1;1U5P_SR15
structure:1U5P_SR15
ANKQQNFNTGIKDFDFWLSEVEALLASEDYGKDLASVNNLLKKHQLLEAD
ISAHEDRLKDLNSQADSLMTSSAFDTSQVKDKRETINGRFQRIKSMAAAR
RAKLNESHRL*
```

Figure S12B: Alignments used to build the models of nesprin-2 SRs

```
>P1;NESPRIN2_SR10
sequence:NESPRIN2_SR10
RSEDTLKALEDFLASLRTAKL-SAEPVTDLSASDTQVAQENTLTVKNKEG
EIHL-MKDKAKHLDKCLKMLDMSFKDAE----RGDDTSCENLLDAFSI--
KLSETHGYGVQEEFTEEN*
>P1;1HCI_SR4
structure:1HCI_SR4
--HANERLRRQFAAQANAIGPWIQNKMEEIARSSI-QITGALEDQMNQLK
QYEHNIINY-KNNIDKLEGDHQLIQEALVFDNKHTNYTMEHIRVGWELLL
TTIARTINEVETQILTRD*
>P1;3EDV_SR14
structure:3EDV_SR14
--SHMRHRLFQLNREVDDLEQWIAEREVVAGSHELGQDYEHVTMLQERFR
EFARDTGNIGQERVDTVNHLADELINSGHSDAATIAEWKDGLNEAWADLL
ELIDTRTQILAASYELH-*
```

```
>P1;NESPRIN2_SR11
sequence:NESPRIN2_SR11
EFTEENKLLEACIFKNNELLKNIQDVQSQISKIGLKDPTVPAVKHRKKSL
IRLDKVLDEY-EEEKRHLQEMANSLPHFKDGREKTVNQQCQNTVVLWENT
KALVTECLEQCGRVLELLK-*
>P1;1HCI_SR4
structure:1HCI_SR4
--HANERLRRQFAAQANAIGPWIQNKMEEIARSSIQI--TGALEDQMNQL
KQYEHNIINY-KNNIDKLEGDHQLIQEALVFDNKHTNYTMEHIRVGWELL
LTTIARTINEVETQI-LTRD*
>P1;3EDV_SR14
structure:3EDV_SR14
--SHMRHRLFQLNREVDDLEQWIAEREVVAGSHELGQ-DYEHVTMLQERF
REFARDTGNIGQERVDTVNHLADELINSGHSDAATIAEWKDGLNEAWADL
LELIDTRTQILAASYELH--*
```

```
>P1;NESPRIN2_SR12
sequence:NESPRIN2_SR12
ECLEQCGRVLELLKQYQNFKSILTTLIQKEESVISLQASYMGKENLKKRI
AEIEIVKEE-FNEHLEVVDKINQVCKNLQFYLNKMKTFE-EPPFEKEANI
IVDRWLDINEKTEDYYENLGRALALWD*
>P1;1HCI_SR4
structure:1HCI_SR4
------HANERLRRQFAAQANAIGPWIQNKMEEIARS----SIQITGALE
DQMNQLKQY-EHNIINYKNNIDKLEGDHQLIQEALVFD--NKHTNYTMEH
IRVGWELLLTTIARTINEVETQILTRD*
>P1;1HCI_SR1
structure:1HCI_SR1
-SSAVNQENERLMEEYERLASELLEWIRRTIPWLENRTPEKTMQAMQKKL
EDFRDYRRKHKPPKVQEKCQLEINFNTLQTKLRISNRPAFMPSEGKMVSD
IAGAWQRLEQAEKGYEEWLLNEIR-RL*
```

Figure S12B: Alignments used to build the models of nesprin-2 SRs

```
>P1;NESPRIN2_SR13
sequence:NESPRIN2_SR13
YYENLGRALALWDKLFNLKNVIDEWTEKALQKMELHQLTEE-DRERLKEE
LQVHEQKTSEFSRRVAEIQFLLQSSEIPLELQ-----VMESSILNKMEHV
QKCLTGESNCHALSGSTAELR--*
>P1;1HCI_SR4
structure:1HCI_SR4
-----HANERLRRQFAAQANAIGPWIQNKMEEIARSSIQITGALEDQMNQ
LKQYEHNIINYKNNIDKLEGDHQLIQEALVFDNKHTNYTMEHIRVGWELL
LTTI--ARTINEV--ETQILTRD*


>P1;NESPRIN2_SR14
sequence:NESPRIN2_SR14
-------NCHALSGSTAELREDLDQAKTQIGMTESLLK-------ALSPS
DSL----EIF-TKLEEIQQQILQQKHSMILLENQ----IGCLTPELSELK
KQYESVSDLFNTKKSVLQDHFSKLLND*
>P1;1QUU_SR2
structure:1QUU_SR2
GSSNEIRRLERLEHLAEKFRQKASTHETWAYGKEQILLQKDYESASLTEV
RALLRKHEAFESDLAAHQDRVEQIAAIAQELNELDYHDAVNVNDRCQKIC
DQWDRLGTLTQKRREALERM-EKLL--*


>P1;NESPRIN2_SR15
sequence:NESPRIN2_SR15
VLQDHFSKLLNDQCKNFNDWFSNIKVNLK--ECFESSETKKSVEQKLQKL
SDFLTLEGRNSKIKQVDSVLKHVKKHLPKAHVKELISWLVGQEFELEKME
SICQARAKELEDSLQQL*
>P1;3EDU_SR14
structure:3EDU_SR14
----GSLFQLKRETDDLEQWISEKELVASSPEMGQDFDHVTLLRDKFRDF
ARETGAIG-QERVDNVNAFIERLIDA-GHSEAATIAEWKDGLNEMWADLL
ELIDTRMQLLAASYDLH*
>P1;3EDV_SR14
structure:3EDV_SR14
SHMRHRLFQLNREVDDLEQWIAEREVVAGSHELGQDYEHVTMLQERFREF
ARDTGNIG-QERVDTVNHLADELINS-GHSDAATIAEWKDGLNEAWADLL
ELIDTRTQILAASYELH*


>P1;NESPRIN2_SR16
sequence:NESPRIN2_SR16
-------EDSLQQLLRLQDDHRNLRKWLTNQEEKWKGMEEPGEKTELFCQ
ALAR-KREQFESVAQLNNSLKEYGFTEEEEIIMEATCLMDRYQTLLRQLS
EIEEEDKLLPTEDQS*
>P1;3EDU_SR14
structure:3EDU_SR14
----GSLFQLKRETDDLEQWISEKELVASSPEMGQDFDHVTLLRDKFRDF
ARETGAIGQERVDNVNAFIERLIDAGHSEAATIAEWKDGLNEMWADLLEL
IDTRMQLLAASYDLH*
>P1;3EDV_SR14
structure:3EDV_SR14
SHMRHRLFQLNREVDDLEQWIAEREVVAGSHELGQDYEHVTMLQERFREF
ARDTGNIGQERVDTVNHLADELINSGHSDAATIAEWKDGLNEAWADLLEL
IDTRTQILAASYELH*
```

Figure S12B: Alignments used to build the models of nesprin-2 SRs

```
>P1;NESPRIN2_SR17
sequence:NESPRIN2_SR17
KLLPTEDQSFNDLAHDVIHWIKEIKESLMVLNSSEGKMPLEERIQKIKEI
ILLKPEGDARIETIMKQAESSEAPLV------QKTLTDISNQWDNTLHLA
STYLSHQEKLLLEGEKYL*
>P1;1HCI_SR4
structure:1HCI_SR4
HANERLRRQFAAQANAIGPWIQNKMEEIARS-SIQITGALEDQMNQLKQY
EHNIINYKNNIDKLEGDHQLIQEALVFDNKHTNYTMEHIRVGWELLLTTI
ARTINEVETQILTRD---*


>P1;NESPRIN2_SR18
sequence:NESPRIN2_SR18
YLSHQEKLLLEG----EKYLQSKEDLRLMLIELKKKQEAGFALQHGLQEK
KAQLKIYKKFLKKAQD---LTSLLKELKSQGNYLLECTK-NPSFSEEPWL
EIKHLHESLLQQLQDSVQNLDGHVREHD*
>P1;1HCI_SR4
structure:1HCI_SR4
-HANERLRR--------QFAAQANAIGPWIQNKMEEIARS-----SIQIT
GALEDQMNQLKQYEHNIINYKNNIDKLEGDHQLIQEALVFDNKHTNYTME
HIRVGWELLLTTIARTINEVETQILTRD*
>P1;3EDV_SR14
structure:3EDV_SR14
--SHMRHRL-------FQLNREVDDLEQWIAEREVVAGSHELGQ-DYEHV
TMLQERFREFARDTGNI--GQERVDTVNHLADELINSGHSDAATIAEWKD
GLNEAWADLLELIDTRTQILAASYELH-*
>P1;1QUU_SR2
structure:1QUU_SR2
GSSNEIRRLERLEHLAEKFRQKASTHETWAYGKEQILLQKDYESASLTEV
RALLRKHEAFESDLAA---HQDRVEQIAAIAQELNELDYHDAVNVNDRCQ
KICDQWDRLGTLTQKRREALERMEKLL-*


>P1;NESPRIN2_SR19
sequence:NESPRIN2_SR19
-LDGHVREHDSYQVCVTDLNTTLDNFSKEFVSFSDKPVDQIAVEEKLQKL
QELENRLSLQDGTLKKILALAKSVKQNTSSVGQKIIKDDIKSLQCKQKDL
ENRLASAKQEMECCLNS-*
>P1;3EDV_SR16
structure:3EDV_SR16
----------RFFSMVRDLMLWMEDVIRQIEAQ-EKPRDVSSVELLMNNH
QGIKAEIDARNDSFTTCIELGKSLLARK-HYASEEIKEKLLQLTEKRKEM
IDKWEDRWEWLRL-----*
>P1;3FB2_SR15
structure:3FB2_SR15
HS-HDSHDLQRFLSDFRDLMSWINGIRGLVSSD-ELAKDVTGAEALLERH
QEHRTEIDARAGTFQAFEQFGQQLLAHGHY-ASPEIKQKLDILDQERADL
EKAWVQRRMMLDQCLELQ*
>P1;1S35_SR8
structure:1S35_SR8
--------EQAFLQDLDDFQAWLSITQKAVASE-DMPESLPEAEQLLQQH
AGIKDEIDGHQDSYQRVKESGEKVIQGQTDPEYLLLGQRLEGLDTGWDAL
GRMWESRSHTLAQCL---*
```

Figure S12B: Alignments used to build the models of nesprin-2 SRs

```
>P1;NESPRIN2_SR20
sequence:NESPRIN2_SR20
---DERKVNELQNQPLELDTMLRNEQ----LEEIEKLYTQLEAKKAAIKP
LEQTECLNKTETGALVLH----NIGYSAQHLDNLLQALITLKKNKESQYC
VLRDFQ*
>P1;1S35_SR8
structure:1S35_SR8
EQAFLQDLDDFQAWLSITQKAVASEDMPESLPEAEQLLQQHAGIKDEIDG
HQDSYQRVKESGEKVIQGQTDPEYLLLGQRLEGLDTGWDALGRMWESRSH
TLAQCL*


>P1;NESPRIN2_SR21
sequence:NESPRIN2_SR21
NKESQYCVLRDFQEYLAAVESSMKALLTDKESLKV-GPLD-SVTYLDKIK
KFIASIEKEKDSLGNLKIKWENLSNHVTDMDKKLLESQIKQLEHGWEQVE
QQIQKKYSQQVVEYDEFTT*
>P1;1HCI_SR4
structure:1HCI_SR4
--HANERLRRQFAAQANAIGPWIQNKMEEIARSSI-QITGALEDQMNQLK
QYEHNIINYKNNIDKLEGDHQLIQEAL-VFDNKHTNYTMEHIRVGWELLL
TTIARTINEVETQILTRD-*
>P1;3EDV_SR14
structure:3EDV_SR14
--SHMRHRLFQLNREVDDLEQWIAEREVVAGSHELGQDYEHVTMLQERFR
EFARDTGNIGQERVDTVNHLADELINSGHSDAATIAEWKDGLNEAWADLL
ELIDTRTQILAASYELH--*


>P1;NESPRIN2_SR22
sequence:NESPRIN2_SR22
QVVEYDEFTTLMNKVQDTEISLQQQQHLQLRLKSPEERAGNQSMIALTT
DLQATKHGFSVLKGQAELQMKRIWGEKEK------KNLEDGINNLKKQWE
TLEPLHLEAENQIKKCDIRN-*
>P1;1HCI_SR4
structure:1HCI_SR4
-HANERLRRQFAAQANAIGPWIQNKMEEIARS-SI-QITG---ALEDQMN
QLKQYEHNIINY-KNNIDKLEGDHQLIQEALVFDNKHTNYTMEHIRVGWE
LLLTTIARTINEVETQILTRD*
>P1;3EDV_SR14
structure:3EDV_SR14
-SHMRHRLFQLNREVDDLEQWIAEREVVAGSH-ELGQDYE---HVTMLQE
RFREFARDTGNIGQERVDTVNHLADELINSGHSDAATIAEWKDGLNEAWA
DLLELIDTRTQILAASYELH-*
```

Figure S12B: Alignments used to build the models of nesprin-2 SRs

```
>P1;NESPRIN2_SR23
sequence:NESPRIN2_SR23
PLHLEAENQIKKCDIRNKMKETILWAKNLLGELN-PSIPLLPDDILSQIR
KCKVTHDGILA-RQQSVESLAEEVKDKVPSLTTYEGSDLNNTLEDLRNQY
QMLVLKSTQRSQQLEFKLEER-*
>P1;1HCI_SR4
structure:1HCI_SR4
----HANERLRR-QFAAQANAIGPWIQNKMEEIARSSI-QITGALEDQMN
QLKQYEHNIIN-YKNNIDKLEGDHQLIQEA-LVFDNKHTNYTMEHIRVGW
ELLLTTIARTINEVETQILTRD*
>P1;3EDV_SR14
structure:3EDV_SR14
-----SHMRHRLFQLNREVDDLEQWIAEREVVAGSHELGQDYEHVTMLQE
RFREFARDTGNIGQERVDTVNHLADELINS-GHSDAATIAEWKDGLNEAW
ADLLELIDTRTQILAASYELH-*


>P1;NESPRIN2_SR24
sequence:NESPRIN2_SR24
---KLEERSNFFAIIRKFQLMVQESETLIIPRVETA---ATEAE---LKH
HHVTLEAS-QKELQEIDSGISTHLQELTNIYEELNVFE-RLFLEDQLKNL
KIRTNRIQRFIQNTCNEVEHKIKFCR-*
>P1;1HCI_SR4
structure:1HCI_SR4
-----HANERLRRQFAAQANAIGPWIQNKMEEIARS----SIQITGALED
QMNQLKQY-EHNIINYKNNIDKLEGDHQLIQEALVFD--NKHTNYTMEHI
RVGWELLLTTIARTINEVETQILT-RD*
>P1;1HCI_SR1
structure:1HCI_SR1
SSAVNQENERLMEEYERLASELLEWIRRTIPWLENRTPEKTMQAMQKKLE
DFRDYRRKHKPPKVQEKCQLEINFNTLQTKLRISNRPAFMPSEGKMVSDI
AGAWQRLEQAEKGYEEWLLNEIR--RL*


>P1;NESPRIN2_SR25
sequence:NESPRIN2_SR25
CNEVEHKIKFCRQFHEKTSALQEEADSIQRNELLLNQEVNKGVKEEIYNL
KDRLTAIKCCILQVLKLKKVFDYIGLN---------WDFSQL----DQLQ
TQVFEKEKELEEKIKQLD*
>P1;1HCI_SR4
structure:1HCI_SR4
HANERLRRQFAAQANAIGPWIQNKMEEIAR----SSIQITGALEDQMNQL
KQYEHNIINYKNNIDKLEGDHQLIQEALVFDNKHTNYTMEHIRVGWELLL
TTIARTINEVETQILTRD*
```

Figure S12B: Alignments used to build the models of nesprin-2 SRs

```
>P1;NESPRIN2_SR26
sequence:NESPRIN2_SR26
QLDTFEEEHGKYQALLSKMRA-IDLQIKKMTEVVLKAPDSSPESRRLNAQ
ILSQRIEKAKCLCDEIIKKLNENKTFDDSFKEKEILQ-IKLNAEENDKLY
KVLQNMVLELSPKELDEK*
>P1;1HCI_SR4
structure:1HCI_SR4
--HANERLRRQFAAQANAIGPWIQNKMEEIARSSI-QITGALEDQMNQLK
QYEHNIINY-KNNIDKLEGDHQLIQEALVFDNKHTNYTMEHIRVGWELLL
TTIARTINEVETQILTRD*
>P1;3EDV_SR14
structure:3EDV_SR14
--SHMRHRLFQLNREVDDLEQWIAEREVVAGSHELGQDYEHVTMLQERFR
EFARDTGNIGQERVDTVNHLADELINSGHSDAATIAEWKDGLNEAWADLL
ELIDTRTQILAASYELH-*


>P1;NESPRIN2_SR27
sequence:NESPRIN2_SR27
ELSPKELDEKNCQDKLETSLHVLNQIKSQLQQPLLINLEIKHIQNEKDNC
EAFQEQVWA-EMCSIKAVTAIEKQREENSS-EASDVETKLREFEDLQMQL
NTSIDLRTNVLNDAYENLT*
>P1;1HCI_SR4
structure:1HCI_SR4
--HANERLRRQFAAQANAIGPWIQNKMEEIAR-SSI-QITGALEDQMNQL
KQYEHNIINY-KNNIDKLEGDHQLIQEALVFDNKHTNYTMEHIRVGWELL
LTTIARTINEVETQILTRD*
>P1;3EDV_SR14
structure:3EDV_SR14
--SHMRHRLFQLNREVDDLEQWIAEREVVAGS-HELGQDYEHVTMLQERF
REFARDTGNIGQERVDTVNHLADELINSGHSDAATIAEWKDGLNEAWADL
LELIDTRTQILAASYELH-*

>P1;NESPRIN2_SR28
sequence:NESPRIN2_SR28
NVLNDAYENLTRYKEAVTRAVESITSLEAIIIPYRVDV------GNPEES
LEMPLRKQEELESTVAHIQDLTEKLGMISSPEAKLQLQY-----------
--TLQELVSKNSAMKEAFKAQETEAERYLENYKCY*
>P1;1QUU_SR2
structure:1QUU_SR2
-GSSNEIRRLERLEHLAEKFRQKASTHETWAYGKEQILLQKDYESASLTE
VRALLRKHEAFESDLAAHQDRVEQIAAIAQELNELDYHDAVNV-------
NDRCQKICDQWDRLGTLTQKRREALERMEKLL---*
>P1;1QUU_SR3
structure:1QUU_SR3
----------ETIDQLHLEFAKRAAPFNNWMEGAMEDL-QDMFIVHSIEE
IQSLITAHEQFKATLPEADGERQSIMAIQNEVEKVIQSYNIRISSSNPYS
TVTMDELRTKWDKVKQLVPIRDQSLQEELARQHAN*
```

Figure S12B: Alignments used to build the models of nesprin-2 SRs

```
>P1;NESPRIN2_SR29
sequence:NESPRIN2_SR29
EAERYLENYKCYRKMEEDIYTNLSKMETVLGQSMSSLPLSYREALERLEQ
SKALVSNLIST-KEELMKLRQILRLLRLRCTENDGICLLKIVSALWEKWL
SLLEAAKEWEMWCEELKQEWK*
>P1;1HCI_SR4
structure:1HCI_SR4
--HANERLRRQFAAQANAIGPWIQNKMEEIARSSI-Q--ITGALEDQMNQ
LKQYEHNIINY-KNNIDKLEGDHQLIQEA-LVFDNKHTNYTMEHIRVGWE
LLLTTIARTINEVETQILTRD*
>P1;3EDV_SR14
structure:3EDV_SR14
--SHMRHRLFQLNREVDDLEQWIAEREVVAGSHELGQ--DYEHVTMLQER
FREFARDTGNIGQERVDTVNHLADELINS-GHSDAATIAEWKDGLNEAWA
DLLELIDTRTQILAASYELH-*


>P1;NESPRIN2_SR30
sequence:NESPRIN2_SR30
-ELKQEWKFVSEEIEREAIILDNLQEELPEISKTKEAATTEELSELLDCL
CQYGENVEKQQLLLTLLLQRIRSIQNVPESSGAVETVPAFQEITSMKERC
NKLLQKVQKNKELVQTEIQERHSFTKE*
>P1;1HCI_SR4
structure:1HCI_SR4
HANERLRRQFAAQANAIGPWIQNKMEEIARSSI-QITGALEDQMNQLKQY
EHNIINY-KNN--IDKLEGDHQLIQEALVFDNK----HTNYTMEHIRVGW
ELLLTTIARTINEVETQILTRD-----*
>P1;3EDV_SR14
structure:3EDV_SR14
SHMRHRLFQLNREVDDLEQWIAEREVVAGSHELGQDYEHVTMLQERFREF
ARDTGNIGQER--VDTVNHLADELINSGHSDAA----TIAEWKDGLNEAW
ADLLELIDTRTQILAASYELH------*


>P1;NESPRIN2_SR31
sequence:NESPRIN2_SR31
NKELVQTEIQERHSFTKEIIALKNFFQQTTTSFQNM-AFQDHPEKSEQFE
ELQSILKKGKLTFENIMEKLRIKYSEMY--TIVPAE--IESQVEECRKAL
EDIDEKISNEVLKSSPSY-------*
>P1;1HCI_SR1
structure:1HCI_SR1
SSAVNQENERLMEEYERLASELLEWIRRTIPWLENRTPEKTMQAMQKKLE
DFRDYRRKHKPPKVQEKCQLEINFNTLQTKLRISNRPAFMPSEGKMVSDI
AGAWQRLEQAEKGYEEWLLNEIRRL*
```

Figure S12B: Alignments used to build the models of nesprin-2 SRs

```
>P1;NESPRIN2_SR32
sequence:NESPRIN2_SR32
-LKSSPSYAMRRKIEEINNGLHNVEKMLQQKS-----KNIEKAQEIQKKM
WDELDLW-HSKLNELDSEVQDIVEQDPGQAQEWMDNLMIPFQQYQQVSQR
AECRTSQLNKATVKME*
>P1;1HCI_SR4
structure:1HCI_SR4
HANERLRRQFAAQANAIGPWIQNKMEEIARSSI-QITGALEDQMNQLKQY
EHNIINY-KNNIDKLEGDHQLIQEALVFDNKHTNYTMEHIRVGWELLLTT
IARTINEVETQILTRD*
>P1;3EDV_SR14
structure:3EDV_SR14
SHMRHRLFQLNREVDDLEQWIAEREVVAGSHELGQDYEHVTMLQERFREF
ARDTGNIGQERVDTVNHLADELINSGHSDAATIAEWKDGLNEAWADLLEL
IDTRTQILAASYELH-*
```

```
>P1;NESPRIN2_SR33
sequence:NESPRIN2_SR33
SQLNKATVKMEEYSDLLKSTEAWIENTSHLLANP---ADYDSLRTLSHHA
STVQMALEDSEQKHNLLHSIFMDLEDLSIIFETDE--LTQSIQELSNQVT
ALQQKIMESLPQIQRMADDVV*
>P1;1HCI_SR4
structure:1HCI_SR4
---HANERLRRQFAAQANAIGPWIQNKMEEI-AR---SSIQITGALEDQM
NQLKQYEHNIINYKNNIDKLEGDHQLIQEALVFDNKHTNYTMEHIRVGWE
LLLTTIARTINEVETQILTRD*
>P1;3EDV_SR14
structure:3EDV_SR14
---SHMRHRLFQLNREVDDLEQWIAEREVVAGSHELGQDYEHVTMLQERF
REFARDTGNIGQ--ERVDTVNHLADELINSGHSDAATIAEWKDGLNEAWA
DLLELIDTRTQILAASYELH-*
```

```
>P1;NESPRIN2_SR34
sequence:NESPRIN2_SR34
-SLPQIQRM-ADDVVAIESEVKSMEKRVSKIKTILLSKEIFDFSPEEHLK
HGEVILENIRPMKKTIAEIVSYQVELRL--PQTGMKPLPVFQRTNQLLQD
IKLLENVTQEQN----*
>P1;1HCI_SR4
structure:1HCI_SR4
HANERLRRQFAAQANAIGPWIQNKMEEIARSSIQIT-GALED-QMNQLKQ
YEHNIINYKNNIDKLEGDHQLIQEALVFDNKHTNYTMEHIRVGWELLLTT
IARTINEVETQILTRD*
```

Figure S12B: Alignments used to build the models of nesprin-2 SRs

```
>P1;NESPRIN2_SR35
sequence:NESPRIN2_SR35
--------QNELLKVVIKQTNEQDEEIENLKQILNNYSAQFSLEHMSPDQ
ADKLPQLQGEIERM-EKQILSLNQRKEDL--------LVDLKATVLNLHQ
HLKQEQEGVERDRLPAV*
>P1;1HCI_SR4
structure:1HCI_SR4
HANERLRRQFAAQANAIGPWIQNKMEEIARSSI-QITGAL-EDQMNQLKQ
YEHNIINY-KNNIDKLEGDHQLIQEALVFDNKHTNYTMEHIRVGWELLLT
TIARTINEVETQILTRD*
>P1;3EDV_SR14
structure:3EDV_SR14
SHMRHRLFQLNREVDDLEQWIAEREVVAGSHELGQDYEHV-TMLQERFRE
FARDTGNIGQERVDTVNHLADELINSGHSDAATIAEWKDGLNEAWADLLE
LIDTRTQILAASYELH-*


>P1;NESPRIN2_SR36
sequence:NESPRIN2_SR36
--KTRPEPTEVLHACKTQVAELELWLQQANVAVEPET--LNADMQQVLEQ
QLVGCQA----MLTEIEHKVAFLLETCKDQGLGDNGA-----TQHEAEAL
SLKLKTVKCNLQKVQMMLQEKHSEDQHPTILKK*
>P1;1HCI_SR4
structure:1HCI_SR4
-----HANERLRRQFAAQANAIGPWIQNKMEEIARS----SIQITGALED
QMNQLKQY-EHNIINYKNNIDKLEGDHQLIQEALVFD--NKHTNYTMEHI
RVGWELLLTTIARTINEVETQILTRD-------*
>P1;1HCI_SR1
structure:1HCI_SR1
SSAVNQENERLMEEYERLASELLEWIRRTIPWLENRTPEKTMQAMQKKLE
DFRDYRRKHKPPKVQEKCQLEINFNTLQTKLRISNRPAFMPSEGKMVSDI
AGAWQRLEQAEKGYEEWLLNEIR-RL-------*


>P1;NESPRIN2_SR37
sequence:NESPRIN2_SR37
NMTEEAYINLDKKLFELFLTLSQCLSSVEEMLEMPRL--YREDGSGQQVH
YETLALELKKLYLALSDKKGDLLKAMTWPGE-NTNLLLECFDNLQVCLEH
TQAAAVCRSKSLKAGLDYNR*
>P1;1HCI_SR4
structure:1HCI_SR4
----HANERLRRQFAAQANAIGPWIQNKMEEIARSSI-QITGALEDQMNQ
LKQYEHNIINY-KNNIDKLEGDHQLIQEALVFDNKHTNYTMEHIRVGWEL
LLTTIARTINEVETQILTRD*
>P1;3EDV_SR14
structure:3EDV_SR14
----SHMRHRLFQLNREVDDLEQWIAEREVVAGSHELGQDYEHVTMLQER
FREFARDTGNIGQERVDTVNHLADELINSGHSDAATIAEWKDGLNEAWAD
LLELIDTRTQILAASYELH-*
```

Figure S12B: Alignments used to build the models of nesprin-2 SRs

```
>P1;NESPRIN2_SR38
sequence:NESPRIN2_SR38
----NEIKRLYHQLIKSKTSLQQSLNEIS-------GQSVAEQLQKADAY
TVELEN-AESRVAKLRDEGERLHLP-YALLQEVYKLEDVLDSMWGMLRAR
YTELSSSFVTE----*
>P1;3EDU_SR14
structure:3EDU_SR14
----GSLFQLKRETDDLEQWISEKELVASSPEMGQDFDHVTLLRDKFRDF
ARETGAIGQERVDNVNAFIERLIDAGHSEAATIAEWKDGLNEMWADLLEL
IDTRMQLLAASYDLH*
>P1;3EDV_SR14
structure:3EDV_SR14
SHMRHRLFQLNREVDDLEQWIAEREVVAGSHELGQDYEHVTMLQERFREF
ARDTGNIGQERVDTVNHLADELINSGHSDAATIAEWKDGLNEAWADLLEL
IDTRTQILAASYELH*

>P1;NESPRIN2_SR39
sequence:NESPRIN2_SR39
TELSSSFVTESQQDALLQGMVELVKIGKEKLAHGHLKQTKSKVALQAQIE
NHKVFFQKLVADMLLIQAYSAKILPSL------LQIRETFWAEQVTEVKI
LEEKPRQCGMKLQSLLQKW*
>P1;1U5P_SR15
structure:1U5P_SR15
------ANKQQNFNTGIKDFDFWLSEVEALLASED--YGKDLASVNNLLK
KHQLLEADISAHEDRLKDLNSQADSLMTSSAFDTSQVKDKRETINGRFQR
IKSMAAARRAKLNESHRL-*
>P1;1U5P_SR16
structure:1U5P_SR16
----------HQFFRDMDDEESWIKEKKLLVSSED--YGRDLTGVQNLRK
KHKRLEAELAAHEPAIQGVLDTGKKLSDDNTIGKEEIQQRLAQFVDHWKE
LKQLAAARGQRLE------*
>P1;1CUN_SR16
structure:1CUN_SR16
--------MVHQFFRDMDDEESWIKEKKLLVSSED--YGRDLTGVQNLRK
KHKRLEAELAAHEPAIQSVLDTGKKLSDDNTIGKEEIQQRLAQFVDHWKE
LKQLAAARGQRLEESLEYQ*

>P1;NESPRIN2_SR40
sequence:NESPRIN2_SR40
QKWEEFDENYASLEKDLEI-LISTLPSVSLVEETEER-LVERISFYQQIK
RNIGGKHARLYQTLNEGKQLVASVSCPELEGQ--IAKLEEQWLSLNKKID
HELHRLQALL----*
>P1;1HCI_SR4
structure:1HCI_SR4
HANERLRRQFAAQANAIGPWIQNKMEEIARSSIQITGALEDQMNQLKQYE
HNIINYKNNIDKLEGDHQLIQEALVFDNKHTNYTMEHIRVGWELLLTTIA
RTINEVETQILTRD*
```

Figure S12B: Alignments used to build the models of nesprin-2 SRs

```
>P1;NESPRIN2_SR41
sequence:NESPRIN2_SR41
----KHLLSYNRDSDQLTKWLESSQHTLNYWKEQSLNVSQDLDTIRSNIN
NFFEFSKEVDE-KSSLKTAVISIGNQLLHLKETDTATLRASLAQFEQKWT
MLITQLPDIQEKLHQLQMEK*
>P1;3EDU_SR14
structure:3EDU_SR14
----GSLFQLKRETDDLEQWISEKELVASS-----PEMGQDFDHVTLLRD
KFRDFARETGAIGQERVDNVNAFIERLIDAGHSEAATIAEWKDGLNEMWA
DLLELIDTRMQLLAASYDLH*
>P1;3EDV_SR14
structure:3EDV_SR14
SHMRHRLFQLNREVDDLEQWIAEREVVAGS-----HELGQDYEHVTMLQE
RFREFARDTGNIGQERVDTVNHLADELINSGHSDAATIAEWKDGLNEAWA
DLLELIDTRTQILAASYELH*


>P1;NESPRIN2_SR42
sequence:NESPRIN2_SR42
---------HQLQMEKLPSRKAITEMISWMNNVEHQTSDEDSVHSPSSAS
QVKHLLQKHKEFRMEMDYKQWIVDFVNQSLLQLSTCDVESKRYERTE---
FAEHLGEMNRQWHRVHGMLNRKIQHLDQLLESITE-*
>P1;1QUU_SR2
structure:1QUU_SR2
GSSNEIRRLERLEHLAEKFRQKASTHETWAYGKEQILLQKDYESAS--LT
EVRALLRKHEAFESDLAAHQDRVEQIAAIAQELNELDYHDAVNV------
-NDRCQKICDQWDRLGTLTQKRREALERMEKLL---*
>P1;1QUU_SR3
structure:1QUU_SR3
---------ETIDQLHLEFAKRAAPFNNWMEGAMEDL-QDMFIVHS--IE
EIQSLITAHEQFKATLPEADGERQSIMAIQNEVEKVIQSYNIRISSSNPY
STVTMDELRTKWDKVKQLVPIRDQSLQEELARQHAN*


>P1;NESPRIN2_SR43
sequence:NESPRIN2_SR43
LDQLLESIT--ESENKIQILNNWLEAQEERLKTLQKPESVISVQKLLLDC
QDIENQLAIKSKALDELKQSYLTLESGAVP-LLEDTASRIDELFQKRSSV
LTQVNQLKTSMQSVLQEW*
>P1;3EDV_SR16
structure:3EDV_SR16
-----------RFFSMVRDLMLWMEDVIRQIEAQEKPRDVSSVELLMNNH
QGIKAEIDARNDSFTTCIELGKSLLARKHY-ASEEIKEKLLQLTEKRKEM
IDKWEDRWEWLRL-----*
>P1;3FB2_SR15
structure:3FB2_SR15
--HSHDSHDLQRFLSDFRDLMSWINGIRGLVSSDELAKDVTGAEALLERH
QEHRTEIDARAGTFQAFEQFGQQLLAHGHY-ASPEIKQKLDILDQERADL
EKAWVQRRMMLDQCLELQ*
>P1;1S35_SR8
structure:1S35_SR8
---------EQAFLQDLDDFQAWLSITQKAVASEDMPESLPEAEQLLQQH
AGIKDEIDGHQDSYQRVKESGEKVIQGQTDPEYLLLGQRLEGLDTGWDAL
GRMWESRSHTLAQCL---*
```

Figure S12B: Alignments used to build the models of nesprin-2 SRs

```
>P1;NESPRIN2_SR44
sequence:NESPRIN2_SR44
QLKTSMQSVLQEWKIYDQLYDEVNMMTIRFWYCMEHSKPVVLSLETLRCQ
VENLQSLQDEAES-SEGSWEKLQEVIGKLKGLCPSVAEIIEEKCQNTHKR
WTQVNQAIADQLQKAQSLLQLWK*
>P1;1HCI_SR4
structure:1HCI_SR4
------HANERLRRQFAAQANAIGPWIQNKMEEIARSS-I-QITGALEDQ
MNQLKQYEHNIINY-KNNIDKLEGDHQLIQEALVFDNKHTNYTMEHIRVG
WELLLTTIARTINEVETQILTRD*
>P1;3EDV_SR14
structure:3EDV_SR14
------SHMRHRLFQLNREVDDLEQWIAEREVVAGSHE-LGQDYEHVTML
QERFREFARDTGNIGQERVDTVNHLADELINSGHSDAATIAEWKDGLNEA
WADLLELIDTRTQILAASYELH-*


>P1;NESPRIN2_SR45
sequence:NESPRIN2_SR45
--NAHGEAAARLKQQE-AKFQQLANISMSGNNL---AEILPPALQDIKEL
QHDVQKT-KEAFLQNSSVLDRLPQPAESSTHMLLPGPLHSLQRA-AYLEK
MLLVKANEFEF------*
>P1;1HCI_SR4
structure:1HCI_SR4
HANERLRRQFAAQANAIGPWIQNKMEEIARSSI-QITGALEDQMNQLKQY
EHNIINY-KNNIDKLEGDHQLIQEALVFDNKHT-NYTMEHIRVGWELLLT
TIARTINEVETQILTRD*
>P1;3EDV_SR14
structure:3EDV_SR14
SHMRHRLFQLNREVDDLEQWIAEREVVAGSHELGQDYEHVTMLQERFREF
ARDTGNIGQERVDTVNHLADELINSGHSDAATI-AEWKDGLNEAWADLLE
LIDTRTQILAASYELH-*


>P1;NESPRIN2_SR46
sequence:NESPRIN2_SR46
-----KANEFEFVLSQFKDFGVRLESLKGLIMHEEENLDRLHQ-QEKENP
DSFLNHVLALTAQSPDIEHLNEVSLKLPLS-----DVAVKTLQNMNRQWI
RATATALERCSELQGIGLNEK*
>P1;1HCI_SR4
structure:1HCI_SR4
-----HANERLRRQFAAQANAIGPWIQNKMEEIARSSI-QITGALEDQMN
QLKQYEHNIINY-KNNIDKLEGDHQLIQEALVFDNKHTNYTMEHIRVGWE
LLLTTIARTINEVETQILTRD*
>P1;3EDV_SR14
structure:3EDV_SR14
-----SHMRHRLFQLNREVDDLEQWIAEREVVAGSHELGQDYEHVTMLQE
RFREFARDTGNIGQERVDTVNHLADELINSGHSDAATIAEWKDGLNEAWA
DLLELIDTRTQILAASYELH-*
```

Figure S12B: Alignments used to build the models of nesprin-2 SRs

```
>P1;NESPRIN2_SR47
sequence:NESPRIN2_SR47
-QGIGLNEKFLYCCEKWIQLLEKIEEALK-VDVANSLPELLEQQKTYKML
EAEVSINQAIADSYVTQSLQLLDTTEIENRPEFITEFSKLTDRWQNAVQG
VRQRKGDVD-------*
>P1;1HCI_SR4
structure:1HCI_SR4
HANERLRRQFAAQANAIGPWIQNKMEEIARSSI-QITGALEDQMNQLKQY
EHNIINY-KNNIDKLEGDHQLIQEALVFDNKHTNYTMEHIRVGWELLLTT
IARTINEVETQILTRD*
>P1;3EDV_SR14
structure:3EDV_SR14
SHMRHRLFQLNREVDDLEQWIAEREVVAGSHELGQDYEHVTMLQERFREF
ARDTGNIGQERVDTVNHLADELINSGHSDAATIAEWKDGLNEAWADLLEL
IDTRTQILAASYELH-*


>P1;NESPRIN2_SR48
sequence:NESPRIN2_SR48
----------FTTSVENLFRFLTDTSHLLSAVRGQERFSLYQTRSLIHEL
KNKEIHFQRRRTTCALTLEAGEKLLLTTDLKTKESVGRRISQLQDSWKDM
EPQLAEMIKQFQSTVETW*
>P1;3EDV_SR16
structure:3EDV_SR16
---------RFFSMVRDLMLWMEDVIRQIEAQ--EKPRDVSSVELLMNNH
QGIKAEIDARNDSFTTCIELGKSLLARKHY-ASEEIKEKLLQLTEKRKEM
IDKWEDRWEWLRL-----*
>P1;3FB2_SR15
structure:3FB2_SR15
HSHDSHDLQRFLSDFRDLMSWINGIRGLVSSD--ELAKDVTGAEALLERH
QEHRTEIDARAGTFQAFEQFGQQLLAHGHY-ASPEIKQKLDILDQERADL
EKAWVQRRMMLDQCLELQ*
>P1;1S35_SR8
structure:1S35_SR8
-------EQAFLQDLDDFQAWLSITQKAVASE--DMPESLPEAEQLLQQH
AGIKDEIDGHQDSYQRVKESGEKVIQGQTDPEYLLLGQRLEGLDTGWDAL
GRMWESRSHTLAQCL---*


>P1;NESPRIN2_SR49
sequence:NESPRIN2_SR49
EMIKQFQSTVETWDQCEKKIKE-LKSRLQVLKAQSEDPLPELHEDLHNEK
ELIKELEQSLASWTQNLKELQTMKADLTRHVLVEDVMVLKEQIEHLHRQW
EDLCLRVAIRKQEIEDRLNTWV*
>P1;1HCI_SR4
structure:1HCI_SR4
---HANERLRRQFAAQANAIGPWIQNKMEEIARSS----IQITGALEDQM
NQLKQYEHNIINYKNNIDKLEGDHQLIQEAL-VFDNKHTNYTMEHIRVGW
ELLLTTIARTINEVETQILTRD*
>P1;3EDV_SR14
structure:3EDV_SR14
---SHMRHRLFQLNREVDDLEQWIAEREVVAGSHELGQDYEHVTMLQERF
REFARDTGNIGQ--ERVDTVNHLADELINSG-HSDAATIAEWKDGLNEAW
ADLLELIDTRTQILAASYELH-*
```

Figure S12B: Alignments used to build the models of nesprin-2 SRs

```
>P1;NESPRIN2_SR50
sequence:NESPRIN2_SR50
RKQEIEDRLNTWVVFNEKNKELCAWLVQMENKVLQTADI-SIEEM--IEK
LQKDCME----EINLFSENKLQLKQMGDQLIKASNKSR-AAEIDDKLNKI
NDRWQHLFDVIGSRVKKLKETFAFIQ*
>P1;1HCI_SR4
structure:1HCI_SR4
-----HANERLRRQFAAQANAIGPWIQNKMEEIARS----SIQITGALED
QMNQLKQY-EHNIINYKNNIDKLEGDHQLIQEALVFD--NKHTNYTMEHI
RVGWELLLTTIARTINEVETQILTRD*
>P1;1HCI_SR1
structure:1HCI_SR1
SSAVNQENERLMEEYERLASELLEWIRRTIPWLENRTPEKTMQAMQKKLE
DFRDYRRKHKPPKVQEKCQLEINFNTLQTKLRISNRPAFMPSEGKMVSDI
AGAWQRLEQAEKGYEEWLLNEIR-RL*


>P1;NESPRIN2_SR51
sequence:NESPRIN2_SR51
KETFAFIQQLDKNMSNLRTWLARIESELSKPVVYDVCDDQEIQKRLAEQQ
DLQRDIEQ-HSAGVESVFNICDVLLHDSDACANETECDSIQQTTRSLDRR
WRNICAMSMERRMKIEETWRLW*
>P1;3EDU_SR14
structure:3EDU_SR14
----GSLFQLKRETDDLEQWISEKELVASSPEM--GQDFDHVTLLRDKFR
DFARETGAIGQERVDNVNAFIERL-----IDAGHSEAATIAEWKDGLNEM
WADLLELIDTRMQLLAASYDLH*
>P1;3EDV_SR14
structure:3EDV_SR14
SHMRHRLFQLNREVDDLEQWIAEREVVAGSHEL--GQDYEHVTMLQERFR
EFARDTGNIGQERVDTVNHLADEL-----INSGHSDAATIAEWKDGLNEA
WADLLELIDTRTQILAASYELH*


>P1;NESPRIN2_SR52
sequence:NESPRIN2_SR52
RRMKIEETWRLWQKFLDDYSRFEDWLKSAERTAACPNSSEVLYTSAKEEL
KRFEAFQR----QIHERLTQLELINKQYRRLARENRTDT-ASRLKQMVHE
GNQRWDNLQRRVTAVLRRLRHFTNQRE*
>P1;1HCI_SR4
structure:1HCI_SR4
-----HANERLRRQFAAQANAIGPWIQNKMEEIARS----S-IQITGALE
DQMNQLKQY-EHNIINYKNNIDKLEGDHQLIQEALVFD--NKHTNYTMEH
IRVGWELLLTTIARTINEVETQILTRD*
>P1;1HCI_SR1
structure:1HCI_SR1
SSAVNQENERLMEEYERLASELLEWIRRTIPWLENRTPEKT-MQAMQKKL
EDFRDYRRKHKPPKVQEKCQLEINFNTLQTKLRISNRPAFMPSEGKMVSD
IAGAWQRLEQAEKGYEEWLLNEIR-RL*
```

Figure S12B: Alignments used to build the models of nesprin-2 SRs

```
>P1;NESPRIN2_SR53
sequence:NESPRIN2_SR53
----VLRRLRHFTNQREEFEGTRESILVWLTEMDLQLTNVEHFSESDADD
KMRQLNGFQQ----EITLNTNKIDQLIVFGEQLIQKSE-----PLDAVLI
EDELEELHRYCQEVFGRVSRFHRRLTSCTPGLE*
>P1;1HCI_SR4
structure:1HCI_SR4
-----HANERLRRQFAAQANAIGPWIQN---KMEEIARS----SIQITGA
LEDQMNQLKQY-EHNIINYKNNIDKLEGDHQLIQEALVFD--NKHTNYTM
EHIRVGWELLLTTIARTINEVETQILTRD----*
>P1;1HCI_SR1
structure:1HCI_SR1
SSAVNQENERLMEEYERLASELLEWIRR---TIPWLENRTPEKTMQAMQK
KLEDFRDYRRKHKPPKVQEKCQLEINFNTLQTKLRISNRPAFMPSEGKMV
SDIAGAWQRLEQAEKGYEEWLLNEIR-RL----*
```

```
>P1;NESPRIN2_SR54
sequence:NESPRIN2_SR54
------------SCPEHH--YKQMEGDRNVPPVPPASST--PYKPPYGKL
LLPPGTDGGKEGPRVLNGNPQQEDGG---LAGITEQQSGAFDRWEMIQAQ
ELHNKLKIKQNLQ---*
>P1;1HCI_SR4
structure:1HCI_SR4
HANERLRRQFAAQANAIGPWIQNKMEEIARSSI-QITGALEDQMNQLKQY
EHNIINY-KNNIDKLEGDHQLIQEALVFDNKHTNYTMEHIRVGWELLLTT
IARTINEVETQILTRD*
>P1;3EDV_SR14
structure:3EDV_SR14
SHMRHRLFQLNREVDDLEQWIAEREVVAGSHELGQDYEHVTMLQERFREF
ARDTGNIGQERVDTVNHLADELINSGHSDAATIAEWKDGLNEAWADLLEL
IDTRTQILAASYELH-*
```

```
>P1;NESPRIN2_SR55
sequence:NESPRIN2_SR55
---KQNLQQLNSDISAITTWLKKTEAELEMLKMAKPPSDIQEIELRVKRL
QEILKAFDT-YKALVVSVNVSSKEFLQTESPESTELQSRLRQLSLLWEAA
QGAVDSWRGGLRQSLMQC*
>P1;3EDU_SR14
structure:3EDU_SR14
----GSLFQLKRETDDLEQWISEKELVASSPE---MGQDFDHVTLLRDKF
RDFARETGAIGQERVDNVNAFIERLIDAGHSEAATIAEWKDGLNEMWADL
LELIDTRMQLLAASYDLH*
>P1;3EDV_SR14
structure:3EDV_SR14
SHMRHRLFQLNREVDDLEQWIAEREVVAGSHE---LGQDYEHVTMLQERF
REFARDTGNIGQERVDTVNHLADELINSGHSDAATIAEWKDGLNEAWADL
LELIDTRTQILAASYELH*
```

Figure S12B: Alignments used to build the models of nesprin-2 SRs

```
>P1;NESPRIN2_SR56
sequence:NESPRIN2_SR56
--GGLRQSLMQCQDFHQLSQNLLLWLASAKNRRQKAHVTDPKADPRALLE
CRRELMQL-EKELVERQPQVDMLQEISNSLLIKGHGED-CIEAEEKVHVI
EKKLKQLREQVSQDLMALQGTQN---*
>P1;1HCI_SR4
structure:1HCI_SR4
-----HANERLRRQFAAQANAIGPWIQNKMEEIARS----SIQITGALED
QMNQLKQY-EHNIINYKNNIDKLEGDHQLIQEALVFD--NKHTNYTMEHI
RVGWELLLTTIARTINEVETQILTRD*
>P1;1HCI_SR1
structure:1HCI_SR1
SSAVNQENERLMEEYERLASELLEWIRRTIPWLENRTPEKTMQAMQKKLE
DFRDYRRKHKPPKVQEKCQLEINFNTLQTKLRISNRPAFMPSEGKMVSDI
AGAWQRLEQAEKGYEEWLLNEIR-RL*
```

Figure S12: Alignment used to build the models of nesprin-1 (A) and nesprin-2 (B) SRs
