# Supplementary material for: Large-Scale Modelling of the Divergent Spectrin Repeats in Nesprins: Giant Modular Proteins
Source: PLoS One. 2013 May 6;8(5):e63633. doi: 10.1371/journal.pone.0063633 (PMC3646009; doi:10.1371/journal.pone.0063633)
Supplement: Table S1 — nesprin-1 SRs Z-Score from ProSA [28] In bolded are higlighed the SR assigned also by Pfam. (PDF) [file pone.0063633.s013.pdf]

Table S1: nesprin-1 SRs Z-Score from ProSA [28]
In bolded are higlighed the SR assigned also by Pfam.

MODEL	Z-Score
NES1-SR1	-4.01
NES1-SR2	-4.49
NES1-SR3	-3.87
NES1-SR4	-1.62
NES1-SR5	-3.85
NES1-SR6	-4.41
NES1-SR7	-3.07
NES1-SR8	-4
NES1-SR9	-5.9
NES1-SR10	-4.36
NES1-SR11	-2.92
**NES1-SR12**	**-5.88**
**NES1-SR13**	**-6**
**NES1-SR14**	**-6.07**
NES1-SR15	-5.54
NES1-SR16	-3.61
**NES1-SR17**	**-5.31**
NES1-SR18	-4.89
NES1-SR19	-5.57
**NES1-SR20**	**-6.11**
**NES1-SR21**	**-5.34**
**NES1-SR22**	**-5.09**
NES1-SR23	-5.06
NES1-SR24	-3.35
**NES1-SR25**	**-6.52**
NES1-SR26	-5.07
**NES1-SR27**	**-5.03**
**NES1-SR28**	**-5.1**
**NES1-SR29**	**-5.27**
NES1-SR30	-4.83
**NES1-SR31**	**-4.99**
**NES1-SR32**	**-5.18**
NES1-SR33	-3.94
**NES1-SR34**	**-5.28**
**NES1-SR35**	**-5.12**
NES1-SR36	-5.07
**NES1-SR37**	**-5.03**
**NES1-SR38**	**-5.1**
**NES1-SR39**	**-5.27**
**NES1-SR40**	**-6.02**
NES1-SR41	-5.17
NES1-SR42	-5.32
NES1-SR43	-5.19
**NES1-SR44**	**-4.91**
**NES1-SR45**	**-5.19**
**NES1-SR46**	**-6.16**
NES1-SR47	-5.86
NES1-SR48	-5.07
NES1-SR49	-4.98
**NES1-SR50**	**-5.56**

NES1-SR51		-5.17
NES1-SR52	N/A	
**NES1-SR53**		**-5.66**
NES1-SR54	N/A	
NES1-SR55		-4.52
NES1-SR56		-4.74
NES1-SR57		-3.17
NES1-SR58		-4.21
**NES1-SR59**		**-5.31**
**NES1-SR60**		**-4.47**
**NES1-SR61**		**-6.22**
**NES1-SR62**		**-5.74**
NES1-SR63		-4.61
**NES1-SR64**		**-6.3**
NES1-SR65		-5.2
NES1-SR66		-4.33
**NES1-SR67**		**-6.07**
**NES1-SR68**		**-5.06**
**NES1-SR69**		**-5.64**
**NES1-SR70**		**-5.67**
**NES1-SR71**		**-6.17**
NES1-SR72		-4.34
**NES1-SR73**		**-5.19**
NES1-SR74		-4.8
