# Supplementary material for: Large-Scale Modelling of the Divergent Spectrin Repeats in Nesprins: Giant Modular Proteins
Source: PLoS One. 2013 May 6;8(5):e63633. doi: 10.1371/journal.pone.0063633 (PMC3646009; doi:10.1371/journal.pone.0063633)
Supplement: Table S2 — nesprin-2 SRs Z-Score from ProSA [28] In bolded are higlighed the SR assigned also by Pfam. (PDF) [file pone.0063633.s014.pdf]

Table S2: nesprin-2 SRs Z-Score from ProSA [28]
In bolded are higlighed the SR assigned also by Pfam.

MODEL	Z-Score
NES2-SR1	-4.52
NES2-SR2	-3.9
NES2-SR3	-4.47
NES2-SR4	-2.46
NES2-SR5	-3.96
NES2-SR6	-5.71
NES2-SR7	-3.83
NES2-SR8	-3.68
NES2-SR9	-4.45
NES2-SR10	-4.04
NES2-SR11	-6.29
NES2-SR12	-4.76
NES2-SR13	-4.23
NES2-SR14	-4.55
NES2-SR15	-5.38
NES2-SR16	-2.92
NES2-SR17	-5.09
NES2-SR18	-4.2
**NES2-SR19**	**-5.3**
NES2-SR20	-3.86
**NES2-SR21**	**-4.8**
NES2-SR22	-4.66
**NES2-SR23**	**-5.14**
NES2-SR24	-3.15
NES2-SR25	-3.98
NES2-SR26	-4.52
NES2-SR27	-4.59
NES2-SR28	-3.98
NES2-SR29	-4.97
NES2-SR30	-4.55
NES2-SR31	-4.6
NES2-SR32	-6.02
NES2-SR33	-4.85
NES2-SR34	-4.11
NES2-SR35	-1.86
NES2-SR36	-4.37
NES2-SR37	-5.17
NES2-SR38	-4.76
NES2-SR39	-4.01
NES2-SR40	-4.62
**NES2-SR41**	**-5.69**
**NES2-SR42**	**-4.72**
**NES2-SR43**	**-4.7**
**NES2-SR44**	**-5.24**
NES2-SR45	-4.24
NES2-SR46	-3.19
**NES2-SR47**	**-5.35**
**NES2-SR48**	**-4.95**
**NES2-SR49**	**-4.67**
**NES2-SR50**	**-4.27**

**NES2-SR51**	**-5.72**
**NES2-SR52**	**-5.33**
**NES2-SR53**	**-6.02**
NES2-SR54	-3.37
**NES2-SR55**	**-6.03**
NES2-SR56	-5.76
