# Supplementary material for: Large-Scale Modelling of the Divergent Spectrin Repeats in Nesprins: Giant Modular Proteins
Source: PLoS One. 2013 May 6;8(5):e63633. doi: 10.1371/journal.pone.0063633 (PMC3646009; doi:10.1371/journal.pone.0063633)
Supplement: Table S3 — Nesprin1α hot spots. (PDF) [file pone.0063633.s015.pdf]

Table S3: Nesprin1α hot spots

NES1-SR69						
Cluster centre	Conservation	SASA (Å2)	neighbours	Conservation	SASA (Å2)	Distance Å
ALA 7886	9	43.16	LEU 7884	8	204.44	6.113
ALA 7886	9	43.16	GLN 7888	9	87.32	5.439
ALA 7886	9	43.16	GLN 7889	9	96.34	5.113
ALA 7886	9	43.16	ASP 7891	9	88.34	8.513
ALA 7886	9	43.16	LYS 7892	8	178.59	9.987
ALA 7886	9	43.16	LEU 7949	8	49.03	8.211
ALA 7886	9	43.16	VAL 7952	9	53.63	9.987
ALA 7886	9	43.16	ASP 7958	9	121.3	8.576
ALA 7886	9	43.16	GLU 7965	8	66.26	9.481
GLN 7888	9	87.32	LEU 7884	8	204.44	6.733
GLN 7888	9	87.32	VAL 7885	9	52.75	4.766
GLN 7888	9	87.32	ALA 7886	9	43.16	5.439
GLN 7888	9	87.32	ASP 7891	9	88.34	4.963
GLN 7888	9	87.32	LYS 7892	8	178.59	6.27
GLN 7888	9	87.32	GLU 7965	8	66.26	9.548
GLN 7889	9	96.34	LEU 7884	8	204.44	5.853
GLN 7889	9	96.34	VAL 7885	9	52.75	5.606
GLN 7889	9	96.34	ALA 7886	9	43.16	5.113
GLN 7889	9	96.34	ASP 7891	9	88.34	5.407
GLN 7889	9	96.34	LYS 7892	8	178.59	5.245
GLN 7889	9	96.34	LEU 7949	8	49.03	8.877
ASP 7891	9	88.34	VAL 7885	9	52.75	9.406
ASP 7891	9	88.34	ALA 7886	9	43.16	8.513
ASP 7891	9	88.34	GLN 7888	9	87.32	4.963
ASP 7891	9	88.34	GLN 7889	9	96.34	5.407
ASP 7891	9	88.34	SER 7896	9	32.51	8.703
ASP 7891	9	88.34	LEU 7949	8	49.03	9.922
ASP 7891	9	88.34	SER 7968	9	69.05	9.521
TYR 7914	7	73.05	PRO 7911	8	76.22	9.394
TYR 7914	7	73.05	ILE 7912	9	68.54	6.16
TYR 7914	7	73.05	GLN 7923	9	130.17	8.59
TYR 7914	7	73.05	ARG 7924	9	111.64	8.506
TYR 7914	7	73.05	LEU 7926	8	48.57	9.87
TYR 7914	7	73.05	THR 7997	9	87.89	9.324
LEU 7926	8	48.57	ILE 7912	9	68.54	9.816
LEU 7926	8	48.57	TYR 7914	7	73.05	9.87
LEU 7926	8	48.57	GLN 7923	9	130.17	5.236
LEU 7926	8	48.57	ARG 7924	9	111.64	5.524
LEU 7926	8	48.57	GLN 7930	9	104.02	5.978
LEU 7926	8	48.57	ARG 7991	9	170.11	8.408
LEU 7926	8	48.57	GLU 7995	8	106.6	7.961
LEU 7926	8	48.57	THR 7997	9	87.89	9.336
GLN 7930	9	104.02	ARG 7924	9	111.64	9.676

GLN 7930	9	104.02	LEU 7926	8	48.57	5.978
GLN 7930	9	104.02	GLN 7933	9	89.2	4.795
GLN 7930	9	104.02	ARG 7934	9	164.05	6.061
GLN 7930	9	104.02	ASP 7935	9	51.13	8.487
GLN 7930	9	104.02	ARG 7991	9	170.11	8.196
GLN 7933	9	89.2	GLN 7930	9	104.02	4.795
GLN 7933	9	89.2	ASP 7935	9	51.13	5.282
GLN 7933	9	89.2	GLU 7937	8	105.41	6.44
GLN 7933	9	89.2	LYS 7938	8	112.25	8.684
GLN 7933	9	89.2	HIS 7939	9	46.41	9.8
GLN 7933	9	89.2	MET 7988	9	117.43	9.24
GLN 7933	9	89.2	ARG 7991	9	170.11	8.968
ASP 7935	9	51.13	GLN 7930	9	104.02	8.487
ASP 7935	9	51.13	GLN 7933	9	89.2	5.282
ASP 7935	9	51.13	GLU 7937	8	105.41	5.189
ASP 7935	9	51.13	LYS 7938	8	112.25	4.76
ASP 7935	9	51.13	HIS 7939	9	46.41	5.937
GLU 7937	8	105.41	GLN 7933	9	89.2	6.44
GLU 7937	8	105.41	ARG 7934	9	164.05	5.153
GLU 7937	8	105.41	ASP 7935	9	51.13	5.189
GLU 7937	8	105.41	HIS 7939	9	46.41	5.494
GLU 7937	8	105.41	THR 7941	9	84.49	7.788
HIS 7939	9	46.41	SER 7896	9	32.51	9.894
HIS 7939	9	46.41	GLN 7933	9	89.2	9.8
HIS 7939	9	46.41	ARG 7934	9	164.05	8.718
HIS 7939	9	46.41	ASP 7935	9	51.13	5.937
HIS 7939	9	46.41	GLU 7937	8	105.41	5.494
HIS 7939	9	46.41	THR 7941	9	84.49	5.354
HIS 7939	9	46.41	ALA 7944	9	31.51	8.414
THR 7941	9	84.49	GLU 7937	8	105.41	7.788
THR 7941	9	84.49	LYS 7938	8	112.25	6.701
THR 7941	9	84.49	HIS 7939	9	46.41	5.354
THR 7941	9	84.49	ALA 7944	9	31.51	4.989
THR 7941	9	84.49	ASN 7948	9	93.59	9.849
ALA 7944	9	31.51	HIS 7939	9	46.41	8.414
ALA 7944	9	31.51	THR 7941	9	84.49	4.989
ALA 7944	9	31.51	ASN 7948	9	93.59	6.305
ALA 7944	9	31.51	LEU 7949	8	49.03	8.667
ALA 7944	9	31.51	ASP 7977	9	61.32	7.861
ASN 7948	9	93.59	THR 7941	9	84.49	9.849
ASN 7948	9	93.59	ALA 7944	9	31.51	6.305
ASN 7948	9	93.59	GLU 7951	8	140.61	5.42
ASN 7948	9	93.59	VAL 7952	9	53.63	6.108
ASN 7948	9	93.59	LEU 7954	8	54.76	9.898
LEU 7949	8	49.03	ALA 7886	9	43.16	8.211

LEU 7949	8	49.03	GLN 7889	9	96.34	8.877
LEU 7949	8	49.03	ASP 7891	9	88.34	9.922
LEU 7949	8	49.03	ALA 7944	9	31.51	8.667
LEU 7949	8	49.03	GLU 7951	8	140.61	5.59
LEU 7949	8	49.03	VAL 7952	9	53.63	5.126
LEU 7949	8	49.03	LEU 7954	8	54.76	8.474
LEU 7949	8	49.03	HIS 7955	9	133.08	9.828
GLU 7951	8	140.61	ASN 7948	9	93.59	5.42
GLU 7951	8	140.61	LEU 7949	8	49.03	5.59
GLU 7951	8	140.61	LEU 7954	8	54.76	4.915
GLU 7951	8	140.61	HIS 7955	9	133.08	6.096
GLU 7951	8	140.61	ASP 7956	9	74.67	8.504
GLU 7951	8	140.61	GLN 7970	9	78.44	7.447
GLU 7951	8	140.61	ARG 7974	9	100.06	9.693
VAL 7952	9	53.63	ALA 7886	9	43.16	9.987
VAL 7952	9	53.63	ASN 7948	9	93.59	6.108
VAL 7952	9	53.63	LEU 7949	8	49.03	5.126
VAL 7952	9	53.63	LEU 7954	8	54.76	5.287
VAL 7952	9	53.63	HIS 7955	9	133.08	5.016
VAL 7952	9	53.63	ASP 7956	9	74.67	5.986
VAL 7952	9	53.63	THR 7962	9	53.43	8.962
LEU 7954	8	54.76	ASN 7948	9	93.59	9.898
LEU 7954	8	54.76	LEU 7949	8	49.03	8.474
LEU 7954	8	54.76	GLU 7951	8	140.61	4.915
LEU 7954	8	54.76	VAL 7952	9	53.63	5.287
LEU 7954	8	54.76	ASP 7956	9	74.67	5.55
LEU 7954	8	54.76	ALA 7961	9	64.92	9.078
LEU 7954	8	54.76	THR 7962	9	53.43	5.362
LEU 7954	8	54.76	GLU 7965	8	66.26	8.008
LEU 7954	8	54.76	SER 7968	9	69.05	9.945
LEU 7954	8	54.76	GLN 7970	9	78.44	7.496
HIS 7955	9	133.08	LEU 7949	8	49.03	9.828
HIS 7955	9	133.08	GLU 7951	8	140.61	6.096
HIS 7955	9	133.08	VAL 7952	9	53.63	5.016
HIS 7955	9	133.08	CYS 7960	7	111.85	9.675
HIS 7955	9	133.08	ALA 7961	9	64.92	8.544
HIS 7955	9	133.08	THR 7962	9	53.43	5.642
ASP 7956	9	74.67	GLU 7951	8	140.61	8.504
ASP 7956	9	74.67	VAL 7952	9	53.63	5.986
ASP 7956	9	74.67	LEU 7954	8	54.76	5.55
ASP 7956	9	74.67	ASP 7958	9	121.3	6.814
ASP 7956	9	74.67	ALA 7959	9	42.69	7.444
ASP 7956	9	74.67	CYS 7960	7	111.85	6.579
ASP 7956	9	74.67	ALA 7961	9	64.92	6.437
ASP 7956	9	74.67	THR 7962	9	53.43	4.497
ASP 7958	9	121.3	ALA 7886	9	43.16	8.576
ASP 7958	9	121.3	ASP 7956	9	74.67	6.814

ASP 7958	9	121.3	CYS 7960	7	111.85	6.293
ASP 7958	9	121.3	ALA 7961	9	64.92	7.97
ASP 7958	9	121.3	THR 7962	9	53.43	8.195
ALA 7961	9	64.92	LEU 7954	8	54.76	9.078
ALA 7961	9	64.92	HIS 7955	9	133.08	8.544
ALA 7961	9	64.92	ASP 7956	9	74.67	6.437
ALA 7961	9	64.92	ASP 7958	9	121.3	7.97
ALA 7961	9	64.92	ALA 7959	9	42.69	5.314
THR 7962	9	53.43	VAL 7952	9	53.63	8.962
THR 7962	9	53.43	LEU 7954	8	54.76	5.362
THR 7962	9	53.43	HIS 7955	9	133.08	5.642
THR 7962	9	53.43	ASP 7956	9	74.67	4.497
THR 7962	9	53.43	ASP 7958	9	121.3	8.195
THR 7962	9	53.43	ALA 7959	9	42.69	7.079
THR 7962	9	53.43	CYS 7960	7	111.85	6.42
THR 7962	9	53.43	GLU 7965	8	66.26	8.46
GLU 7965	8	66.26	ALA 7886	9	43.16	9.481
GLU 7965	8	66.26	GLN 7888	9	87.32	9.548
GLU 7965	8	66.26	LEU 7954	8	54.76	8.008
GLU 7965	8	66.26	THR 7962	9	53.43	8.46
GLU 7965	8	66.26	SER 7968	9	69.05	5.518
GLU 7965	8	66.26	GLN 7970	9	78.44	8.574
SER 7968	9	69.05	ASP 7891	9	88.34	9.521
SER 7968	9	69.05	LEU 7954	8	54.76	9.945
SER 7968	9	69.05	GLU 7965	8	66.26	5.518
SER 7968	9	69.05	GLN 7970	9	78.44	5.535
SER 7968	9	69.05	GLN 7971	9	126.02	5.394
GLN 7970	9	78.44	GLU 7951	8	140.61	7.447
GLN 7970	9	78.44	LEU 7954	8	54.76	7.496
GLN 7970	9	78.44	GLU 7965	8	66.26	8.574
GLN 7970	9	78.44	SER 7968	9	69.05	5.535
GLN 7970	9	78.44	ARG 7974	9	100.06	6.07
ARG 7974	9	100.06	GLU 7951	8	140.61	9.693
ARG 7974	9	100.06	GLN 7970	9	78.44	6.07
ARG 7974	9	100.06	GLN 7971	9	126.02	5.371
ARG 7974	9	100.06	ASP 7977	9	61.32	5.055
ARG 7974	9	100.06	ARG 7978	9	187.79	5.975
ARG 7991	9	170.11	LEU 7926	8	48.57	8.408
ARG 7991	9	170.11	GLN 7930	9	104.02	8.196
ARG 7991	9	170.11	GLN 7933	9	89.2	8.968
ARG 7991	9	170.11	ALA 7985	9	35.85	9.982
ARG 7991	9	170.11	MET 7988	9	117.43	5.209
ARG 7991	9	170.11	GLU 7989	8	80.03	5.298
ARG 7991	9	170.11	GLU 7995	8	106.6	5.926
ARG 7991	9	170.11	GLU 7996	8	134.38	8.468

NES1-SR70

Cluster centre	Conservation	SASA (Å2)	neighbours	Conservation	SASA (Å2)	Distance Å
TRP 7998	7	191.38	LEU 8000	8	84.4	5.093
TRP 7998	7	191.38	TRP 8001	7	72.87	6.928
TRP 7998	7	191.38	GLN 8002	9	116.23	8.09
TRP 7998	7	191.38	ARG 8065	9	189.81	9.029
TRP 7998	7	191.38	ASN 8070	9	74.57	9.886
ARG 7999	9	194.49	TRP 8001	7	72.87	5.93
ARG 7999	9	194.49	GLN 8002	9	116.23	8.424
ARG 7999	9	194.49	ARG 8065	9	189.81	9.482
ARG 7999	9	194.49	ASN 8070	9	74.57	8.088
ARG 7999	9	194.49	ARG 8071	9	253.24	9.106
ARG 7999	9	194.49	ASP 8073	9	144.5	8.861
TRP 8001	7	72.87	TRP 7998	7	191.38	6.928
TRP 8001	7	72.87	ARG 7999	9	194.49	5.93
TRP 8001	7	72.87	ASP 8006	9	82.68	8.388
TRP 8001	7	72.87	ASP 8007	9	42.21	9.997
TRP 8001	7	72.87	ASP 8073	9	144.5	9.36
GLN 8002	9	116.23	TRP 7998	7	191.38	8.09
GLN 8002	9	116.23	ARG 7999	9	194.49	8.424
GLN 8002	9	116.23	LEU 8000	8	84.4	5.309
GLN 8002	9	116.23	ASP 8006	9	82.68	5.791
GLN 8002	9	116.23	ASP 8007	9	42.21	8.461
GLN 8002	9	116.23	SER 8009	9	49.09	9.959
ASP 8007	9	42.21	TRP 8001	7	72.87	9.997
ASP 8007	9	42.21	GLN 8002	9	116.23	8.461
ASP 8007	9	42.21	SER 8009	9	49.09	5.399
ASP 8007	9	42.21	GLU 8012	9	41.5	8.783
ASP 8007	9	42.21	GLN 8055	9	94.08	7.364
ASP 8007	9	42.21	GLU 8057	9	63.47	9.506
ASP 8007	9	42.21	LEU 8058	8	50.31	7.188
ASP 8007	9	42.21	ASN 8060	9	44.27	9.256
SER 8009	9	49.09	GLN 8002	9	116.23	9.959
SER 8009	9	49.09	ASP 8006	9	82.68	5.282
SER 8009	9	49.09	ASP 8007	9	42.21	5.399
SER 8009	9	49.09	GLU 8012	9	41.5	5.129
SER 8009	9	49.09	TRP 8014	7	94.67	8.781
GLU 8012	9	41.5	ASP 8007	9	42.21	8.783
GLU 8012	9	41.5	SER 8009	9	49.09	5.129
GLU 8012	9	41.5	TRP 8014	7	94.67	5.5
GLU 8012	9	41.5	GLN 8055	9	94.08	9.742
GLU 8012	9	41.5	TRP 8089	7	73.34	9.513
TRP 8014	7	94.67	SER 8009	9	49.09	8.781
TRP 8014	7	94.67	GLU 8012	9	41.5	5.5
TRP 8014	7	94.67	VAL 8049	9	31.21	8.554

TRP 8014	7	94.67	GLN 8055	9	94.08	8.292
TRP 8014	7	94.67	TRP 8089	7	73.34	9.903
LYS 8036	8	161.77	TYR 8032	8	199.73	6.101
LYS 8036	8	161.77	LEU 8039	8	52.66	5.226
LYS 8036	8	161.77	LYS 8040	8	136.48	6.289
LYS 8036	8	161.77	LYS 8041	8	78.05	8.589
LYS 8036	8	161.77	LYS 8104	8	115.44	9.99
GLU 8037	9	73.43	TYR 8032	8	199.73	8.574
GLU 8037	9	73.43	LEU 8039	8	52.66	5.372
GLU 8037	9	73.43	LYS 8040	8	136.48	4.954
GLU 8037	9	73.43	LYS 8041	8	78.05	5.929
GLU 8037	9	73.43	GLU 8043	9	91.15	9.594
LEU 8039	8	52.66	LYS 8036	8	161.77	5.226
LEU 8039	8	52.66	GLU 8037	9	73.43	5.372
LEU 8039	8	52.66	LYS 8041	8	78.05	5.419
LEU 8039	8	52.66	GLU 8043	9	91.15	6.1
LEU 8039	8	52.66	LEU 8100	8	40.87	8.252
LEU 8039	8	52.66	LYS 8104	8	115.44	7.816
LYS 8041	8	78.05	LYS 8036	8	161.77	8.589
LYS 8041	8	78.05	GLU 8037	9	73.43	5.929
LYS 8041	8	78.05	LEU 8039	8	52.66	5.419
LYS 8041	8	78.05	GLU 8043	9	91.15	5.407
LYS 8041	8	78.05	GLN 8046	9	84.55	8.66
LYS 8041	8	78.05	ARG 8047	9	94.2	9.781
GLU 8043	9	91.15	GLU 8037	9	73.43	9.594
GLU 8043	9	91.15	LEU 8039	8	52.66	6.1
GLU 8043	9	91.15	LYS 8040	8	136.48	4.89
GLU 8043	9	91.15	LYS 8041	8	78.05	5.407
GLU 8043	9	91.15	GLN 8046	9	84.55	4.792
GLU 8043	9	91.15	ARG 8047	9	94.2	6.131
GLU 8043	9	91.15	VAL 8049	9	31.21	9.726
GLU 8043	9	91.15	LEU 8100	8	40.87	8.239
GLN 8046	9	84.55	LYS 8040	8	136.48	9.51
GLN 8046	9	84.55	LYS 8041	8	78.05	8.66
GLN 8046	9	84.55	GLU 8043	9	91.15	4.792
GLN 8046	9	84.55	VAL 8049	9	31.21	5.142
GLN 8046	9	84.55	GLU 8051	9	98.59	8.667
GLN 8046	9	84.55	GLN 8093	9	80.59	9.519
GLN 8046	9	84.55	LEU 8100	8	40.87	8.867
VAL 8049	9	31.21	TRP 8014	7	94.67	8.554
VAL 8049	9	31.21	GLU 8043	9	91.15	9.726
VAL 8049	9	31.21	GLN 8046	9	84.55	5.142
VAL 8049	9	31.21	ARG 8047	9	94.2	5.511
VAL 8049	9	31.21	GLU 8051	9	98.59	5.378
VAL 8049	9	31.21	THR 8054	9	92.78	9.354
VAL 8049	9	31.21	GLN 8055	9	94.08	9.868

VAL 8049	9	31.21	TRP 8089	7	73.34	9.918
VAL 8049	9	31.21	GLN 8093	9	80.59	8.587
GLU 8051	9	98.59	GLN 8046	9	84.55	8.667
GLU 8051	9	98.59	ARG 8047	9	94.2	6.29
GLU 8051	9	98.59	VAL 8049	9	31.21	5.378
GLU 8051	9	98.59	THR 8054	9	92.78	6.801
GLU 8051	9	98.59	GLN 8055	9	94.08	8.211
THR 8054	9	92.78	VAL 8049	9	31.21	9.354
THR 8054	9	92.78	GLU 8051	9	98.59	6.801
THR 8054	9	92.78	GLU 8057	9	63.47	5.1
THR 8054	9	92.78	LEU 8058	8	50.31	5.73
THR 8054	9	92.78	ASN 8060	9	44.27	9.932
THR 8054	9	92.78	LYS 8061	8	108.87	9.916
GLN 8055	9	94.08	ASP 8007	9	42.21	7.364
GLN 8055	9	94.08	GLU 8012	9	41.5	9.742
GLN 8055	9	94.08	TRP 8014	7	94.67	8.292
GLN 8055	9	94.08	VAL 8049	9	31.21	9.868
GLN 8055	9	94.08	GLU 8051	9	98.59	8.211
GLN 8055	9	94.08	GLU 8057	9	63.47	5.597
GLN 8055	9	94.08	LEU 8058	8	50.31	5.28
GLN 8055	9	94.08	ASN 8060	9	44.27	9.433
GLU 8057	9	63.47	ASP 8007	9	42.21	9.506
GLU 8057	9	63.47	THR 8054	9	92.78	5.1
GLU 8057	9	63.47	GLN 8055	9	94.08	5.597
GLU 8057	9	63.47	ASN 8060	9	44.27	5.191
GLU 8057	9	63.47	LYS 8061	8	108.87	6.306
GLU 8057	9	63.47	ASN 8086	9	57.02	7.978
GLU 8057	9	63.47	TRP 8089	7	73.34	8.444
LEU 8058	8	50.31	ASP 8007	9	42.21	7.188
LEU 8058	8	50.31	THR 8054	9	92.78	5.73
LEU 8058	8	50.31	GLN 8055	9	94.08	5.28
LEU 8058	8	50.31	ASN 8060	9	44.27	5.327
LEU 8058	8	50.31	LYS 8061	8	108.87	5.133
ASN 8060	9	44.27	ASP 8007	9	42.21	9.256
ASN 8060	9	44.27	THR 8054	9	92.78	9.932
ASN 8060	9	44.27	GLN 8055	9	94.08	9.433
ASN 8060	9	44.27	GLU 8057	9	63.47	5.191
ASN 8060	9	44.27	LEU 8058	8	50.31	5.327
ASN 8060	9	44.27	ARG 8065	9	189.81	8.514
ASN 8060	9	44.27	HIS 8083	9	111.15	7.99
ASN 8060	9	44.27	ASN 8086	9	57.02	7.099
LYS 8061	8	108.87	THR 8054	9	92.78	9.916
LYS 8061	8	108.87	GLU 8057	9	63.47	6.306
LYS 8061	8	108.87	LEU 8058	8	50.31	5.133
LYS 8061	8	108.87	ARG 8065	9	189.81	6.231
LYS 8061	8	108.87	ARG 8068	9	172.19	9.888

ARG 8065	9	189.81	TRP 7998	7	191.38	9.029
ARG 8065	9	189.81	ARG 7999	9	194.49	9.482
ARG 8065	9	189.81	ASN 8060	9	44.27	8.514
ARG 8065	9	189.81	LYS 8061	8	108.87	6.231
ARG 8065	9	189.81	ARG 8068	9	172.19	5.442
ARG 8065	9	189.81	GLU 8069	9	108.31	4.385
ARG 8065	9	189.81	ASN 8070	9	74.57	6.341
ARG 8065	9	189.81	ARG 8071	9	253.24	9.032
ARG 8068	9	172.19	LYS 8061	8	108.87	9.888
ARG 8068	9	172.19	ARG 8065	9	189.81	5.442
ARG 8068	9	172.19	ASN 8070	9	74.57	5.615
ARG 8068	9	172.19	ARG 8071	9	253.24	5.788
ARG 8068	9	172.19	ASP 8073	9	144.5	9.471
ARG 8068	9	172.19	CYS 8076	8	88.69	8.682
ASN 8070	9	74.57	TRP 7998	7	191.38	9.886
ASN 8070	9	74.57	ARG 7999	9	194.49	8.088
ASN 8070	9	74.57	ARG 8065	9	189.81	6.341
ASN 8070	9	74.57	ARG 8068	9	172.19	5.615
ASN 8070	9	74.57	ASP 8073	9	144.5	9.232
ARG 8071	9	253.24	ARG 7999	9	194.49	9.106
ARG 8071	9	253.24	ARG 8065	9	189.81	9.032
ARG 8071	9	253.24	ARG 8068	9	172.19	5.788
ARG 8071	9	253.24	GLU 8069	9	108.31	5.882
ARG 8071	9	253.24	ASP 8073	9	144.5	6.427
ARG 8071	9	253.24	CYS 8076	8	88.69	9.941
ASP 8073	9	144.5	ARG 7999	9	194.49	8.861
ASP 8073	9	144.5	LEU 8000	8	84.4	9.842
ASP 8073	9	144.5	TRP 8001	7	72.87	9.36
ASP 8073	9	144.5	ARG 8068	9	172.19	9.471
ASP 8073	9	144.5	ASN 8070	9	74.57	9.232
ASP 8073	9	144.5	ARG 8071	9	253.24	6.427
ASP 8073	9	144.5	CYS 8076	8	88.69	7.163
ASN 8086	9	57.02	GLU 8057	9	63.47	7.978
ASN 8086	9	57.02	ASN 8060	9	44.27	7.099
ASN 8086	9	57.02	HIS 8083	9	111.15	5.133
ASN 8086	9	57.02	TRP 8089	7	73.34	4.984
ASN 8086	9	57.02	ASP 8090	9	32.86	6.059
TRP 8089	7	73.34	GLU 8012	9	41.5	9.513
TRP 8089	7	73.34	TRP 8014	7	94.67	9.903
TRP 8089	7	73.34	VAL 8049	9	31.21	9.918
TRP 8089	7	73.34	GLU 8057	9	63.47	8.444
TRP 8089	7	73.34	HIS 8083	9	111.15	9.777
TRP 8089	7	73.34	ASN 8086	9	57.02	4.984
TRP 8089	7	73.34	GLN 8093	9	80.59	6.239
TRP 8089	7	73.34	ARG 8095	9	125.07	9.884

GLN 8093	9	80.59	GLN 8046	9	84.55	9.519
GLN 8093	9	80.59	VAL 8049	9	31.21	8.587
GLN 8093	9	80.59	TRP 8089	7	73.34	6.239
GLN 8093	9	80.59	ASP 8090	9	32.86	5.483
GLN 8093	9	80.59	ARG 8095	9	125.07	5.426
ARG 8095	9	125.07	ALA 8023	9	60.78	8.716
ARG 8095	9	125.07	TRP 8089	7	73.34	9.884
ARG 8095	9	125.07	ASP 8090	9	32.86	8.571
ARG 8095	9	125.07	GLN 8093	9	80.59	5.426
ARG 8095	9	125.07	LEU 8100	8	40.87	8.766
LEU 8100	8	40.87	LEU 8039	8	52.66	8.252
LEU 8100	8	40.87	GLU 8043	9	91.15	8.239
LEU 8100	8	40.87	GLN 8046	9	84.55	8.867
LEU 8100	8	40.87	ARG 8095	9	125.07	8.766
LEU 8100	8	40.87	ARG 8102	9	106.1	5.436
LEU 8100	8	40.87	LYS 8104	8	115.44	6.291
LEU 8100	8	40.87	HIS 8105	9	144.58	8.676
LEU 8100	8	40.87	PHE 8106	8	136.28	9.845
ARG 8102	9	106.1	PRO 8025	8	60.62	8.905
ARG 8102	9	106.1	LEU 8100	8	40.87	5.436
ARG 8102	9	106.1	LYS 8104	8	115.44	5.321
ARG 8102	9	106.1	HIS 8105	9	144.58	5.065
ARG 8102	9	106.1	PHE 8106	8	136.28	5.542
LYS 8104	8	115.44	LYS 8036	8	161.77	9.99
LYS 8104	8	115.44	LEU 8039	8	52.66	7.816
LYS 8104	8	115.44	LEU 8100	8	40.87	6.291
LYS 8104	8	115.44	ARG 8101	9	135.92	5.107
LYS 8104	8	115.44	ARG 8102	9	106.1	5.321
LYS 8104	8	115.44	PHE 8106	8	136.28	5.616
PHE 8106	8	136.28	SER 8027	9	78.98	9.317
PHE 8106	8	136.28	SER 8028	9	65.34	7.234
PHE 8106	8	136.28	LEU 8100	8	40.87	9.845
PHE 8106	8	136.28	ARG 8101	9	135.92	8.268
PHE 8106	8	136.28	ARG 8102	9	106.1	5.542
PHE 8106	8	136.28	LYS 8104	8	115.44	5.616

NES1-SR71

Cluster centre	Conservation	SASA (Å2)	neighbours	Conservation	SASA (Å2)	Distance Å
GLU 8111	9	59.14	ILE 8107	9	145.21	5.992
GLU 8111	9	59.14	GLN 8109	9	112.53	5.37
GLU 8111	9	59.14	GLU 8114	9	59.96	5.087
GLU 8111	9	59.14	THR 8115	9	43.76	6.146
GLU 8111	9	59.14	ARG 8117	9	59.48	9.699
GLU 8112	9	55.47	ILE 8107	9	145.21	6.403
GLU 8112	9	55.47	GLN 8109	9	112.53	5.31
GLU 8112	9	55.47	ARG 8110	9	130.91	5.488

GLU 8112	9	55.47	GLU 8114	9	59.96	5.458
GLU 8112	9	55.47	THR 8115	9	43.76	5.089
GLU 8112	9	55.47	ARG 8117	9	59.48	8.407
GLU 8112	9	55.47	ASP 8118	9	55.04	9.857
GLU 8114	9	59.96	GLN 8109	9	112.53	8.657
GLU 8114	9	59.96	ARG 8110	9	130.91	6.078
GLU 8114	9	59.96	GLU 8111	9	59.14	5.087
GLU 8114	9	59.96	GLU 8112	9	55.47	5.458
GLU 8114	9	59.96	ARG 8117	9	59.48	4.986
GLU 8114	9	59.96	ASP 8118	9	55.04	6.174
THR 8115	9	43.76	ARG 8110	9	130.91	8.546
THR 8115	9	43.76	GLU 8111	9	59.14	6.146
THR 8115	9	43.76	GLU 8112	9	55.47	5.089
THR 8115	9	43.76	ARG 8117	9	59.48	5.349
THR 8115	9	43.76	ASP 8118	9	55.04	5.137
THR 8115	9	43.76	LEU 8121	8	64.06	9.866
ARG 8117	9	59.48	GLU 8111	9	59.14	9.699
ARG 8117	9	59.48	GLU 8112	9	55.47	8.407
ARG 8117	9	59.48	GLU 8114	9	59.96	4.986
ARG 8117	9	59.48	THR 8115	9	43.76	5.349
ARG 8117	9	59.48	LEU 8121	8	64.06	5.772
ARG 8117	9	59.48	VAL 8122	9	63.14	8.351
ARG 8117	9	59.48	TRP 8123	7	40.93	9.708
ARG 8117	9	59.48	LEU 8189	8	35.7	8.893
ASP 8118	9	55.04	GLU 8112	9	55.47	9.857
ASP 8118	9	55.04	GLU 8114	9	59.96	6.174
ASP 8118	9	55.04	THR 8115	9	43.76	5.137
ASP 8118	9	55.04	LEU 8121	8	64.06	5.144
ASP 8118	9	55.04	VAL 8122	9	63.14	6.164
ASP 8118	9	55.04	TRP 8123	7	40.93	8.63
LEU 8121	8	64.06	THR 8115	9	43.76	9.866
LEU 8121	8	64.06	ARG 8117	9	59.48	5.772
LEU 8121	8	64.06	ASP 8118	9	55.04	5.144
LEU 8121	8	64.06	TRP 8123	7	40.93	5.393
LEU 8121	8	64.06	THR 8125	9	63.72	6.215
LEU 8121	8	64.06	GLU 8126	9	101.94	8.553
LEU 8121	8	64.06	GLU 8191	9	54.31	9.521
LEU 8121	8	64.06	TYR 8195	8	86.86	9.029
VAL 8122	9	63.14	ARG 8117	9	59.48	8.351
VAL 8122	9	63.14	ASP 8118	9	55.04	6.164
VAL 8122	9	63.14	THR 8125	9	63.72	5.155
VAL 8122	9	63.14	GLU 8126	9	101.94	6.172
VAL 8122	9	63.14	ASP 8128	9	39.05	9.854
TRP 8123	7	40.93	ARG 8117	9	59.48	9.708
TRP 8123	7	40.93	ASP 8118	9	55.04	8.63
TRP 8123	7	40.93	LEU 8121	8	64.06	5.393

TRP 8123	7	40.93	THR 8125	9	63.72	5.402
TRP 8123	7	40.93	GLU 8126	9	101.94	4.944
TRP 8123	7	40.93	ASP 8128	9	39.05	8.443
TRP 8123	7	40.93	LEU 8129	8	74.4	9.768
THR 8125	9	63.72	LEU 8121	8	64.06	6.215
THR 8125	9	63.72	VAL 8122	9	63.14	5.155
THR 8125	9	63.72	TRP 8123	7	40.93	5.402
THR 8125	9	63.72	ASP 8128	9	39.05	5.069
THR 8125	9	63.72	LEU 8129	8	74.4	6.13
THR 8125	9	63.72	GLN 8130	9	70.27	8.642
THR 8125	9	63.72	TYR 8195	8	86.86	8.965
GLU 8126	9	101.94	LEU 8121	8	64.06	8.553
GLU 8126	9	101.94	VAL 8122	9	63.14	6.172
GLU 8126	9	101.94	TRP 8123	7	40.93	4.944
GLU 8126	9	101.94	ASP 8128	9	39.05	5.437
GLU 8126	9	101.94	LEU 8129	8	74.4	5.166
GLU 8126	9	101.94	GLN 8130	9	70.27	6.276
ASP 8128	9	39.05	VAL 8122	9	63.14	9.854
ASP 8128	9	39.05	TRP 8123	7	40.93	8.443
ASP 8128	9	39.05	THR 8125	9	63.72	5.069
ASP 8128	9	39.05	GLU 8126	9	101.94	5.437
ASP 8128	9	39.05	GLN 8130	9	70.27	5.469
ASP 8128	9	39.05	THR 8132	9	53.66	6.139
ASP 8128	9	39.05	ASN 8133	9	59.73	8.932
ASP 8128	9	39.05	TYR 8195	8	86.86	8.599
ASP 8128	9	39.05	GLU 8198	9	60.81	8.579
ASP 8128	9	39.05	ARG 8202	9	83.81	8.105
LEU 8129	8	74.4	TRP 8123	7	40.93	9.768
LEU 8129	8	74.4	THR 8125	9	63.72	6.13
LEU 8129	8	74.4	GLU 8126	9	101.94	5.166
LEU 8129	8	74.4	THR 8132	9	53.66	5.323
LEU 8129	8	74.4	ASN 8133	9	59.73	6.792
GLN 8130	9	70.27	THR 8125	9	63.72	8.642
GLN 8130	9	70.27	GLU 8126	9	101.94	6.276
GLN 8130	9	70.27	ASP 8128	9	39.05	5.469
GLN 8130	9	70.27	THR 8132	9	53.66	5.464
GLN 8130	9	70.27	ASN 8133	9	59.73	5.633
GLN 8130	9	70.27	ILE 8134	9	75.49	8.876
GLN 8130	9	70.27	GLU 8135	9	76.97	9.812
GLN 8130	9	70.27	PHE 8152	8	42.52	9.003
GLN 8130	9	70.27	ARG 8202	9	83.81	9.86
THR 8132	9	53.66	ASP 8128	9	39.05	6.139
THR 8132	9	53.66	LEU 8129	8	74.4	5.323
THR 8132	9	53.66	GLN 8130	9	70.27	5.464
THR 8132	9	53.66	ILE 8134	9	75.49	6.215
THR 8132	9	53.66	GLU 8135	9	76.97	9.107
THR 8132	9	53.66	ARG 8202	9	83.81	7.752

GLU 8135	9	76.97	GLN 8130	9	70.27	9.812
GLU 8135	9	76.97	THR 8132	9	53.66	9.107
GLU 8135	9	76.97	ASN 8133	9	59.73	5.808
GLU 8135	9	76.97	PHE 8137	8	122.32	5.76
GLU 8135	9	76.97	SER 8138	9	58.85	8.444
GLU 8135	9	76.97	ASP 8141	9	66.29	8.475
GLU 8135	9	76.97	LEU 8213	8	39.33	9.881
PHE 8137	8	122.32	ILE 8134	9	75.49	8.498
PHE 8137	8	122.32	GLU 8135	9	76.97	5.76
PHE 8137	8	122.32	GLU 8139	9	152.31	6.798
PHE 8137	8	122.32	CYS 8140	8	78.69	8.8
PHE 8137	8	122.32	ASP 8141	9	66.29	6.989
PHE 8137	8	122.32	LEU 8213	8	39.33	7.677
PHE 8137	8	122.32	PRO 8214	8	36.93	9.046
SER 8138	9	58.85	GLU 8135	9	76.97	8.444
SER 8138	9	58.85	CYS 8140	8	78.69	5.369
SER 8138	9	58.85	ASP 8141	9	66.29	4.815
SER 8138	9	58.85	GLN 8143	9	103.1	8.479
SER 8138	9	58.85	ALA 8144	9	29.81	9.502
SER 8138	9	58.85	LEU 8213	8	39.33	8.522
SER 8138	9	58.85	PRO 8214	8	36.93	8.29
GLU 8139	9	152.31	PHE 8137	8	122.32	6.798
GLU 8139	9	152.31	ASP 8141	9	66.29	5.267
GLU 8139	9	152.31	GLN 8143	9	103.1	6.352
GLU 8139	9	152.31	ALA 8144	9	29.81	8.714
GLU 8139	9	152.31	LEU 8213	8	39.33	7.711
GLU 8139	9	152.31	PRO 8214	8	36.93	5.913
GLU 8139	9	152.31	LEU 8215	8	73.45	9.042
CYS 8140	8	78.69	PHE 8137	8	122.32	8.8
CYS 8140	8	78.69	SER 8138	9	58.85	5.369
CYS 8140	8	78.69	GLN 8143	9	103.1	5.373
CYS 8140	8	78.69	ALA 8144	9	29.81	6.723
CYS 8140	8	78.69	PRO 8214	8	36.93	8.787
ASP 8141	9	66.29	GLU 8135	9	76.97	8.475
ASP 8141	9	66.29	PHE 8137	8	122.32	6.989
ASP 8141	9	66.29	SER 8138	9	58.85	4.815
ASP 8141	9	66.29	GLU 8139	9	152.31	5.267
ASP 8141	9	66.29	GLN 8143	9	103.1	5.259
ASP 8141	9	66.29	ALA 8144	9	29.81	4.923
ASP 8141	9	66.29	ILE 8146	9	59	8.539
ASP 8141	9	66.29	LYS 8147	9	114.85	9.947
ASP 8141	9	66.29	PRO 8214	8	36.93	9.168
GLN 8143	9	103.1	SER 8138	9	58.85	8.479
GLN 8143	9	103.1	GLU 8139	9	152.31	6.352
GLN 8143	9	103.1	CYS 8140	8	78.69	5.373
GLN 8143	9	103.1	ASP 8141	9	66.29	5.259

GLN 8143	9	103.1	ILE 8146	9	59	5.274
GLN 8143	9	103.1	LYS 8147	9	114.85	6.584
GLN 8143	9	103.1	GLN 8148	9	96.16	8.716
GLN 8143	9	103.1	LEU 8213	8	39.33	9.8
GLN 8143	9	103.1	PRO 8214	8	36.93	7.283
ALA 8144	9	29.81	SER 8138	9	58.85	9.502
ALA 8144	9	29.81	GLU 8139	9	152.31	8.714
ALA 8144	9	29.81	CYS 8140	8	78.69	6.723
ALA 8144	9	29.81	ASP 8141	9	66.29	4.923
ALA 8144	9	29.81	ILE 8146	9	59	5.481
ALA 8144	9	29.81	LYS 8147	9	114.85	5.354
ALA 8144	9	29.81	GLN 8148	9	96.16	6.207
ILE 8146	9	59	ASP 8141	9	66.29	8.539
ILE 8146	9	59	GLN 8143	9	103.1	5.274
ILE 8146	9	59	ALA 8144	9	29.81	5.481
ILE 8146	9	59	GLN 8148	9	96.16	5.343
ILE 8146	9	59	PHE 8152	8	42.52	9.782
ILE 8146	9	59	PRO 8214	8	36.93	8.832
GLN 8148	9	96.16	GLN 8143	9	103.1	8.716
GLN 8148	9	96.16	ALA 8144	9	29.81	6.207
GLN 8148	9	96.16	ILE 8146	9	59	5.343
GLN 8148	9	96.16	PHE 8152	8	42.52	6.342
GLN 8148	9	96.16	GLN 8154	9	127.62	9.881
PHE 8152	8	42.52	GLN 8130	9	70.27	9.003
PHE 8152	8	42.52	ILE 8146	9	59	9.782
PHE 8152	8	42.52	LYS 8147	9	114.85	8.857
PHE 8152	8	42.52	GLN 8148	9	96.16	6.342
PHE 8152	8	42.52	GLN 8154	9	127.62	5.271
PHE 8152	8	42.52	GLU 8155	9	40.41	4.826
PHE 8152	8	42.52	LEU 8158	8	93.51	9.597
GLU 8175	9	61.44	GLU 8171	9	80.76	5.233
GLU 8175	9	61.44	GLU 8178	9	181.73	7.904
GLU 8175	9	61.44	ASP 8181	9	91.61	9.029
GLU 8175	9	61.44	ALA 8183	9	36.61	8.9
GLU 8175	9	61.44	GLU 8186	9	77.72	7.677
ASP 8181	9	91.61	GLU 8175	9	61.44	9.029
ASP 8181	9	91.61	GLU 8178	9	181.73	7.644
ASP 8181	9	91.61	PRO 8179	8	108.48	6.831
ASP 8181	9	91.61	ALA 8183	9	36.61	5.644
ASP 8181	9	91.61	GLU 8186	9	77.72	9.372
ALA 8183	9	36.61	GLU 8175	9	61.44	8.9
ALA 8183	9	36.61	ASP 8181	9	91.61	5.644
ALA 8183	9	36.61	GLU 8186	9	77.72	5.101
ALA 8183	9	36.61	GLU 8187	9	104.66	6.035
ALA 8183	9	36.61	LEU 8189	8	35.7	9.926

GLU 8186	9	77.72	GLU 8171	9	80.76	8.126
GLU 8186	9	77.72	GLU 8175	9	61.44	7.677
GLU 8186	9	77.72	ASP 8181	9	91.61	9.372
GLU 8186	9	77.72	ALA 8183	9	36.61	5.101
GLU 8186	9	77.72	LEU 8189	8	35.7	5.196
GLU 8186	9	77.72	GLU 8191	9	54.31	8.816
LEU 8189	8	35.7	ARG 8117	9	59.48	8.893
LEU 8189	8	35.7	GLU 8171	9	80.76	8.728
LEU 8189	8	35.7	ALA 8183	9	36.61	9.926
LEU 8189	8	35.7	GLU 8186	9	77.72	5.196
LEU 8189	8	35.7	GLU 8187	9	104.66	5.376
LEU 8189	8	35.7	GLU 8191	9	54.31	5.466
LEU 8189	8	35.7	ARG 8194	9	117.95	8.674
GLU 8191	9	54.31	LEU 8121	8	64.06	9.521
GLU 8191	9	54.31	GLU 8186	9	77.72	8.816
GLU 8191	9	54.31	GLU 8187	9	104.66	6.29
GLU 8191	9	54.31	LEU 8189	8	35.7	5.466
GLU 8191	9	54.31	ARG 8194	9	117.95	5.037
GLU 8191	9	54.31	TYR 8195	8	86.86	6.087
TYR 8195	8	86.86	LEU 8121	8	64.06	9.029
TYR 8195	8	86.86	THR 8125	9	63.72	8.965
TYR 8195	8	86.86	ASP 8128	9	39.05	8.599
TYR 8195	8	86.86	GLU 8191	9	54.31	6.087
TYR 8195	8	86.86	GLU 8198	9	60.81	5.008
TYR 8195	8	86.86	PHE 8200	8	56.26	8.788
GLU 8198	9	60.81	ASP 8128	9	39.05	8.579
GLU 8198	9	60.81	ARG 8194	9	117.95	6.237
GLU 8198	9	60.81	TYR 8195	8	86.86	5.008
GLU 8198	9	60.81	PHE 8200	8	56.26	5.387
GLU 8198	9	60.81	ARG 8202	9	83.81	6.122
ARG 8202	9	83.81	ASP 8128	9	39.05	8.105
ARG 8202	9	83.81	GLN 8130	9	70.27	9.86
ARG 8202	9	83.81	THR 8132	9	53.66	7.752
ARG 8202	9	83.81	GLU 8198	9	60.81	6.122
ARG 8202	9	83.81	PHE 8200	8	56.26	5.393
ARG 8202	9	83.81	ARG 8205	9	165.7	5.104
LEU 8213	8	39.33	GLU 8135	9	76.97	9.881
LEU 8213	8	39.33	PHE 8137	8	122.32	7.677
LEU 8213	8	39.33	SER 8138	9	58.85	8.522
LEU 8213	8	39.33	GLU 8139	9	152.31	7.711
LEU 8213	8	39.33	GLN 8143	9	103.1	9.8
LEU 8213	8	39.33	LEU 8215	8	73.45	4.997
PRO 8214	8	36.93	PHE 8137	8	122.32	9.046
PRO 8214	8	36.93	SER 8138	9	58.85	8.29
PRO 8214	8	36.93	GLU 8139	9	152.31	5.913
PRO 8214	8	36.93	CYS 8140	8	78.69	8.787

PRO 8214	8	36.93	ASP 8141	9	66.29	9.168
PRO 8214	8	36.93	GLN 8143	9	103.1	7.283
PRO 8214	8	36.93	ILE 8146	9	59	8.832
PRO 8214	8	36.93	ARG 8212	9	145.61	5.56

NES1-SR72

Cluster centre	Conservation	SASA (Å2)	neighbours	Conservation	SASA (Å2)	Distance Å
ARG 8358	7	77.45	THR 8361	8	61.66	5.71
ARG 8358	7	77.45	PRO 8362	7	47.27	4.935
ARG 8358	7	77.45	THR 8363	8	40.64	8.279
ARG 8358	7	77.45	GLY 8364	7	32.59	9.13
ARG 8358	7	77.45	PRO 8365	7	38.47	6.517
THR 8361	8	61.66	ARG 8358	7	77.45	5.71
THR 8361	8	61.66	SER 8359	8	126.36	6.522
THR 8361	8	61.66	THR 8363	8	40.64	6.259
THR 8361	8	61.66	GLY 8364	7	32.59	7.191
THR 8361	8	61.66	PRO 8365	7	38.47	6.892
PRO 8362	7	47.27	ARG 8358	7	77.45	4.935
PRO 8362	7	47.27	SER 8359	8	126.36	7.713
PRO 8362	7	47.27	GLY 8364	7	32.59	6.013
PRO 8362	7	47.27	PRO 8365	7	38.47	5.594
PRO 8362	7	47.27	LEU 8367	7	32.49	8.222
THR 8363	8	40.64	ARG 8358	7	77.45	8.279
THR 8363	8	40.64	THR 8361	8	61.66	6.259
THR 8363	8	40.64	PRO 8365	7	38.47	5.537
THR 8363	8	40.64	LEU 8367	7	32.49	6.076
THR 8363	8	40.64	ASP 8368	9	98.57	8.648
GLY 8364	7	32.59	ARG 8358	7	77.45	9.13
GLY 8364	7	32.59	THR 8361	8	61.66	7.191
GLY 8364	7	32.59	PRO 8362	7	47.27	6.013
GLY 8364	7	32.59	LEU 8367	7	32.49	5.24
GLY 8364	7	32.59	ASP 8368	9	98.57	6.377
PRO 8365	7	38.47	ARG 8358	7	77.45	6.517
PRO 8365	7	38.47	SER 8359	8	126.36	8.684
PRO 8365	7	38.47	THR 8361	8	61.66	6.892
PRO 8365	7	38.47	PRO 8362	7	47.27	5.594
PRO 8365	7	38.47	THR 8363	8	40.64	5.537
PRO 8365	7	38.47	LEU 8367	7	32.49	5.36
PRO 8365	7	38.47	ASP 8368	9	98.57	5.123
LEU 8367	7	32.49	PRO 8362	7	47.27	8.222
LEU 8367	7	32.49	THR 8363	8	40.64	6.076
LEU 8367	7	32.49	GLY 8364	7	32.59	5.24
LEU 8367	7	32.49	PRO 8365	7	38.47	5.36
LEU 8367	7	32.49	TYR 8371	8	82.25	6.297
LEU 8367	7	32.49	LYS 8372	7	110.65	8.603
LEU 8367	7	32.49	GLU 8433	8	60.17	9.437

ASP 8368	9	98.57	THR 8363	8	40.64	8.648
ASP 8368	9	98.57	GLY 8364	7	32.59	6.377
ASP 8368	9	98.57	PRO 8365	7	38.47	5.123
ASP 8368	9	98.57	TYR 8371	8	82.25	5.285
ASP 8368	9	98.57	LYS 8372	7	110.65	6.417
ASP 8368	9	98.57	TYR 8374	8	96.33	9.927
TYR 8371	8	82.25	LEU 8367	7	32.49	6.297
TYR 8371	8	82.25	ASP 8368	9	98.57	5.285
TYR 8371	8	82.25	TYR 8374	8	96.33	4.85
TYR 8371	8	82.25	MET 8375	9	58.25	6.22
TYR 8371	8	82.25	ALA 8429	7	34.48	9.776
ARG 8421	9	85.56	CYS 8381	7	57.11	8.951
ARG 8421	9	85.56	SER 8385	9	30.52	8.06
ARG 8421	9	85.56	ILE 8419	9	65.22	5.396
ARG 8421	9	85.56	GLU 8423	8	90.6	5.366
ARG 8421	9	85.56	ALA 8427	9	30.45	9.809
ALA 8427	9	30.45	THR 8353	7	56.89	9.094
ALA 8427	9	30.45	ARG 8421	9	85.56	9.809
ALA 8427	9	30.45	TRP 8422	7	115.23	8.565
ALA 8427	9	30.45	GLU 8423	8	90.6	5.956
ALA 8427	9	30.45	ALA 8429	7	34.48	5.445
ALA 8427	9	30.45	GLU 8433	8	60.17	9.633
ALA 8429	7	34.48	TYR 8371	8	82.25	9.776
ALA 8429	7	34.48	TYR 8374	8	96.33	8.488
ALA 8429	7	34.48	GLU 8423	8	90.6	9.792
ALA 8429	7	34.48	ALA 8427	9	30.45	5.445
ALA 8429	7	34.48	GLU 8433	8	60.17	6.047

NES1-SR73

Cluster centre	Conservation	SASA (Å2)	neighbours	Conservation	SASA (Å2)	Distance Å
ASP 8448	9	43.6	TRP 8442	7	53.08	9.78
ASP 8448	9	43.6	GLN 8444	9	114.26	6.284
ASP 8448	9	43.6	LEU 8500	8	35.73	9.459
ASP 8448	9	43.6	SER 8501	9	41.74	7.211
ASP 8448	9	43.6	ASN 8503	9	32.64	9.137
ILE 8578	9	43.64	THR 8473	7	105.98	7.3
ILE 8578	9	43.64	ASP 8474	9	47.54	6.247
ILE 8578	9	43.64	ILE 8475	9	82.89	5.197
ILE 8578	9	43.64	ILE 8482	9	35.52	6.367
ILE 8578	9	43.64	GLN 8546	9	88.53	9.79
ILE 8578	9	43.64	ALA 8548	9	97.27	8.851
ILE 8482	9	35.52	ILE 8478	9	43.64	6.367
ILE 8482	9	35.52	GLU 8479	9	114.13	5.377
ILE 8482	9	35.52	LYS 8486	9	107.26	5.969
ILE 8482	9	35.52	LEU 8488	8	33.84	9.589

ILE 8482	9	35.52	ARG 8542	9	126.66	8.33
ILE 8482	9	35.52	GLN 8546	9	88.53	7.635
ILE 8482	9	35.52	ALA 8548	9	97.27	9.934
LYS 8486	9	107.26	ILE 8482	9	35.52	5.969
LYS 8486	9	107.26	LEU 8488	8	33.84	5.202
LYS 8486	9	107.26	GLN 8489	9	50.95	4.963
LYS 8486	9	107.26	LYS 8490	9	144.47	6.153
LYS 8486	9	107.26	ARG 8542	9	126.66	8.258
LEU 8488	8	33.84	GLU 8462	9	51.95	8.233
LEU 8488	8	33.84	ILE 8482	9	35.52	9.589
LEU 8488	8	33.84	LYS 8483	9	107.26	5.202
LEU 8488	8	33.84	LYS 8490	9	144.47	5.2
LEU 8488	8	33.84	ASP 8493	9	95.66	8.704
LYS 8496	9	88.81	TRP 8455	7	62.89	9.296
LYS 8496	9	88.81	LYS 8490	9	144.47	9.839
LYS 8496	9	88.81	ASP 8493	9	95.66	5.024
LYS 8496	9	88.81	LEU 8500	8	35.73	6.018
LYS 8496	9	88.81	SER 8501	9	41.74	8.279
LYS 8496	9	88.81	TRP 8531	7	69.13	8.799
LEU 8500	8	35.73	ASP 8448	9	43.6	9.459
LEU 8500	8	35.73	LYS 8493	9	88.81	6.018
LEU 8500	8	35.73	ASN 8503	9	32.64	4.993
LEU 8500	8	35.73	LEU 8504	8	84.59	6.31
LEU 8500	8	35.73	ASN 8528	9	77.11	7.929
LEU 8500	8	35.73	TRP 8531	7	69.13	8.363
TRP 8531	7	69.13	TRP 8455	7	62.89	9.725
TRP 8531	7	69.13	GLU 8460	9	48.39	9.374
TRP 8531	7	69.13	LYS 8496	9	88.81	8.799
TRP 8531	7	69.13	LEU 8500	8	35.73	8.363
TRP 8531	7	69.13	ASN 8528	9	77.11	5.039
ARG 8542	9	126.66	ILE 8482	9	35.52	8.33
ARG 8542	9	126.66	LYS 8486	9	107.26	8.258
ARG 8542	9	126.66	GLN 8489	9	50.95	8.737
ARG 8542	9	126.66	GLN 8546	9	88.53	6.299
ARG 8542	9	126.66	ALA 8548	9	97.27	9.896
GLN 8546	9	88.53	ILE 8478	9	43.64	9.79
GLN 8546	9	88.53	ILE 8482	9	35.52	7.635
GLN 8546	9	88.53	TRP 8541	7	44.06	8.443
GLN 8546	9	88.53	ARG 8542	9	126.66	6.299
GLN 8546	9	88.53	ALA 8548	9	97.27	5.441

NES1-SR74

Cluster centre	Conservation	SASA (Å2)	neighbours	Conservation	SASA (Å2)	Distance Å
GLN 8551	9	57.64	LEU 8549	8	177.2	5.269
GLN 8551	9	57.64	GLN 8551	9	89.31	5.462

GLN 8551	9	57.64	GLN 8618	9	83.64	7.105
GLN 8551	9	57.64	LEU 8620	8	65.96	9.916
GLN 8551	9	57.64	GLY 8625	8	24.61	7.177
GLN 8553	9	89.31	LEU 8549	8	177.2	6.079
GLN 8553	9	89.31	MET 8550	9	154.98	5.01
GLN 8553	9	89.31	GLN 8551	9	57.64	5.462
GLN 8553	9	89.31	GLU 8557	9	128.71	6.183
GLN 8553	9	89.31	GLY 8625	8	24.61	9.248
GLN 8553	9	89.31	LEU 8629	8	135.87	9.093
LEU 8563	8	36	HIS 8560	9	76.05	5.552
LEU 8563	8	36	GLU 8568	9	110.66	6.44
LEU 8563	8	36	ASN 8568	9	92.37	8.712
LEU 8563	8	36	VAL 8635	9	59.98	9.974
LEU 8563	8	36	VAL 8637	9	37.07	8.203
LEU 8563	8	36	ARG 8641	9	73.27	7.839
LEU 8563	8	36	LEU 8642	8	50.21	8.908
GLU 8567	9	110.66	LEU 8563	8	36	6.44
GLU 8567	9	110.66	LEU 8564	8	72.02	5.035
GLU 8567	9	110.66	ARG 8571	9	148.58	6.588
GLU 8567	9	110.66	ARG 8572	9	123.86	8.951
GLU 8567	9	110.66	ARG 8641	9	73.27	7.563
GLU 8567	9	110.66	LEU 8642	8	50.21	9.166
ASN 8568	9	92.37	LEU 8563	8	36	8.712
ASN 8568	9	92.37	LEU 8564	8	72.02	5.953
ASN 8568	9	92.37	ARG 8571	9	148.58	5.212
ASN 8568	9	92.37	ARG 8572	9	123.86	6.482
ASN 8568	9	92.37	ASN 8574	9	61.99	9.792
ASN 8574	9	61.99	ASN 8568	9	92.37	9.792
ASN 8574	9	61.99	ARG 8571	9	148.58	4.799
ASN 8574	9	61.99	ARG 8572	9	123.86	5.318
ASN 8574	9	61.99	PRO 8578	9	36.38	8.682
ASN 8574	9	61.99	ILE 8579	9	125.28	9.008
GLN 8618	9	83.64	GLN 8551	9	57.64	7.105
GLN 8618	9	83.64	GLN 8613	9	121.47	8.812
GLN 8618	9	83.64	LEU 8620	8	65.96	5.211
GLN 8618	9	83.64	VAL 8621	9	93.78	5.097
GLN 8618	9	83.64	GLY 8625	8	24.61	8.672
GLY 8625	8	24.61	LEU 8549	8	177.2	7.729
GLY 8625	8	24.61	MET 8550	9	154.98	9.617
GLY 8625	8	24.61	GLN 8551	9	57.64	7.177
GLY 8625	8	24.61	GLN 8553	9	89.31	9.248
GLY 8625	8	24.61	GLN 8618	9	83.64	8.672
GLY 8625	8	24.61	LEU 8620	8	65.96	6.994
GLY 8625	8	24.61	VAL 8621	9	93.78	9.35
GLY 8625	8	24.61	LEU 8629	8	135.87	8.61

VAL 8635	9	59.98	LEU 8563	8	36	9.974
VAL 8635	9	59.98	VAL 8609	9	47.17	9.266
VAL 8635	9	59.98	GLN 8613	9	121.47	7.015
VAL 8635	9	59.98	VAL 8637	9	37.07	5.611
VAL 8635	9	59.98	ASN 8640	9	72.16	8.851
VAL 8637	9	37.07	LEU 8563	8	36	8.203
VAL 8637	9	37.07	VAL 8635	9	59.98	5.611
VAL 8637	9	37.07	ASN 8640	9	72.16	5.217
VAL 8637	9	37.07	ARG 8641	9	73.27	6.181
VAL 8637	9	37.07	LEU 8642	8	50.21	8.427
ARG 8641	9	73.27	LEU 8563	8	36	7.839
ARG 8641	9	73.27	GLU 8567	9	110.66	7.563
ARG 8641	9	73.27	HIS 8636	9	131.59	8.604
ARG 8641	9	73.27	VAL 8637	9	37.07	6.181
ARG 8641	9	73.27	LEU 8646	8	41.6	8.712
ARG 8641	9	73.27	LYS 8647	9	131.44	9.764
LEU 8642	8	50.21	LEU 8563	8	36	8.908
LEU 8642	8	50.21	GLU 8567	9	110.66	9.166
LEU 8642	8	50.21	VAL 8609	9	47.17	7.969
LEU 8642	8	50.21	HIS 8636	9	131.59	9.696
LEU 8642	8	50.21	VAL 8637	9	37.07	8.427
LEU 8642	8	50.21	ASN 8640	9	72.16	5.417
LEU 8642	8	50.21	LEU 8646	8	41.6	6.4
LEU 8642	8	50.21	LYS 8647	9	131.44	8.656
