# Supplementary material for: Large-Scale Modelling of the Divergent Spectrin Repeats in Nesprins: Giant Modular Proteins
Source: PLoS One. 2013 May 6;8(5):e63633. doi: 10.1371/journal.pone.0063633 (PMC3646009; doi:10.1371/journal.pone.0063633)
Supplement: Table S4 — Nesprin2β hot spots. (PDF) [file pone.0063633.s016.pdf]

Table S4: Nesprin2β hot spots

NES2-SR51

Cluster centre	Conservation	SASA (Å2	neighbours	Conservation	SASA (Å2)	Distance Å
ASN 6029	9	53.31	GLN 6024	8	48.28	8.672
ASN 6029	9	53.31	ASP 6027	8	47.27	5.399
ASN 6029	9	53.31	ASN 6032	9	53.36	5.356
ASN 6029	9	53.31	ARG 6034	8	150.92	8.51
ASN 6029	9	53.31	SER 6081	9	49.64	9.753
ARG 6034	8	150.92	LYS 6028	8	108.57	9.831
ARG 6034	8	150.92	ASN 6029	9	53.31	8.51
ARG 6034	8	150.92	ASN 6032	9	53.36	5.339
ARG 6034	8	150.92	THR 6108	9	33.15	9.858
ARG 6034	8	150.92	ASP 6113	8	39.96	9.708
ARG 6034	8	150.92	ARG 6115	8	102.34	7.501
TYR 6050	7	94.99	LYS 6046	8	99.21	8.39
TYR 6050	7	94.99	PRO 6047	8	102.17	5.514
TYR 6050	7	94.99	VAL 6048	9	51.38	5.414
TYR 6050	7	94.99	CYS 6053	7	118.61	8.707
TYR 6050	7	94.99	ARG 6127	8	145.74	9.93
TYR 6050	7	94.99	GLU 6131	8	61.09	8.832
TYR 6050	7	94.99	GLU 6132	8	101.53	8.202
TYR 6050	7	94.99	LEU 6136	7	103.92	9.586
LEU 6068	7	47.84	GLU 6043	8	85.29	8.814
LEU 6068	7	47.84	GLU 6064	8	61.06	6.071
LEU 6068	7	47.84	GLN 6066	8	86.07	5.37
LEU 6068	7	47.84	ASP 6071	8	59.56	5.148
LEU 6068	7	47.84	GLU 6073	8	113.27	8.325
ASP 6092	8	85.96	ASP 6094	8	62.84	5.341
ASP 6092	8	85.96	CYS 6096	7	63.29	8.813
ASP 6092	8	85.96	ASP 6103	8	101.51	9.76
ASP 6092	8	85.96	GLN 6106	8	74.76	6.829
ASP 6092	8	85.96	GLN 6107	8	136.46	9.999
ASP 6094	8	62.84	HIS 6091	9	56.7	4.897
ASP 6094	8	62.84	ASP 6092	8	85.96	5.341
ASP 6094	8	62.84	CYS 6096	7	63.29	5.097
ASP 6094	8	62.84	ASP 6103	8	101.51	8.935
ASP 6094	8	62.84	GLN 6106	8	74.76	8.985
ASP 6103	8	101.51	HIS 6091	9	56.7	9.609
ASP 6103	8	101.51	ASP 6092	8	85.96	9.76
ASP 6103	8	101.51	ASP 6094	8	62.84	8.935
ASP 6103	8	101.51	GLU 6101	8	48.84	5.412
ASP 6103	8	101.51	GLN 6106	8	74.76	5.274
ASP 6103	8	101.51	GLN 6107	8	136.46	6.846
ASP 6103	8	101.51	THR 6108	9	33.15	9.106
GLN 6106	8	74.76	HIS 6091	9	56.7	7.221

GLN 6106	8	74.76	ASP 6092	8	85.96	6.829
GLN 6106	8	74.76	ASP 6094	8	62.84	8.985
GLN 6106	8	74.76	GLU 6101	8	48.84	8.698
GLN 6106	8	74.76	ASP 6103	8	101.51	5.274
GLN 6106	8	74.76	SER 6104	9	49.99	5.175
GLN 6106	8	74.76	THR 6108	9	33.15	5.422
THR 6108	9	33.15	ASP 6027	8	47.27	8.097
THR 6108	9	33.15	ARG 6034	8	150.92	9.858
THR 6108	9	33.15	ASP 6103	8	101.51	9.106
THR 6108	9	33.15	SER 6104	9	49.99	6.388
THR 6108	9	33.15	GLN 6106	8	74.76	5.422
THR 6108	9	33.15	ASP 6113	8	39.96	8.618
ASP 6113	8	39.96	ARG 6034	8	150.92	9.708
ASP 6113	8	39.96	GLN 6107	8	136.46	9.848
ASP 6113	8	39.96	THR 6108	9	33.15	8.618
ASP 6113	8	39.96	ARG 6115	8	102.34	5.411
ASP 6113	8	39.96	ARG 6117	8	117.59	6.029
ASP 6113	8	39.96	ASN 6118	9	66.17	8.351
ARG 6127	8	145.74	TYR 6050	7	94.99	9.93
ARG 6127	8	145.74	GLN 6066	8	86.07	7.553
ARG 6127	8	145.74	GLU 6125	8	98.65	5.398
ARG 6127	8	145.74	GLU 6131	8	61.09	6.152
ARG 6127	8	145.74	GLU 6132	8	101.53	8.605
GLU 6131	8	61.09	TYR 6050	7	94.99	8.832
GLU 6131	8	61.09	GLN 6059	8	88.71	8.485
GLU 6131	8	61.09	ARG 6127	8	145.74	6.152
GLU 6131	8	61.09	ARG 6135	8	139.86	6.427
GLU 6131	8	61.09	LEU 6136	7	103.92	8.816

NES2-SR52

Cluster centre	Conservation	SASA (Å2	neighbours	Conservation	SASA (Å2)	Distance Å
TYR 6168	8	150.99	VAL 6166	9	105.96	7.035
TYR 6168	8	150.99	LYS 6172	9	84.48	6.34
TYR 6168	8	150.99	GLU 6173	9	100.75	8.641
TYR 6168	8	150.99	GLU 6174	9	44.24	9.927
TYR 6168	8	150.99	PHE 6242	8	60.8	6.982
THR 6169	9	71.17	VAL 6166	9	105.96	8.32
THR 6169	9	71.17	LYS 6172	9	84.48	5.025
THR 6169	9	71.17	GLU 6173	9	100.75	6.254
THR 6169	9	71.17	GLU 6174	9	44.24	8.769
THR 6169	9	71.17	PHE 6242	8	60.8	9.457
GLU 6174	9	44.24	TYR 6168	8	150.99	9.927
GLU 6174	9	44.24	THR 6169	9	71.17	8.769
GLU 6174	9	44.24	LYS 6172	9	84.48	5.557
GLU 6174	9	44.24	GLU 6179	9	68.05	8.35
GLU 6174	9	44.24	LEU 6236	8	31.73	9.878

GLU 6174	9	44.24	PHE 6242	8	60.8	9.567
GLN 6182	9	48.78	GLU 6179	9	68.05	4.966
GLN 6182	9	48.78	HIS 6186	9	145.82	6.152
GLN 6182	9	48.78	GLU 6187	9	106.44	8.445
GLN 6182	9	48.78	GLN 6229	9	71.71	9.237
GLN 6182	9	48.78	LEU 6236	8	31.73	8.6
GLU 6193	9	85.36	LEU 6189	8	59.72	6.416
GLU 6193	9	85.36	THR 6190	9	81.03	5.298
GLU 6193	9	85.36	ASN 6196	9	60.7	5.09
GLU 6193	9	85.36	GLN 6198	9	84.18	8.255
GLU 6193	9	85.36	ASN 6222	9	63.51	9.395
ASN 6196	9	60.7	GLU 6193	9	85.36	5.09
ASN 6196	9	60.7	GLN 6198	9	84.18	5.467
ASN 6196	9	60.7	ARG 6200	9	189.39	6.594
ASN 6196	9	60.7	ARG 6201	9	188.41	9.074
ASN 6196	9	60.7	HIS 6219	9	111.54	8.789
ASN 6196	9	60.7	ASN 6222	9	63.51	7.449
SER 6212	9	52.3	ASN 6206	9	43.49	8.661
SER 6212	9	52.3	ARG 6207	9	261.39	7.202
SER 6212	9	52.3	THR 6208	9	42.75	6.198
SER 6212	9	52.3	ASP 6209	9	121.61	4.817
SER 6212	9	52.3	MET 6217	9	68.43	8.578
HIS 6219	9	111.54	ASN 6196	9	60.7	8.789
HIS 6219	9	111.54	ARG 6200	9	189.39	9.912
HIS 6219	9	111.54	MET 6217	9	68.43	5.299
HIS 6219	9	111.54	ASN 6222	9	63.51	5.278
HIS 6219	9	111.54	GLN 6223	9	80.77	6.422
ASN 6222	9	63.51	GLU 6193	9	85.36	9.395
ASN 6222	9	63.51	ASN 6196	9	60.7	7.449
ASN 6222	9	63.51	MET 6217	9	68.43	8.851
ASN 6222	9	63.51	HIS 6219	9	111.54	5.278
ASN 6222	9	63.51	ASP 6226	9	64.22	6.276
GLN 6229	9	71.71	GLN 6182	9	48.78	9.237
GLN 6229	9	71.71	HIS 6186	9	145.82	9.757
GLN 6229	9	71.71	LEU 6189	8	59.72	9.998
GLN 6229	9	71.71	GLN 6223	9	80.77	9.939
GLN 6229	9	71.71	ASP 6226	9	64.22	5.283
GLN 6229	9	71.71	ARG 6231	9	141.29	5.46
GLN 6229	9	71.71	ALA 6234	9	40.87	8.63
ARG 6231	9	141.29	ALA 6159	9	40.68	7.152
ARG 6231	9	141.29	ASP 6226	9	64.22	8.687
ARG 6231	9	141.29	GLN 6229	9	71.71	5.46
ARG 6231	9	141.29	ALA 6234	9	40.87	5.108

ARG 6231	9	141.29	LEU 6236	8	31.73	8.35
LEU 6236	8	31.73	GLU 6174	9	44.24	9.878
LEU 6236	8	31.73	GLU 6179	9	68.05	8.327
LEU 6236	8	31.73	GLN 6182	9	48.78	8.6
LEU 6236	8	31.73	ARG 6231	9	141.29	8.35
LEU 6236	8	31.73	ALA 6234	9	40.87	5.373
LEU 6236	8	31.73	ARG 6238	9	92.59	5.402
PHE 6242	8	60.8	TYR 6168	8	150.99	6.982
PHE 6242	8	60.8	THR 6169	9	71.17	9.457
PHE 6242	8	60.8	LYS 6172	9	84.48	7.467
PHE 6242	8	60.8	GLU 6174	9	44.24	9.567
PHE 6242	8	60.8	ARG 6238	9	92.59	8.391

NES2-SR53

Cluster centre	Conservation	SASA (Å2	neighbours	Conservation	SASA (Å2)	Distance Å
LEU 6257	8	46.35	THR 6261	9	36.65	6.097
LEU 6257	8	46.35	GLU 6262	9	118.24	8.595
LEU 6257	8	46.35	GLU 6324	9	63.04	9.011
LEU 6257	8	46.35	GLU 6327	9	71.07	8.721
LEU 6257	8	46.35	TYR 6331	8	89.01	8.744
THR 6261	9	36.65	LEU 6257	8	46.35	6.097
THR 6261	9	36.65	VAL 6258	9	46.8	5.087
THR 6261	9	36.65	LEU 6265	8	92.36	6.378
THR 6261	9	36.65	GLN 6266	9	83.25	8.848
THR 6261	9	36.65	TYR 6331	8	89.01	8.065
GLU 6262	9	118.24	LEU 6257	8	46.35	8.595
GLU 6262	9	118.24	VAL 6258	9	46.8	6.173
GLU 6262	9	118.24	LEU 6265	8	92.36	5.02
GLU 6262	9	118.24	GLN 6266	9	83.25	6.367
GLU 6262	9	118.24	THR 6268	9	61.02	9.762
THR 6268	9	61.02	GLU 6262	9	118.24	9.762
THR 6268	9	61.02	LEU 6265	8	92.36	4.979
THR 6268	9	61.02	GLN 6266	9	83.25	5.502
THR 6268	9	61.02	GLU 6271	9	103.07	9.898
THR 6268	9	61.02	ARG 6338	9	144.83	7.874
LYS 6298	9	86.95	THR 6293	9	75.68	9.569
LYS 6298	9	86.95	LEU 6294	8	106.9	7.755
LYS 6298	9	86.95	ASP 6300	9	69.61	5.403
LYS 6298	9	86.95	ILE 6303	9	50.52	8.613
LYS 6298	9	86.95	VAL 6304	9	61.85	9.993
ILE 6303	9	50.52	LYS 6298	9	86.95	8.613
ILE 6303	9	50.52	ASP 6300	9	69.61	5.118
ILE 6303	9	50.52	GLU 6307	9	90.61	6.278
ILE 6303	9	50.52	LEU 6309	8	41.89	9.957
ILE 6303	9	50.52	GLU 6326	9	67.63	8.821

GLU 6307	9	90.61	ILE 6303	9	50.52	6.278
GLU 6307	9	90.61	VAL 6304	9	61.85	5.314
GLU 6307	9	90.61	LEU 6309	8	41.89	5.474
GLU 6307	9	90.61	GLN 6311	9	83.97	5.868
GLU 6307	9	90.61	GLU 6322	9	49.68	7.759
GLU 6307	9	90.61	GLU 6326	9	67.63	9.877
LEU 6309	8	41.89	ARG 6246	9	190.12	7.719
LEU 6309	8	41.89	ILE 6303	9	50.52	9.957
LEU 6309	8	41.89	VAL 6304	9	61.85	8.86
LEU 6309	8	41.89	GLU 6307	9	90.61	5.474
LEU 6309	8	41.89	GLN 6311	9	83.97	5.618
LEU 6309	8	41.89	SER 6313	9	39.32	7.442
LEU 6309	8	41.89	GLU 6322	9	49.68	9.052
GLU 6322	9	49.68	GLU 6307	9	90.61	7.759
GLU 6322	9	49.68	LEU 6309	8	41.89	9.052
GLU 6322	9	49.68	GLN 6311	9	83.97	9.147
GLU 6322	9	49.68	SER 6313	9	39.32	9.798
GLU 6322	9	49.68	ASP 6317	9	83.34	8.583
GLU 6322	9	49.68	GLU 6324	9	63.04	5.36
GLU 6322	9	49.68	GLU 6326	9	67.63	6.376
GLU 6322	9	49.68	GLU 6327	9	71.07	8.861
GLU 6326	9	67.63	ILE 6303	9	50.52	8.821
GLU 6326	9	67.63	GLU 6307	9	90.61	9.877
GLU 6326	9	67.63	GLU 6322	9	49.68	6.376
GLU 6326	9	67.63	GLU 6324	9	63.04	5.345
GLU 6326	9	67.63	TYR 6331	8	89.01	8.586
GLU 6327	9	71.07	LEU 6257	8	46.35	8.721
GLU 6327	9	71.07	GLU 6322	9	49.68	8.861
GLU 6327	9	71.07	GLU 6324	9	63.04	5.163
GLU 6327	9	71.07	TYR 6331	8	89.01	5.909
GLU 6327	9	71.07	GLN 6333	9	127.95	9.887
TYR 6331	8	89.01	LEU 6257	8	46.35	8.744
TYR 6331	8	89.01	THR 6261	9	36.65	8.065
TYR 6331	8	89.01	GLU 6326	9	67.63	8.586
TYR 6331	8	89.01	GLU 6327	9	71.07	5.909
TYR 6331	8	89.01	GLN 6333	9	127.95	5.384
TYR 6331	8	89.01	GLU 6334	9	66.77	5.025
TYR 6331	8	89.01	PHE 6336	8	108	8.557
PHE 6336	8	108	THR 6293	9	75.68	9.657
PHE 6336	8	108	TYR 6331	8	89.01	8.557
PHE 6336	8	108	GLN 6333	9	127.95	5.456
PHE 6336	8	108	GLU 6334	9	66.77	5.47
PHE 6336	8	108	ARG 6338	9	144.83	5.414
PHE 6336	8	108	ARG 6341	9	185.3	8.788
ARG 6338	9	144.83	THR 6268	9	61.02	7.874

ARG 6338	9	144.83	GLN 6333	9	127.95	8.736
ARG 6338	9	144.83	GLU 6334	9	66.77	6.073
ARG 6338	9	144.83	PHE 6336	8	108	5.414
ARG 6338	9	144.83	ARG 6341	9	185.3	5.242
ARG 6338	9	144.83	HIS 6343	9	90.67	8.531
HIS 6343	9	90.67	MET 6282	9	67.03	7.972
HIS 6343	9	90.67	ARG 6338	9	144.83	8.531
HIS 6343	9	90.67	ARG 6341	9	185.3	5.433
HIS 6343	9	90.67	ARG 6345	9	158	5.419
HIS 6343	9	90.67	SER 6348	9	73.84	8.753

NES2-SR56

Cluster centre	Conservation	SASA (Å2	neighbours	Conservation	SASA (Å2)	Distance Å
LEU 6682	8	74.61	LEU 6678	8	110.32	6.407
LEU 6682	8	74.61	CYS 6685	8	37.9	4.894
LEU 6682	8	74.61	GLN 6686	7	96.65	5.798
LEU 6682	8	74.61	ASP 6687	7	62.63	8.575
LEU 6682	8	74.61	SER 6749	8	33.14	9.814
CYS 6685	8	37.9	LEU 6682	8	74.61	4.894
CYS 6685	8	37.9	ASP 6687	7	62.63	5.541
CYS 6685	8	37.9	HIS 6689	9	63.68	6.915
CYS 6685	8	37.9	LEU 6745	8	33.49	8.624
CYS 6685	8	37.9	GLN 6746	7	49.26	8.684
CYS 6685	8	37.9	ILE 6748	8	79.45	9.907
CYS 6685	8	37.9	SER 6749	8	33.14	6.877
CYS 6685	8	37.9	ASP 6760	7	81.14	8.415
LYS 6703	7	84.19	TRP 6698	7	65.45	8.331
LYS 6703	7	84.19	ARG 6705	7	137.42	5.495
LYS 6703	7	84.19	LYS 6774	9	105.7	9.463
LYS 6703	7	84.19	LEU 6775	8	64.74	9.538
LYS 6703	7	84.19	GLN 6777	7	84.8	7.489
LEU 6745	8	33.49	CYS 6685	8	37.9	8.624
LEU 6745	8	33.49	ASP 6687	7	62.63	8.491
LEU 6745	8	33.49	GLU 6747	7	81.79	5.224
LEU 6745	8	33.49	ILE 6748	8	79.45	4.915
LEU 6745	8	33.49	SER 6749	8	33.14	5.821
GLN 6746	7	49.26	CYS 6685	8	37.9	8.684
GLN 6746	7	49.26	ILE 6748	8	79.45	5.75
GLN 6746	7	49.26	SER 6749	8	33.14	5.421
GLN 6746	7	49.26	ASP 6760	7	81.14	9.673
GLN 6746	7	49.26	VAL 6768	7	51	7.662
GLN 6746	7	49.26	HIS 6769	8	149.61	9.906
SER 6749	8	33.14	LEU 6682	8	74.61	9.814
SER 6749	8	33.14	CYS 6685	8	37.9	6.877
SER 6749	8	33.14	LEU 6745	8	33.49	5.821
SER 6749	8	33.14	GLN 6746	7	49.26	5.421

SER 6749	8	33.14	GLU 6747	7	81.79	5.236
SER 6749	8	33.14	GLY 6758	8	38.98	9.726
SER 6749	8	33.14	GLU 6759	7	178.29	9.582
SER 6749	8	33.14	ASP 6760	7	81.14	8.254
LYS 6767	9	58.75	GLU 6763	9	63.54	6.565
LYS 6767	9	58.75	ALA 6764	9	59.09	5.186
LYS 6767	9	58.75	HIS 6769	8	149.61	5.514
LYS 6767	9	58.75	VAL 6770	9	41.67	4.937
LYS 6767	9	58.75	LYS 6774	9	105.7	9.58
VAL 6770	9	41.67	ALA 6764	9	59.09	9.453
VAL 6770	9	41.67	LYS 6767	9	58.75	4.937
VAL 6770	9	41.67	VAL 6768	7	51	5.69
VAL 6770	9	41.67	LYS 6774	9	105.7	6.435
VAL 6770	9	41.67	LEU 6775	8	64.74	8.772
VAL 6770	9	41.67	LYS 6776	7	68.56	9.551
LYS 6774	9	105.7	LEU 6697	8	120.79	9.922
LYS 6774	9	105.7	TRP 6698	7	65.45	9.991
LYS 6774	9	105.7	LYS 6703	7	84.19	9.463
LYS 6774	9	105.7	LYS 6767	9	58.75	9.58
LYS 6774	9	105.7	HIS 6769	8	149.61	9.613
LYS 6774	9	105.7	VAL 6770	9	41.67	6.435
LYS 6774	9	105.7	LYS 6776	7	68.56	5.29
LYS 6774	9	105.7	GLN 6777	7	84.8	5.014
